# 100% Fruit juice intake and cardiovascular risk: a systematic review and meta-analysis of prospective and randomised controlled studies

**DOI:** 10.1007/s00394-020-02426-7

**Published:** 2020-11-04

**Authors:** Lanfranco D’Elia, Monica Dinu, Francesco Sofi, Massimo Volpe, Pasquale Strazzullo, The SINU Working Group, Endorsed by SIPREC, Alessandra Bordoni, Pasquale Strazzullo, Giulia Cairella, Maria Cristina Casiraghi, Lanfranco D’Elia, Valeria del Balzo, Monica Dinu, Daniela Erba, Francesca Garbagnati, Andrea Ghiselli, Nicoletta Pellegrini, Alessandro Pinto, Laura Rossi, Gian Luigi Russo, Francesca Scazzina, Umberto Scognamiglio, Francesco Sofi, Salvatore Vaccaro, Elvira Verduci

**Affiliations:** 1 Department of Clinical Medicine and Surgery, ESH Excellence Centre of Hypertension, “Federico II” University of Naples Medical School, Via S. Pansini, 5. 80131, Naples, Italy; 2 Department of Experimental and Clinical Medicine, University of Florence, Florence, Italy; 3 Department of Clinical and Molecular Medicine, School of Medicine and Psychology, Sapienza University of Rome, Rome, Italy; 4 IRCCS Neuromed, Pozzilli, IS Italy

**Keywords:** Fruit juice, Cardiovascular disease, Cardiovascular risk factors, Blood pressure, Meta-analysis

## Abstract

**Purpose:**

The relationship between 100% fruit juice (100%FJ) consumption and cardiovascular risk is object of debate: indeed, recently published investigations provided new but discrepant evidence on this important question and International dietary guidelines are not in agreement on recommendations about fruit juice consumption. Therefore, we performed a meta-analysis of the prospective studies and the randomised controlled trials (RCTs) that explored the relationship between 100%FJ intake, cardiovascular risk profile and risk of cardiovascular events.

**Methods:**

We performed a systematic search of publications up to August 2019. Summary relative risks and exploration of linearity of the association were estimated for prospective studies and summary mean differences (MDs) calculated for RCTs.

**Results:**

A total of 21 prospective studies and 35 RCTs met the inclusion criteria. Dose–response analysis detected a significant inverse association between low-moderate 100%FJ consumption and risk of stroke (up to 200 ml/day) or total CV events (up to 170 ml/day) compared with no consumption, with a non-linear relationship (*p* for non-linearity < 0.05). No significant association was found for coronary heart disease and diabetes risk. In RCTs, a favorable and significant effect of 100%FJ intake was detected on blood pressure (systolic, MD: − 3.14 mmHg; diastolic, MD: − 1.68 mmHg), arterial compliance (carotid-femoral pulse wave velocity, − 0.38 m/s) and endothelial function (flow-mediated dilation, 2.10%). Neutral effects were found on body weight, blood lipids and glucose metabolism.

**Conclusions:**

The results of these analyses indicate that 100%FJ consumption is not associated with higher CV risk. A non-linear inverse dose–response relationship occurs between 100%FJ consumption and CV disease, in particular for risk of stroke, probably mediated by the decrease in blood pressure.

**Trial registration:**

PROSPERO registration number (CRD42019135577).

**Electronic supplementary material:**

The online version of this article (10.1007/s00394-020-02426-7) contains supplementary material, which is available to authorized users.

## Introduction

The pivotal role of fresh fruits and vegetables in a healthy diet is universally acknowledged and increased consumption of these foods is recommended by all the international guidelines for prevention of cardiovascular (CV) disease [1, 2]. On the other hand, the role of processed fruits, and in particular of 100% fruit juice (100%FJ), is object of debate because of its reduced nutritional value compared to fresh fruit mainly due to the generally lower fibre content and to the higher caloric density. However, 100%FJ provides substantial amounts of micronutrients and bioactive substances, among which polyphenols, minerals and vitamins. These nutrients may reduce oxidative stress and improve inflammatory markers, glucose metabolism [3, 4] and endothelial function [5], while inhibiting platelet aggregation [6]. For these reasons [7, 8], several studies were performed to analyse their possible influence on CV disorders, a major cause of death worldwide [9, 10]. While some epidemiological studies suggested that consumption of fruit drinks may have beneficial effects on CV risk [11–14], others provided contrasting evidence [15–17]. Unfortunately, most studies were not able to specify the exact properties of the fruit drinks consumed, i.e., whether 100%FJ or unspecified fruit drinks, and none explored the possibility of non-linear associations. In addition to prospective observational studies, many intervention trials were carried out to investigate the potential effect of fruit juice intake (mostly 100%FJ) on CV risk profile and many of them have been the object of various systematic reviews. One of these suggested a beneficial effect on diastolic blood pressure (BP) but, including several studies classified as low quality [18]. Other meta-analyses were limited to a single type of fruit or combined the effects of 100%FJ and fruit extracts [19, 20] or included of non-randomised controlled trials [21, 22].

The aim of the present study was thus to perform a comprehensive systematic review and subsequent meta-analysis of the prospective studies and the randomised controlled trials (RCTs) that explored the relationship between 100%FJ intake, CV risk profile and risk of CV events, aiming at clarifying the shape and strength of the dose–response relationship for these associations, if at all possible. As for prospective investigations the majority did not specify the type of fruit drink consumed by participants, we performed separate analyses of the few studies dealing with 100%FJ only but for completeness we also added an analysis including all the other studies available.

## Methods

### Data sources and search strategy

This meta-analysis was planned, conducted and reported according to the PRISMA statement [23]. The study protocol was preregistered (CRD42019135577). A systematic search of the available publications was performed using MEDLINE/PubMed, Web of Science, and Scopus, up to May 2019. The search was later updated to August 2019. The search strategy, without restrictions, included the expressions “fruit juice” AND “cardiovascular” OR “cerebrovascular” OR “stroke” OR “coronary heart” OR “blood pressure” OR “hypertension” OR “lipid” OR “cholesterol” OR “triglyceride” OR “HDL” OR “LDL” OR “glucose” OR “glycemia” OR “insulin” OR “HOMA” OR “weight” OR “BMI” OR “waist” OR “diabetes” OR “carotid” OR “flow mediated dilation” OR “pulse wave velocity” OR “arterial compliance” OR “arterial elasticity”, or combinations thereof, either in medical subject headings or in the title/abstract. Further information was retrieved through a manual search of references from recent reviews and relevant published original studies.

### Study selection and data extraction

The data selection was conducted and reported in accordance with the PRISMA statement [23] by L.D., and was checked for accuracy by M.D. The titles and abstracts of the studies retrieved in the searches were screened to identify the studies that met the predefined inclusion criteria. The full texts of the potentially eligible studies were then retrieved and assessed for eligibility. Discrepancies over the inclusion of studies and the interpretation of data were resolved in conference with a third reviewer (P.S.). Data were then extracted from the studies selected for inclusion by L.D. in accordance with the PRISMA statement, and was checked for accuracy by M.D.

### Inclusion criteria

To be included in the meta-analysis a published study had to meet the following criteria, stratified by study design:

*Prospective studies* (a) Original articles, (b) studies with a prospective design, (c) studies involving the adult population, (d) studies involving the assessment of 100% fruit juice (100%FJ) [24] or of unspecified fruit drinks intake as the baseline exposure, (e) studies in which the patients have a diagnosis of cardiovascular (CV) disease (i.e., total cardiovascular events/mortality, coronary heart disease, stroke events/mortality) and/or are exposed to CV risk factors (e.g., hypertension, diabetes) which are determined prospectively as outcomes, (f) studies in which there is an indication of the number of participants exposed and the rate or number of events in the different categories of 100% FJ intake or of unspecified fruit drinks intake, (g) studies in which there is an assessment of relative risk (RR) or hazard ratio (HR) for specified 100% FJ or of unspecified fruit drink intake categories, (h) studies with a follow-up of at least 2 years (mean or median).

*Intervention studies* (a) Original articles, (b) randomized controlled trials (RCTs), (c) studies involving the adult population, (d) studies in which there is an indication of the difference in outcomes—among which CV risk factors (e.g., blood pressure, lipid profile, glucose homeostasis, body weight) or CV damage (e.g., arterial stiffness, intima media thickness, flow-mediated dilation)—between the intake of 100% FJ [24] and of control drink in one or more patient cohorts; (e) studies in which there are indications of the number of participants included in the exposed and control groups; (f) studies in which the length of intervention is at least 7 days.

### Risk of bias

The risk of bias of the studies included in the meta-analyses was assessed according to established criteria [25]: the Newcastle–Ottawa Scale was used for the evaluation of prospective studies [26], and the Cochrane risk of bias tool was applied for the evaluation of randomized controlled trials (RCTs) [27].

### Grading of evidence

The quality of the entire body of evidence was evaluated using the GRADE (grading of recommendations assessment, development, and evaluation) methodology [28]. Evidence was graded as high, moderate or low quality. Observational studies started as low and RCTs as high by default. They were downgraded or upgraded based on specified criteria. Criteria to downgrade included study limitations (risk of bias), inconsistency (substantial unexplained heterogeneity), indirectness (factors that limit generalizability), imprecision (95% CI cross a minimally important difference of 5%, and publication bias (significant evidence of small-study effects). Criteria to upgrade certainty of evidence included a large magnitude of effect, a dose–response gradient, and attenuation by plausible confounding factors.

### Statistical analysis

Meta-analysis on the prospective evaluation: the assessment of linear and non-linear association between 100%FJ or unspecified fruit drink consumption and outcomes was carried out. The possibility of non-linear relationship was explored by modelling 100% FJ or unspecified fruit drink consumption using restricted cubic splines with three knots at fixed percentiles (25, 50, and 75%) of 100% FJ distribution. Departure from linearity was assessed by testing the null hypothesis that the coefficient of the second spline was equal to zero. A two-stage dose–response random-effects meta-analysis was performed [29, 30], which takes into account the correlation between the RR estimates across categories of 100% FJ or of unspecified fruit drink consumption. This analysis was carried out both to evaluate the effect in the single studies and to aggregate the results of all the studies. The median consumption for each specific category was assigned to each corresponding RR estimate. If the median consumption was not reported by the authors, the midpoint between the upper and lower boundary was used. If the lowest category was open-ended, its lower boundary was set to zero. If the upper boundary of the highest category was left unspecified, we assumed the category to be of the same amplitude as the preceding one. Statistical heterogeneity across the studies was also explored by *Q*-test.

Due to the small number of studies included in the prospective evaluation (*n* < 10), sensitivity, publication bias, sub-group and meta-regression analysis were not performed.

Further analyses were carried out including unspecified fruit drink study results. Separate dose–response analysis for unspecified fruit drink consumption was performed for stroke risk, coronary heart disease and diabetes risk.

For the meta-analysis of RCTs: mean differences (MDs)—and standard errors (SEs)—of the defined outcomes were extracted from the selected publications. The pooled weighted MD and 95% CI were estimated using a random-effect model. If these were not available, MD and SE were calculated from the comparison of the outcomes of 100% FJ and control drink intake [31]. The influence of a single cohort or of a particular study was estimated by sensitivity analysis. The Cochran *Q* test and the *I*^2^ statistic were used to evaluate statistical heterogeneity across the studies. Funnel plots were constructed and visually assessed for possible publication bias. Egger’s weighted regression test and Begg’s rank correlation test were also used to explore potential publication bias. In the case of significant funnel plot asymmetry, suggesting a number of possibly “missing” publications, the pooled estimate was recalculated based on the estimated number of “missing” studies and their effect sizes and SEs, a method known as “trim and fill”.

Subgroup and meta-regression analyses were used to identify associations between risk of cardiovascular risk factors and relevant study characteristics as possible sources of heterogeneity: total participants, age, gender, BMI, country, year of publication, length of intervention, underlying disease status, baseline value of the outcome and variables potentially involved, serving size, type of 100% FJ, study design, comparator, level of feeding control, energy intake, energy difference between intervention and comparator, domains of risk of bias, and assessment methods for arterial stiffness. The additional analyses were not performed if there was a small number of cohorts for single outcome exploration (*n* < 10).

All statistical analyses were performed using the Stata Corp. software (version11.2; College Station, Texas, USA).

## Results

Of a total of 16,762 publications retrieved, 21 prospective studies (8 reporting 100%FJ and 13 unspecified fruit drinks) [11–17, 32–45] (Online Resource-Supplemental-Tables 4–5) and 35 RCTs [46–81] (Online Resource-Supplemental Table 6) were identified that met the inclusion criteria (Online Resource-Supplemental Fig. 1, Online Resource-Supplemental Table 7).

*Prospective studies* Of 21 prospective studies included in the meta-analysis: 10 studies reported data on CV risk [11–17, 32, 34] (Online Resource-Supplemental Table 4), nine studies reported the risk only for diabetes [36, 38–45], one study only for hypertension [37], and finally one study both for diabetes and hypertension, separately [35] (Online Resource-Supplemental Table 5).

*Cardiovascular risk* A total of ten studies were included in the meta-analysis of CV risk assessment [11–17, 32, 34]. One of the studies reported the results of three cohorts stratified by percentage of dietary energy from carbohydrates, hence we considered these cohorts separately [15]. Four studies reported analyses relative to the same cohorts (Nurses’ Health Study-NHS and Health Professionals’ Follow-Up Study-HPFS), but focusing on different outcomes. Thus, each study was used for the included in only one analysis of the respective outcome. Only three studies specified the results for 100%FJ consumption [13, 14, 17]. All of them assessed 100%FJ consumption by a validated structured self-administered food frequency questionnaire according to the country meal pattern, containing specific questions on the average and type of fruit drinks consumption (EPIC [13, 14], Block [17]). In all the studies, the results were adjusted for the main confounders in multivariate models. All the studies had substantial high quality scores (Online Resource-Supplemental Table 5).

Diabetes risk. A total of ten studies were included in the meta-analysis of diabetes risk evaluation [35, 36, 38–45]. Only five studies specified the results for 100%FJ consumption [35, 38, 42, 44, 45]. Three of them [38, 42, 45] assessed 100%FJ consumption by a validated structured self-administered food frequency questionnaire according to the country meal pattern, containing specific questions on the average and type of fruit drinks consumption (EPIC); while in the other two studies a validated structured food frequency questionnaire containing specific items on type and serving size of fruit drink intake was administered to all participants (Women’s Health Initiative food frequency questionnaire [35], reduced version of Block [44]). In all but one study [44], the results were adjusted for the main confounders in multivariate models. All but one study had substantial high quality scores (Online Resource-Supplemental Table 5).

Hypertension risk. Only two studies were included in the analysis of hypertension development, but their risk expression was not comparable [35, 37] (Online Resource-Supplemental Table 5). These two studies assessed 100%FJ intake by a validated food frequency questionnaire containing specific items on type and serving size of fruit drink intake administered to all participants (Women’s Health Initiative food frequency questionnaire [35], Cardia dietary history [37]). One study included young participants with stringent criteria for diagnosis of hypertension [37]. The other one included post-menopausal women with common criteria for hypertension diagnosis [35].

*Quality of body of evidence* According to the GRADE criteria the evidence for the association between 100%FJ consumption and CV risk was of moderate quality both for risk of CV events and diabetes risk. Despite the GRADE methodology defines observational evidence from cohort studies as low quality, there was an upgrade of the score due to dose–response gradient.

### Risk of total cardiovascular disease

**Fig. 1:**
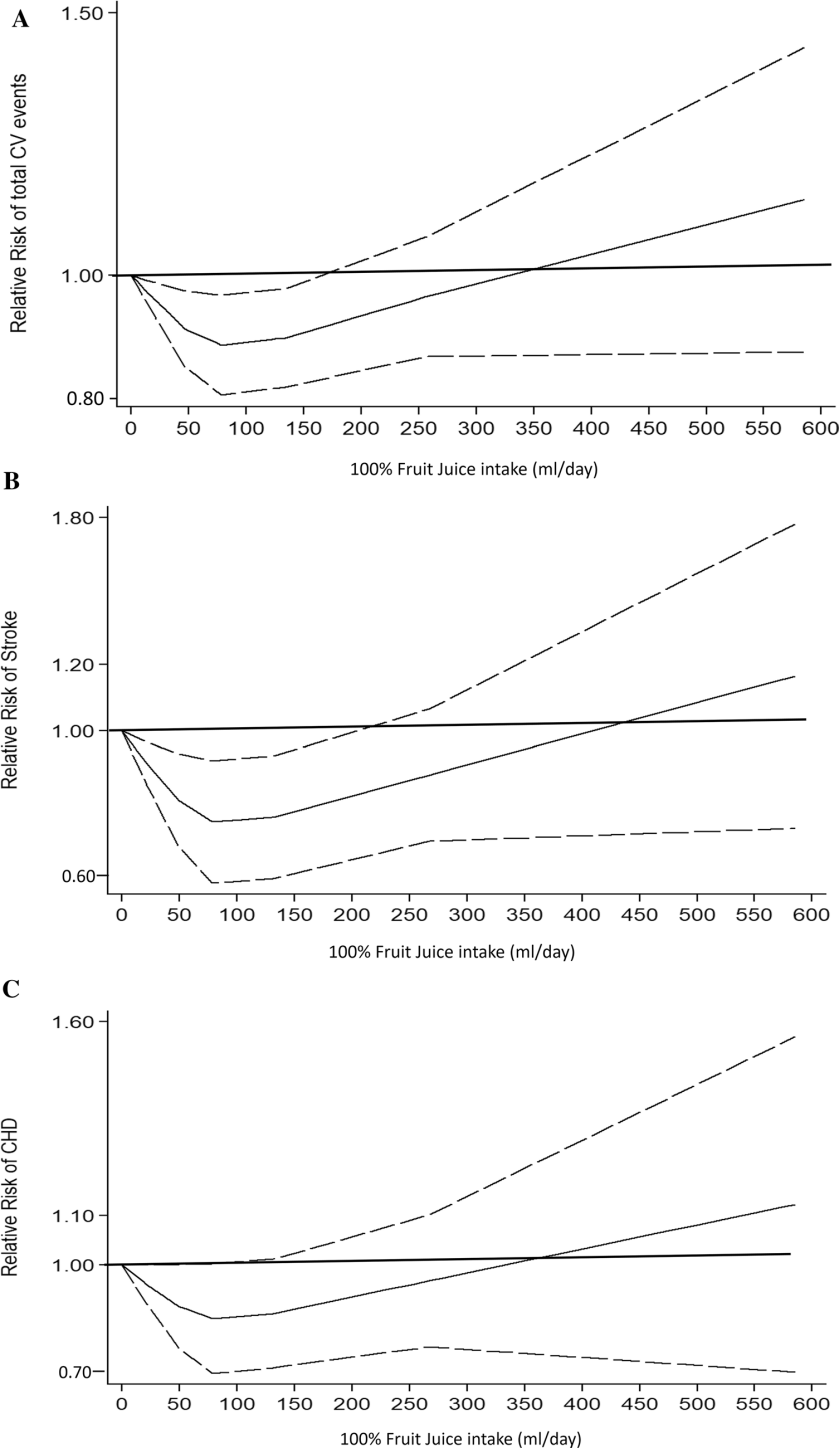
Dose–response association between 100% fruit juice consumption and cardiovascular events. **a** Risk of total cardiovascular (CV) events. **b** Risk of stroke events. **c** Risk of coronary heart disease (CHD). 100% fruit juice consumption was modelled with restricted cubic splines in a multivariate random-effects dose–response model (solid line). Dashed lines represent the 95% confidence intervals for the spline model

The analysis of the two studies that investigated the consumption of 100%FJ and total CV events [13, 14] (overall, 65,018 participants and 4087 events, Online Resource-Supplemental Table 4) showed a non-linear association (*p* for non-linearity = 0.02) (Fig. 1a). 100%FJ consumption up to 170 ml per day was associated with significantly lower risk of total CV events, with lowest risk at 78 ml per day (RR = 0.90, 95% CI 0.83–0.97) compared with no 100%FJ consumption (Fig. 1a). There was no significant heterogeneity (*p* = 0.97). On the other hand, a not significant effect of higher levels of reported 100%FJ intake over 170 ml per day was apparent compared with no 100% FJ consumption.

In addition, two studies that did not specify the type of fruit drink consumed analysed the risk of total CV events [15, 33]: in both cases, no significant association was detected between fruit drinks intake and risk of total CV events.

### Risk of stroke

Two studies were available for the analysis of 100%FJ intake and risk of stroke [13, 14] (overall, 65,018 participants and 1283 events, Online Resource-Supplemental Table 4). The analysis of departure from linearity for these two studies indicated a non-linear association between 100%FJ consumption and stroke risk (*p* for non-linearity = 0.01). A level of consumption up to 200 ml per day was significantly associated with lower risk of stroke compared with no consumption, with lowest risk at 100%FJ consumption of 78 ml per day (RR = 0.78, 95% CI 0.66–0.92) (Fig. 1b). No heterogeneity was found (*p* = 0.44). By contrast, there was no significant effect of higher levels of reported 100%FJ intake, above 200 ml per day, compared with no 100%FJ consumption.

Separate analysis of the studies with unspecified fruit drink intake [11, 12] (overall, three cohorts, 118,771 participants and 1039 events) again showed a non-linear association (*p* for non-linearity = 0.01). Fruit drinks intake was significantly and inversely associated with risk of stroke at moderate consumption (Online Resource-Supplemental Fig. 2). The analysis did not detect significant heterogeneity (*p* = 0.88).

### Risk of coronary heart disease

The analysis of the two studies that investigated the association between 100%FJ intake and CHD [13, 14] (overall, 65,018 participants and 2273 events, Online Resource-Supplemental Table 4) indicated no significant association between any consumption of 100%FJ and CHD risk (*p* for non-linearity = 0.15) (Fig. 1c). There was no heterogeneity between studies (*p* = 0.41). A further study evaluated CHD risk for 100%FJ intake, but there were no data available for dose–response analysis [17]. The main findings of this study indicated no significant increase in risk for a consumption of 350 ml/day of 100%FJ consumption.

Separate dose–response analysis of the studies with unspecified fruit drink intake [16, 34] (overall, 3 cohorts, 180,782 participants and 3265 events) showed no significant association between any consumption of fruit drinks and CHD risk (*p* for non-linearity = 0.86) (Online Resource-Supplemental Fig. 3) and no evidence of heterogeneity among studies (*p* = 0.83). Other two studies not specifying the type of fruit drink consumed analysed the risk of CHD [12, 32], with data not available for dose–response analysis. In both studies, there was no association between fruit drink intake and CHD risk.

### Risk of diabetes

**Fig. 2:**
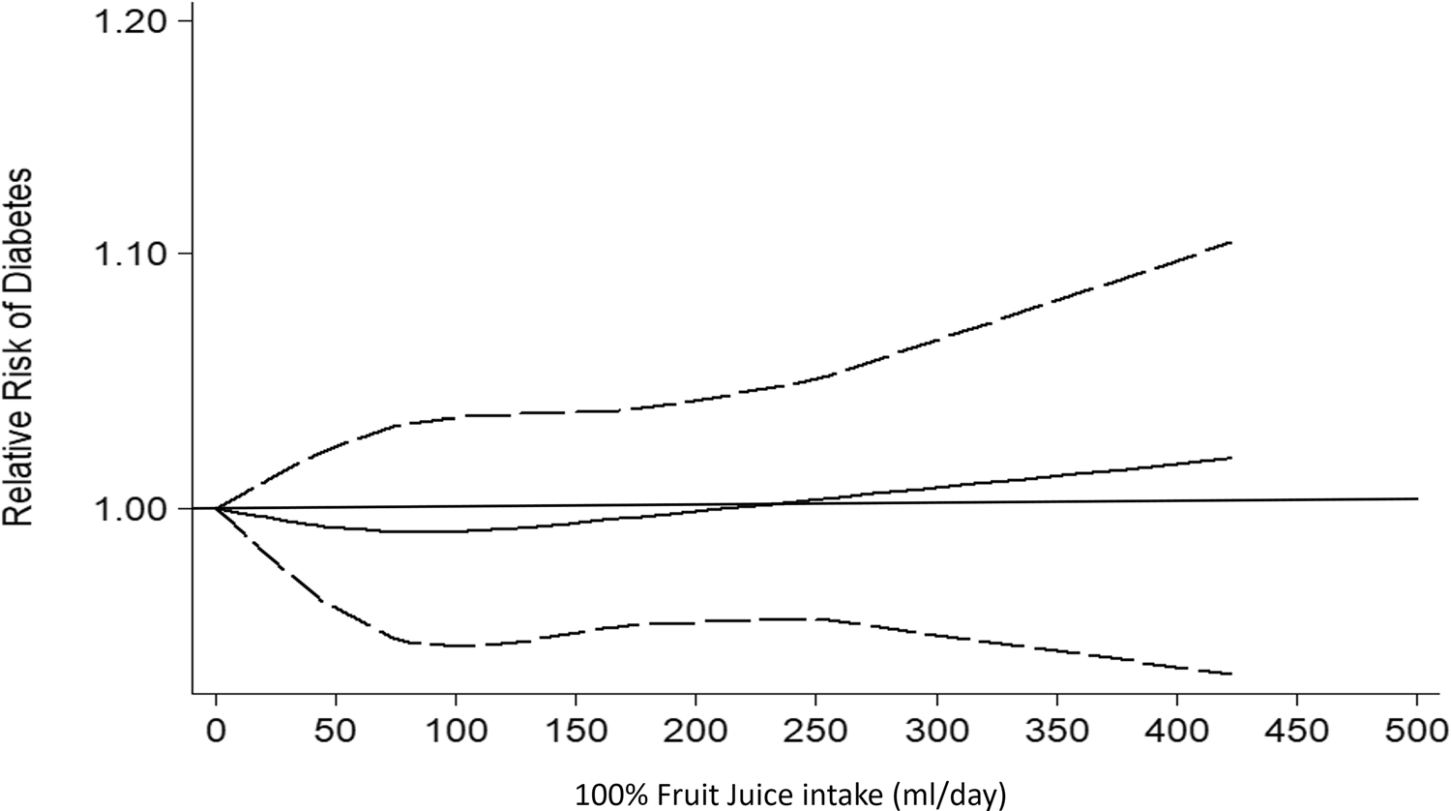
Dose–response association between 100% fruit juice consumption and risk of diabetes. 100% fruit juice consumption was modelled with restricted cubic splines in a multivariate random-effects dose–response model (solid line). Dashed lines represent the 95% confidence intervals for the spline model

The dose–response analysis of the five studies [35, 38, 42, 44, 45] (overall, 286,083 participants and 17,894 new diabetes cases, Online Resource-Supplemental Table 5) that reported 100%FJ intake and risk of diabetes detected no significant association (*p* for non-linearity = 0.83) (Fig. 2), without heterogeneity among studies (*p* = 0.41). Separate analysis with available data on the relationship between citrus 100%FJ intake and diabetes risk [44, 45] showed similar results (*p* for non-linearity = 0.11; heterogeneity, p = 0.40) (Online Resource-Supplemental Fig. 3).

By contrast, additional analysis of the studies with unspecified fruit drink intake [36, 39–41, 43] (overall, five cohorts, 320,014 participants and 31,116 new diabetes cases, Online Resource-Supplemental Table 5) indicated a direct non-linear association between fruit drink consumption and risk of diabetes (*p* for non-linearity < 0.001) (Online Resource-Supplemental Fig. 5). There was significant heterogeneity among studies (*p* = 0.02).

*RCTs* A total of 35 RCTs were included in the different meta-analyses (Online Resource-Supplemental-Table 6). Only one study provided multiple groups including different categories of patients [61]. Six studies assessed the arterial stiffness by pulse wave velocity (PWV) and four the endothelial function using flow-mediated dilation (FMD). The evaluation of the “risk of bias” indicated that the majority of the studies were substantially at moderate-risk (Online Resource-Supplemental Table 3). Quality of body of evidence: the GRADE methodology defines evidence from RCTs as high quality. This score was confirmed for the significant effect on BP and endothelial function and also for no effect on weight and lipids metabolism. For glucose metabolism, the quality was downgraded to moderate because of the large heterogeneity among studies.

The quality of the association between 100%FJ intake and arterial stiffness was downgraded from high to moderate level because of heterogeneity among studies. However, the quality level achieved the high GRADE score when the evaluation was performed on only carotid-femoral PWV results.

### Blood pressure

**Fig. 3:**
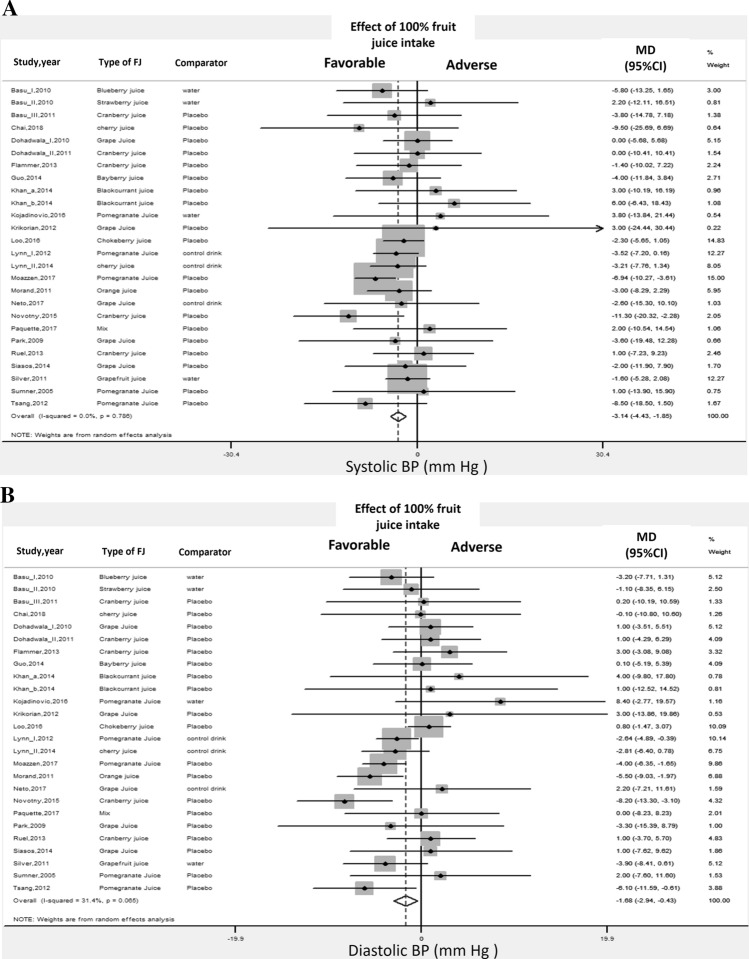
100% fruit juice consumption and blood pressure. **a** Forest plot of the effect of 100% fruit juice intake on systolic blood pressure (BP). **b** Forest plot of the effect of 100% fruit juice intake on diastolic blood pressure (BP). Results are expressed as Mean Difference (MD) and 95% confidence intervals (95% CI). Squares indicate study-specific relative risk estimates (size of the square reflects the study-specific statistical weight); horizontal lines indicate 95% CI; diamond indicates the overall relative risk with its 95% CI

The meta-analysis of the effects of 100%FJ intake on brachial BP included 25 studies [47–49, 52–54, 56, 58, 61–73, 75, 76, 80, 81] (Online Resource-Supplemental Table 6). Pooled analyses showed a significant reduction of both systolic and diastolic BP upon 100%FJ intake in comparison with placebo or control drink (Fig. 3, Table 1). There was low heterogeneity for diastolic BP (Table 1). Visual analysis of the funnel plot indicated little asymmetry (Online Resource-Supplemental Figs. 6–7), whereas Egger’s and Begg’s tests did not find significant evidence of publication bias. Also, the “trim and fill” method did not identify any possibly missing study. Sensitivity analysis showed that the average change in BP did not vary substantially when any individual study or cohort was excluded.

**Table 1:** Meta-analysis of the association between 100% fruit juice intake and cardiovascular risk factors: results of randomised controlled trials

Outcome	*N* of Studies (*N* of cohorts)	*N* of participants	Average intake (ml/day)	Effect estimate Mean difference (95% CI)	*p*-value	Heterogeneity *p*, *I*^2^	Publication bias—Egger’s test (*p*-value; Begg’s test (*p*-value)
Pulse wave velocity (m/s) (53,54,65,66,75,81)	6 (6)	256	435	− 0.03 (− 0.41 to 0.35)	0.58	0.046,56%	0.20; 0.30
Flow-mediated dilation (%) (50,54,61,75)	4 (5)	174	700	2.10% (1.14 to 3.06)	< 0.001	0.5, 0%	0.12; 0.33
Blood pressure (47–49,52–54,56,58,61–73,75,76,80,81)							
Systolic blood pressure (mm Hg)	25 (26)	1032	447	− 3.14 (-4.43 to − 1.85)	< 0.001	0.8, 0%	0.20; 0.40
Diastolic blood pressure (mm Hg)	25 (26)	1032	447	− 1.68 (-2.94 to − 0.43)	< 0.001	0.06, 31%	0.12; 0.22
Body weight (46–48,50,52,53,57–65,71,72,75–80)							
Weight (kg)	19 (20)	805	464	− 0.07 (-0.39 to 0.25)	0.67	1.0, 0%	0.53; 0.40
BMI (kg/m^2^)	12 (13)	527	416	− 0.03 (− 0.15 to 0.10)	0.67	1.0, 0%	0.66; 0.39
Waist circumference (cm)	10 (10)	367	338	0.11 (− 0.94 to 1.16)	0.84	1.0, 0%	0.95; 0.93
Lipid metabolism (46–49,51–62,64,66–68,70–72,75–77,80,81)							
Total cholesterol (mg/dl)	28 (29)	1,180	456	− 3.15 (− 6.43 to 0.13)	0.06	0.99, 0%	0.35; 0.38
LDL-cholesterol (mg/dl)	23 (23)	880	429	0.29 (− 2.62 to 3.20)	0.84	0.97, 0%	0.17; 0.10
HDL-cholesterol (mg/dl)	25 (25)	1,007	414	0.43 (− 0.72 to 1.59)	0.85	0.34, 9%	0.10; 0.30
Triglycerides (mg/dl)	26 (26)	1,049	422	− 0.65 (− 5.83 to 4.52)	0.80	1.0, 0%	0.77; 0.47
Glucose metabolism (47–54,57–59,62–64,67,68,70,74–81)							
Glucose (mg/dl)	23 (23)	845	409	− 1.01 (− 4.02 to 2.00)	0.51	< 0.01, 69%	0.92; 0.85
HOMA index (U)	11 (11)	487	415	0.01 (− 0.28 to 0.30)	0.50	0.66, 0%	0.80; 0.70
Insulin (%)	11 (11)	424	430	3.4 (− 7.2 to 14.0)	0.53	0.88, 0%	0.80; 0.70
Glycated haemoglobin (%)	3 (3)	120	323	− 0.10 (− 0.31 to 0.10)	0.32	0.98, 0%	–

*Additional analyses* Only the study design was a significant source of heterogeneity for the effect on diastolic BP. Of note, sub-group analysis stratified by type of 100%FJ consumed found that pomegranate juice significantly improved BP (Online Resource-Supplemental Table 8).

### Body weight

**Fig. 4:**
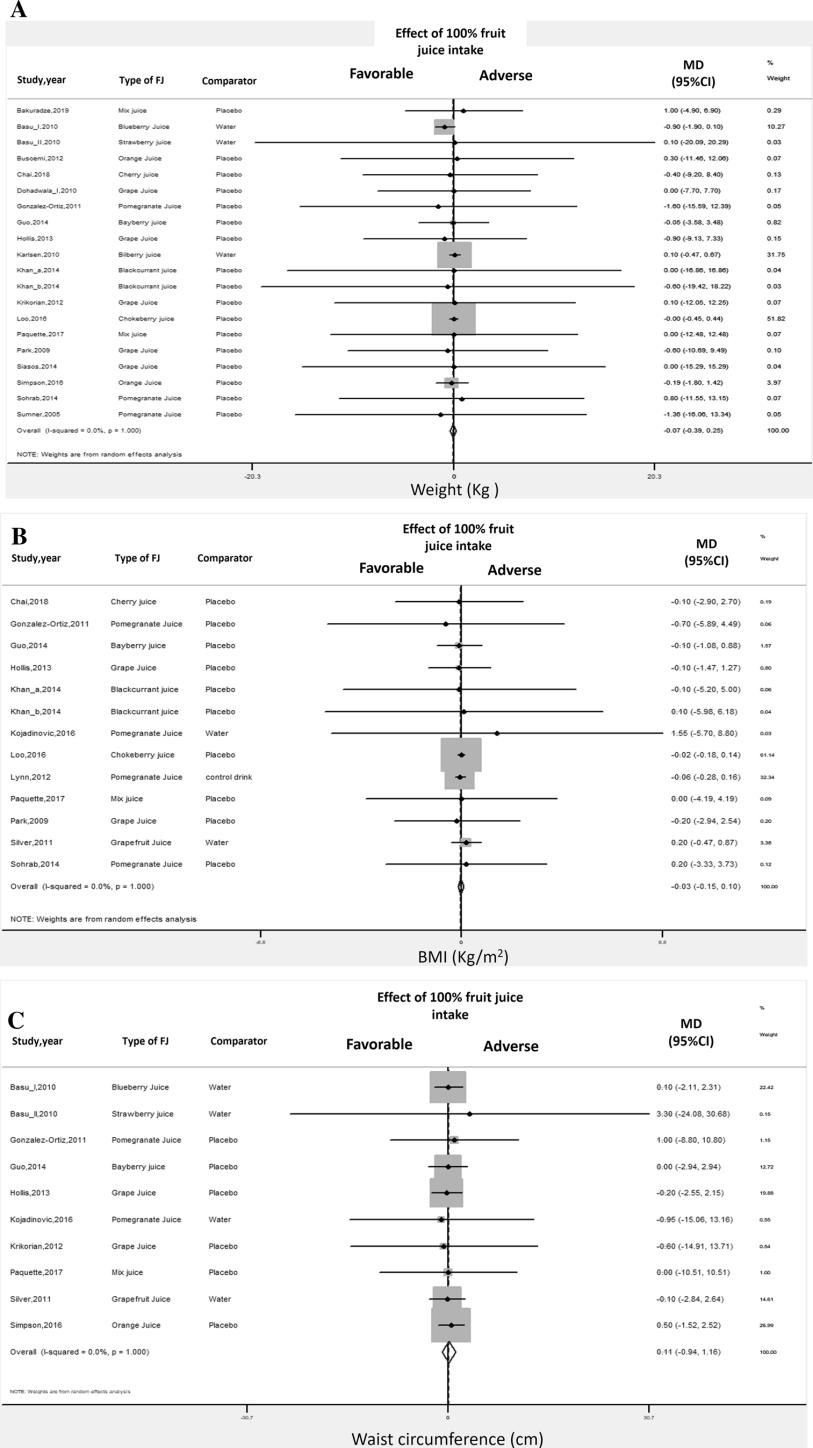
100% fruit juice consumption and body weight. **a** Forest plot of the effect of 100% fruit juice intake on weight. **b** Forest plot of the effect of 100% fruit juice intake on BMI. **c** Forest plot of the effect of 100% fruit juice intake on waist circumference. Results are expressed as Mean Difference (MD) and 95% confidence intervals (95% CI). Squares indicate study-specific relative risk estimates (size of the square reflects the study-specific statistical weight); horizontal lines indicate 95% CI; diamond indicates the overall relative risk with its 95% CI

Pooled analysis of the effect of 100%FJ intake on body weight changes showed no significant association for all the specific outcomes considered: weight, BMI, and waist circumference [46–48, 50, 52, 53, 57–65, 71, 72, 75–80] (Table 1, Online Resource-Supplemental Table 6, Fig. 4). For all the outcomes, there was no significant heterogeneity among studies and no evidence of publication bias (Table 1, Online Resource-Supplemental Figs. 8–10). Sensitivity analysis showed that the average change in different body weight expressions did not vary substantially with the exclusion of any individual study or cohort.

*Additional analyses* No study characteristics affected the relationship between 100%FJ intake and body weight changes by meta-regression and subgroup analysis (Online Resource-Supplemental Table 9).

### Lipid profile

The analyses of the effect of 100%FJ intake on the lipid metabolic profile did not detect any significant association for total cholesterol, LDL-cholesterol, HDL-cholesterol, and triglycerides [46–49, 51–62, 64, 66–68, 70–72, 75–77, 80, 81] (Table 1, Online Resource-Supplemental Table 6, Online Resource-Supplemental Figs. 11–14). There was no significant heterogeneity among studies (Table 1), and no evidence of publication bias (Online Resource-Supplemental Figs. 15–18). The evaluation of single study effect did not show significant associations for any of the included studies. However, only for total cholesterol, sensitivity analysis indicated a significant inverse association between cholesterol changes and 100%FJ intake after exclusion of a few single studies.

*Additional analyses* Age and features of the participants, design of the studies, type of comparator used, characteristics of 100%FJ, and diet during the studies were significant sources of heterogeneity on changes of HDL-cholesterol (Online Resource-Supplemental Table 10).

### Glucose metabolism

Pooled analysis of the effect of 100%FJ intake on glucose metabolism showed no significant association for all the outcomes considered: serum glucose, HOMA index, serum insulin, and glycated haemoglobin [47–54, 57–59, 62–64, 67, 68, 70, 74–81] (Table 1, Online Resource-Supplemental Table 6, Online Resource-Supplemental Figs. 19–22). Significant heterogeneity was observed only for serum glucose (Table 1). Visual analysis of the funnel plot indicated little asymmetry only for changes in glucose levels (Online Resource-Supplemental Figs. 23–25), but the formal tests did not find significant evidence of publication bias (Table 1). Sensitivity analysis showed that the average change in the different outcome levels did not vary substantially with the exclusion of any individual study.

*Additional analyses* Meta-regression and subgroup analyses found gender, country of origin, characteristics of 100%FJ and study design as significant sources of heterogeneity about the effect of 100%FJ intake on serum glucose (Online Resource-Supplemental Table 11). Analysis of the features that may affect changes in HOMA index detected only the type and the energy content of the comparator as significant sources of heterogeneity (Online Resource-Supplemental Table 11).

### Arterial stiffness

In the pooled analysis of six RCTs [53, 54, 65, 66, 75, 81] (Online Resource-Supplemental Table 6), 100%FJ intake was not associated with changes in PWV (Table 1, Online Resource-Supplemental Fig. 26). There was significant heterogeneity among studies (Table 1). Visual analysis of the funnel plot indicated little asymmetry (Online Resource-Supplemental Fig. 27), whereas formal tests did not find significant evidence of publication bias (Table 1). Of note, changes in BP did not affect changes in PWV.

Separate analysis including only the studies using the carotid-femoral PWV changes (i.e., the gold standard for measuring large artery stiffness) [53, 54, 75, 81] (four studies, 162 total participants) indicated a significant beneficial effect of 100%FJ intake compared with the comparator drink (MD: − 0.38 m/s; *p* = 0.04). In addition, the analysis did not detect heterogeneity among studies (Online Resource-Supplemental Fig. 26).

### Flow-mediated dilation

Pooled analysis of four RCTs [50, 54, 61, 75] (Online Resource-Supplemental Table 6) showed that 100%FJ intake was significantly associated with increased FMD compared with the comparator drink (Table 1, Online Resource-Supplemental Fig. 28). There was no significant heterogeneity among studies and no evidence of publication bias (Table 1, Online Resource-Supplemental Fig. 29). Sensitivity analysis showed that the average change in FMD did not vary substantially when any individual cohort was excluded. Even in this case, changes in BP did not affect changes in FMD.

## Discussion

### Main study results

Our meta-analysis indicated that 100% FJ consumption is not associated with increased CV risk. Prospective data support a non-linear dose–response association between 100% FJ consumption and the rate of incident stroke and total CV disease, with evidence of a statistically significant beneficial effect at low-moderate intake (~ 80 ml/day). There was no significant association between 100% FJ consumption and risk of CHD or diabetes. The results of the RCTs support a beneficial effect on CV risk due to the favourable impact of 100% FJ intake on BP levels, arterial compliance and endothelial function. A neutral effect was detected on body weight, lipid profile and glucose metabolism. The results were supported by the GRADE categorization that detected high quality of the results for RCTs and moderate quality for prospective studies.

The results on CV risk were strengthened by no evidence of heterogeneity among studies. While, in general, our results are consistent with the trend found by a previous systematic review [82], relevant elements of novel information of the present meta-analysis include separate assessment of the studies with specified 100% FJ intake, detailed dose–response analysis with detection in some cases of non-linear relationships, and assessment of the quality of the results by GRADE categorization.

Our results are also substantially in agreement with a recent study of a European population [14, 83] showing a non-linear relationship between 100%FJ consumption and CV risk with significant benefit at low-moderate consumption.

By contrast, they are at variance with another recent study on a large American population, in which a very high 100% FJ intake was associated with significantly higher CHD death rate [17]. The latter study had major limitations in the small number of CHD-related deaths, in the very large amount of 100% FJ intake considered for the assessment of risk, in the probable misclassification of 100%FJ intake and in the lack of assessment of a potential dose–response relationship.

### 100% FJ intake, BP and arterial function

The beneficial effect of moderate 100%FJ intake on CV risk, in particular on the risk of stroke, is supported by the results on BP. Pooled analysis of a large number of studies showed that 100% FJ intake was associated with a reduction of more than 3 mmHg for systolic and approximately 2 mmHg for diastolic BP. Compared with previous meta-analyses of the effects of 100%FJ intake on BP, our study used more stringent inclusion criteria and adopted a robust methodology for the assessment of potential sources of heterogeneity, potential publication bias, robustness and quality of results. Furthermore, we explored for the first time the effect of 100% FJ intake on arterial stiffness, an expression of CV organ damage [1]. In this regard, the analysis of the RCTs that used carotid-femoral PWV as a proxy for arterial stiffness did show a significant and favourable effect of 100% FJ. In line with this result are the findings on endothelial function: pooled analysis of FMD, another expression of early CV damage [84], found a significant improvement upon 100% FJ intake, with no significant heterogeneity among studies, no effect dependent on BP changes, and a high quality level as suggested by GRADE score.

Unfortunately, only two studies explored the risk to develop hypertension as a function of the consumption of fruit drinks [35, 37] (Online Resource-Supplemental Table 5). One American study, including young participants, suggested a significant and inverse trend between fruit drinks intake and risk of hypertension [37]. The other American study, including a large post-menopausal female sample, found a not significant association between 100% FJ consumption and development of hypertension at different levels of intake [35].

### Possible mechanisms involved

Although our study has not the potential to shed light on pathophysiological mechanisms, it is conceivable that the relatively high amount of minerals, vitamins and bioactive compounds associated with 100% FJ consumption explain the benefit on CV risk [5, 85] and, in particular, on arterial stiffness, endothelial function, BP and risk of stroke.

In addition to the clinical and epidemiological evidence [85–87], many studies in animal models indicated that a high potassium intake may reduce CV organ damage, even independently from its effect on BP [88, 89]. A meta-analysis of RCTs suggested a beneficial effect of potassium intake on arterial stiffness [90].

Among the bioactive substances highly bioavailable in 100% FJ, polyphenols (e.g., hesperidin, naringenin) may contribute to the cardiovascular benefit, in particular by modulation of the nitric oxide (NO)–cGMP pathway. These compounds may regulate the levels and the activity of endothelial NO synthase and therefore NO bioavailability [68, 91, 92], also involving intracellular Ca^2+^ [93]. Polyphenols also cause inhibition of platelet aggregation, enhance release of platelet-derived NO, and reduce superoxide production [91, 94], oxidative DNA damage [55] and lipid peroxidation [95–97]. In addition, experimental studies suggested that hesperidin, the major polyphenol contained in orange juice, exerts its anti-inflammatory and antioxidant effects also by suppression of gene expression of some proinflammatory cytokines (e.g., TNF-a, IL-1β, IL-6) [98, 99], reduction of the expression of the intercellular adhesion molecule-1 (ICAM-1) [100], and production of metalloproteinases [94].

Also the antioxidant activity of vitamins may support the beneficial CV effect of 100% FJ intake. Vitamin A and derivatives were reported to reduce coronary heart disease risk [101] and this was attributed to modulation of endothelial cells differentiation and increase in NO synthesis [102–104]. A high content of Vitamin C may contribute to endothelium-dependent vasodilation by increasing NO availability [105], in particular by modulation of endothelial NO synthase [106]. Vitamin E decreases cell adhesion molecule expression [107] and monocyte adhesion to the endothelium [108]. Finally, folic acid content may exert beneficial effects on CV risk, by reducing homocysteine production and its detrimental effects [109, 110].

Based on the available evidence and on the results of our analyses, a biphasic dose–response relationships apparent, where the beneficial effect detected at low-moderate 100% FJ consumption may be explained by the favourable health effect of “low dose” bioactive substances [111]. On the contrary, at a high level of consumption, the benefit may be offset by the unfavourable consequences of excess sugar and calorie intake [111].

Although there is an almost complete lack of studies comparing the effects on health outcomes of low-moderate consumption of 100%FJ with those of equivalent amounts of fresh fruit [76], it is indeed conceivable that the latter would be at least as beneficial as 100% FJ because of the higher content of fibre and in many cases of the other beneficial compounds. Actually, the benefit associated with 100% FJ intake in observational studies may result from partial compensation of a generally inadequate fresh fruit consumption at the population level.

### 100% FJ intake, adiposity and risk of diabetes

100%FJ intake in our study appeared to have a neutral effect on adiposity in all its expressions. Noteworthy, none of the included RCTs show significant changes in body weight upon 100%FJ consumption. These results are in line with the recent prospective study by Garduño-Alanís et al. in 5000 Russian individuals indicating no significant BMI increase after 3 years of follow-up in 100%FJ consumers [112]. Likewise, in a study on a large sample of post-menopausal women, 100%FJ consumption was associated with only a small not clinically relevant increase in weigh after 3 years of follow-up [113]. A similar not clinically relevant increase in BMI was also found in children and adolescents by a recent meta-analysis of prospective studies [114].

Other experimental investigations, in particular those on citrus 100%FJ, did not indicate a detrimental effect on body weight and actually suggested that citrus extracts may induce lipolysis [115, 116] and reduce fat accumulation [117], also by modulation of gut microbiota [118].

Our systematic review did not detect a significant association between 100% FJ consumption and risk of diabetes and in this respect our results are in general consistent with those of previous meta-analyses [119, 120]. One of these indeed showed that the consumption of unspecified fruit drinks was associated with an increased risk of diabetes whereas that of 100% FJ was not [119]. In another recent meta-analysis by Imamura et al. [120] the consumption of sugary drinks and 100% FJ in excess of 250 ml per day was also associated with a significant increase in the risk of diabetes. Our dose–response analysis detected a non-linear significant direct association between fruit drinks consumption and risk of diabetes but no association for 100% FJ or citrus 100%FJ intake. At variance with previously reported experimental data [3, 4], our pooled analysis of RCTs indicated no significant association between 100% FJ intake and several parameters of glucose metabolism. In particular, 100% FJ intake did not affect serum levels of glucose and insulin nor the HOMA index. In addition, from the analysis of three available studies, there was no significant association with glycated-haemoglobin levels, further supporting a long-term neutral effect. These results confirm those found in previous meta-analyses [21, 22].

### 100% FJ intake and lipid profile

The exploration of the lipid profile showed no significant effect of 100% FJ intake in our meta-analysis. Previous systematic reviews as well as some experimental study reported a beneficial effect on lipid profile with consumption of 100% FJ [121–123].

## Strengths and limitations

This study has several strengths: (a) the evaluation of both prospective and intervention studies; (b) the stringent inclusion criteria; (c) a large number of participants for prospective evaluation and a large number of studies for intervention assessment; (d) the robustness of the findings by sensitivity and sub-group analysis; (e) the comprehensive exploration of possible sources of heterogeneity; (f) the substantially low heterogeneity among studies and no evidence of publication bias; (g) the evaluation of shape and strength of the dose–response relationship; (h) the assessment of overall quality of evidence using the GRADE assessment approach.

On the other hand, the study also has limitations. For prospective studies, their observational nature impairs conclusions about possible cause-effect relationships.

Also the questionnaire to assess the extent of 100%FJ consumption is subject to limitations in prospective studies both in the administered and the self-administered version, because of potential misclassification of quantity and quality of 100%FJ consumption.

The heterogeneity in the characteristics of the included studies is an important limitation, in particular for the RCTs, which varied for age, health status, type of 100%FJ utilized, serving size and comparator, length of intervention. This limitation was explored by sub-group and meta-regression analysis, which in general found little evidence of subgroup differences. However, in some subgroup analyses, the test was performed including relatively few studies in one of the subgroups, hence in those cases definitive conclusions cannot be reached. In the analysis of prospective studies, an important source of heterogeneity may be the different serving size due to geographical location, but this limitation was dealt with by dose–response analysis. Furthermore, although all the included prospective studies adjusted for several relevant confounding factors (including age, body mass index, and other CV risk factors), residual confounding by other potential factors cannot be ruled out.

This meta-analysis was not able to detect consistent effects of individual types of 100% FJ, apart for the effect of pomegranate juice on BP, because of the low number of studies in each subgroup. Of note, a focused evaluation of the citrus/orange 100% FJ effect, the most popular juice worldwide, suggests substantial similar results of this type of juice as compared to the unspecified 100%FJ both for CV outcomes [13, 14] and diabetes risk [35, 44, 45]. There was also heterogeneity with regard to the fruit juices or fruit juice preparations used in the RCTs: nevertheless, the effects observed in the studies that reported on reconstituted 100%FJ or fruit juices as such were not significantly different.

Also, because few studies evaluated the effect of 100%FJ on CV organ damage, only the effect on arterial stiffness was explored by this meta-analysis. About CV organ damage, our systematic search detected only two studies that examined the effect of 100% FJ on other outcomes (i.e., carotid intima-media thickness) [124, 125]. One RCT, including approximately 300 participants at moderate CHD risk and with a relatively high intima-media thickness, found a not significant reduction in these values after 1 year of 240 ml of pomegranate juice supplementation [125]. The other one, non RCT, found progressively lower values in ten participants, during 1 year of 50 ml of pomegranate juice supplementation [124].

Finally, further limitations are given by the relatively small number of prospective studies and cohorts available, by the residual possibility of publication bias and by the difficulty to draw definitive conclusions with regard to the interaction with age, gender and race given the peculiar composition of the study cohorts available. This notwithstanding, the observational design and the process of meta-analysis, with the calculation of a pooled estimate of the effect and the dose–response analysis in a large number of participants, are functional to overcome at least in part this problem.

## Conclusions

The results of our study show that low-moderate 100% FJ consumption is not associated with increased risk of CV disease and that actually it is associated with apparent benefit against the development of CV events through a non-linear relationship. The positive changes in BP, arterial compliance and endothelial function may help explain the effect of 100% FJ intake on risk of CV disease, in particular on stroke risk. These findings are in keeping with the inverse association also occurring between regular fresh fruit consumption and risk of CV disease.

Given the importance of CV diseases [9, 10, 126, 127] and the popularity of fruit drinks consumption around the world [7, 8], the relationship between 100% FJ and CV risk assumes considerable relevance. Therefore, to clearly classify 100% FJ from unspecified fruit drink is crucial both for future research and for correct consumption. Further properly powered RCTs of the effect of long-term moderate 100% FJ consumption are warranted to determine possible cause-effect relationships, to disentangle the effects of different types of 100% FJ (e.g., orange juice), and to overcome the currently limited evidence with respect to the interactions with age, ethnicity and diseases. In particular, intervention studies with carefully controlled intake of 100% FJ should evaluate the mechanisms of its effects on BP and CV organ damage to extend current knowledge in this field.

### Implications for public health

Based on recent data, the overall average consumption of 100% FJ is 38 ml per day [7], with women’s consumption being higher than that of men. Recent European estimates found an average consumption of 49 ml per day, with highest consumption in Western (52 ml) and lowest in Eastern countries (29 ml) [8].

International dietary guidelines are not in agreement on recommendations about 100%FJ consumption. In the United States, the Guidelines for Healthy Eating recommend the daily consumption of two cups-equivalent of fruit (one cup = 237 ml), of which less than half can be taken as 100% FJ [128]. For the American Heart Association, one of the recommended portions of fruit can be replaced by the consumption of 100%FJ (½ cup = 115 ml) [129]. In the United Kingdom, the dietary guidelines suggest that 100% FJ can contribute one portion of the daily fruit portion size (150 ml) [130]. Conversely, Dutch and Italian guidelines suggest more caution on 100% FJ consumption. Both guidelines do not recommend the consumption of 100%FJ in place of fresh fruit, because one classified 100%FJ in the category of sugar-containing beverages [131], and the Italian guidelines consider it as “unnecessary” consumption [132].

While fresh fruit in the proper amounts cannot be surrogated by processed fruit consumption, the results of our study indicate no harm for the adult population from low-moderate 100% FJ consumption and suggest that low-moderate consumption of 100% FJ in the context of a balanced diet and as an alternative to sugar-sweetened beverages needs not to be discouraged. Although these results also show that high levels of 100% FJ intake were not associated with a detrimental CV effect, it would be hazardous to try to reach any conclusion on “safe levels” of intake, given the few prospective data available and the high heterogeneity of the level of consumption between trials and prospective studies. Therefore, only general consideration can be made. Indeed, it seems reasonable to suggest particular caution by overweight and obese individuals and by anyone else at higher risk of diabetes mellitus. Furthermore, more research is warranted to compare the effects on health outcomes of low-moderate consumption of 100% FJ with that of equivalent amounts of fresh fruit, given the almost complete lack of sound information available in this respect [75, 133].

## Electronic supplementary material

Below is the link to the electronic supplementary material.

Supplementary file1 (PDF 279 kb)

Supplementary file2 (PDF 705 kb)
