# Supplementary material for: 100% Fruit juice intake and cardiovascular risk: a systematic review and meta-analysis of prospective and randomised controlled studies
Source: Eur J Nutr. 2020 Nov 4;60(5):2449–67. doi: 10.1007/s00394-020-02426-7 (PMC8275541; doi:10.1007/s00394-020-02426-7)
Supplement: Supplementary file 1 — Supplementary file1 (PDF 279 kb) [file 394_2020_2426_MOESM1_ESM.pdf]

Supplemental Table 1. The PRISMA Checklist

Section/topic	#	Checklist item	Reported on page #
**TITLE**			
Title	1	Identify the report as a systematic review, meta-analysis, or both.	1
**ABSTRACT**			
Structured summary	2	Provide a structured summary including, as applicable: background; objectives; data sources; study eligibility criteria, participants, and interventions; study appraisal and synthesis methods; results; limitations; conclusions and implications of key findings; systematic review registration number.	2
**INTRODUCTION**			
Rationale	3	Describe the rationale for the review in the context of what is already known.	4
Objectives	4	Provide an explicit statement of questions being addressed with reference to participants, interventions, comparisons, outcomes, and study design (PICOS).	4
**METHODS**			
Protocol and registration	5	Indicate if a review protocol exists, if and where it can be accessed (e.g., Web address), and, if available, provide registration information including registration number.	5
Eligibility criteria	6	Specify study characteristics (e.g., PICOS, length of follow-up) and report characteristics (e.g., years considered, language, publication status) used as criteria for eligibility, giving rationale.	5-6, Supplemental Tables
Information sources	7	Describe all information sources (e.g., databases with dates of coverage, contact with study authors to identify additional studies) in the search and date last searched.	5, Supplemental Tables
Search	8	Present full electronic search strategy for at least one database, including any limits used, such that it could be repeated.	5, Supplemental Tables, Supplemental Figures
Study selection	9	State the process for selecting studies (i.e., screening, eligibility, included in systematic review, and, if applicable, included in the meta-analysis).	5-7 Supplemental Tables, Supplemental

			Figures
Data collection process	10	Describe method of data extraction from reports (e.g., piloted forms, independently, in duplicate) and any processes for obtaining and confirming data from investigators.	5-6<br>Supplemental Tables, Supplemental Figures
Data items	11	List and define all variables for which data were sought (e.g., PICOS, funding sources) and any assumptions and simplifications made.	5-7<br>Supplemental Tables, Supplemental Figures
Risk of bias in individual studies	12	Describe methods used for assessing risk of bias of individual studies (including specification of whether this was done at the study or outcome level), and how this information is to be used in any data synthesis.	6
Summary measures	13	State the principal summary measures (e.g., risk ratio, difference in means).	7,8
Synthesis of results	14	Describe the methods of handling data and combining results of studies, if done, including measures of consistency (e.g., I^2) for each meta-analysis.	7,8
Risk of bias across studies	15	Specify any assessment of risk of bias that may affect the cumulative evidence (e.g., publication bias, selective reporting within studies).	7,8
Additional analyses	16	Describe methods of additional analyses (e.g., sensitivity or subgroup analyses, meta-regression), if done, indicating which were pre-specified.	7,8
**RESULTS**			
Study selection	17	Give numbers of studies screened, assessed for eligibility, and included in the review, with reasons for exclusions at each stage, ideally with a flow diagram.	8,9,11,12<br>Supplemental Tables, Supplemental Figures
Study characteristics	18	For each study, present characteristics for which data were extracted (e.g., study size, PICOS, follow-up period) and provide the citations.	8,9,11,12<br>Supplemental Tables,
Risk of bias within studies	19	Present data on risk of bias of each study and, if available, any outcome level assessment (see item 12).	8,9,11,12<br>Supplementa

			l Tables
Results of individual studies	20	For all outcomes considered (benefits or harms), present, for each study: (a) simple summary data for each intervention group (b) effect estimates and confidence intervals, ideally with a forest plot.	9-14, Supplemental Tables, Supplemental Figures
Synthesis of results	21	Present results of each meta-analysis done, including confidence intervals and measures of consistency.	9-14 Supplemental Tables, Supplemental Figures
Risk of bias across studies	22	Present results of any assessment of risk of bias across studies (see Item 15).	9-14 Supplemental Tables, Supplemental Figures
Additional analysis	23	Give results of additional analyses, if done (e.g., sensitivity or subgroup analyses, meta-regression [see Item 16]).	9-14 Supplemental Tables, Supplemental Figures
**DISCUSSION**			
Summary of evidence	24	Summarize the main findings including the strength of evidence for each main outcome; consider their relevance to key groups (e.g., healthcare providers, users, and policy makers).	14-18
Limitations	25	Discuss limitations at study and outcome level (e.g., risk of bias), and at review-level (e.g., incomplete retrieval of identified research, reporting bias).	18-19
Conclusions	26	Provide a general interpretation of the results in the context of other evidence, and implications for future research.	19-21
**FUNDING**			
Funding	27	Describe sources of funding for the systematic review and other support (e.g., supply of data); role of funders for the systematic review.	3

Supplemental Table 2. Newcastle-Ottawa Quality Assessment Scale for cohort studies included in the meta-analysis

META-ANALYSES	SELECTION				COMPARABILITY	OUTCOME			TOTAL SCORE
	Representativeness of the exposed cohort	Selection of the non-exposed cohort	Ascertainment of exposure	Demonstration that outcome of interest was not present at start of study		Assessment of outcome	Was follow-up long enough for outcomes to occur	Adequacy of follow up of cohorts	
**Cardiovascular risk**									
Collin et al., 2019	*	*	*	*	**	*	*	*	**9**
Hansen et al., 2010	*	*	*	*	**	*	*	*	**9**
Hung et al., 2004		*	*	*	**	*	*		**7**
Joshipura et al., 1999		*	*	*	**	*	*	*	**8**
Joshipura et al., 2001		*	*	*	**	*	*	*	**8**
Joshipura et al., 2008		*	*	*	**	*	*		**7**
Lai et al., 2015		*	*	*	**	*	*		**7**
Mink et al., 2007		*	*	*	**	*	*	*	**8**
Scheffers et al., 2018	*	*	*	*	**	*	*		**8**
Simila et al., 2013		*	*	*	**	*	*		**7**
**Diabetes**									
Auerbach et al., 2017		*	*	*	**	*	*		**7**
Bazzano et al., 2008		*	*	*	**		*		**6**
Fagherazzi et al., 2013		*	*	*	**	*	*	*	**8**
Interact et al., 2013	*	*	*	*	**	*	*	*	**9**
Muraki et al., 2013		*	*	*	**	*	*	*	**8**
Mursu et al., 2014		*	*	*	**	*	*	*	**8**
O'Connor et al., 2015	*	*	*	*	**	*	*		**8**
Odegaard et al., 2010	*	*	*	*	**	*	*	*	**9**
Palmer et al., 2008		*	*	*	**		*	*	**7**
Scheffers et al., 2020	*	*	*	*	**	*	*		**8**
**Hypertension**									
Auerbach et al., 2017		*	*	*	**	*	*		**7**
Cardia et al., 2010	*	*	*		**	*	*		**7**

Supplemental Table 3. Risk of bias of the randomised controlled trials included in the meta-analysis

Studies with intention-to-treat	Study ID	Randomization process	Deviations from intended interve	Missing outcome data	Measurement of the outcome	Selection of the reported result	Overall
	Bakurazde 2019	?	?	−	+	?	−
	Basu (I) 2010	?	−	−	+	−	−
	Basu (II) 2010	?	?	+	+	−	−
	Basu (III) 2011	?	?	+	+	−	−
	Buscemi 2012	?	+	+	+	+	!
	Cerdà 2006	?	+	+	+	?	!
	Chai 2018	+	+	+	+	?	!
	Dohadwala (I) 2010	+	?	+	+	+	!
	Dohadwala (II) 2011	+	?	+	+	+	!
	Duthie 2006	?	?	+	+	−	−
	Flammer 2013	?	?	−	+	?	−
	Gonzalez-Ortiz, 2011	?	+	+	+	?	!
	Guo 2014	+	+	+	+	?	!
	Hollis 2009	?	+	+	+	?	!
	Karlsen 2010	+	+	+	+	?	!
	Khan 2014	+	+	+	+	?	!
	Kojadinovic 2016	−	+	+	+	?	−
	Krikorian 2012	?	+	+	+	?	!
	Loo 2016	?	+	+	+	?	!
	Lynn (I) 2012	+	?	+	+	?	!
	Lynn (II) 2014	+	+	+	+	?	!
	Moazzen 2017	?	+	+	+	?	!
	Morand 2011	?	+	+	+	+	!
	Neto 2017	?	+	+	+	?	!
	Novotny 2015	+	+	+	+	+	+
	Paquette 2017	+	?	+	+	+	!
	Park 2009	?	+	+	+	?	!
	Ruel 2013	?	+	+	+	?	!
	Shidfar 2012	?	+	+	+	+	!
	Siasos 2011	?	+	+	+	?	!
	Silver 2011	?	?	+	+	+	!
	Simpson 2016	+	+	+	+	+	+
	Sohrab (I) 2014	+	−	−	+	+	−
	Sohrab (II) 2015	+	+	+	+	+	+
	Sumner 2005	+	+	+	+	?	!
	Tsang 2012	?	−	−	+	?	−

+ Low risk
? Some concerns
− High risk

Supplemental Table 4. Characteristics of the prospective studies included in the meta-analysis of the risk of cardiovascular events

First author, year	Country	Population	Participants (n)/ Events (n)	Follow-up (years)	Type of Juice	100% Fruit Juice	Age (yrs)	BMI (kg/m^2)	Dietary assessment tool	Outcome (type of assessment)
Collin, 2019 (17)	USA	REGARDS (59% men, 41%women)	13,440 / CHD: 168	6	All types	Specified	63.6	-	FFQ	CHD mortality (medical records and death certificates)
Hansen, 2010 (16)	Europe (Denmark)	Danish Diet, Cancer and Health cohort study (47% men, 53% women)	54,383 / CHD: 1,075	7.7	Grape and orange juice	Unspecified	55	26	FFQ	Acute coronary syndrome (National registry and Death registry)
Hung, 2004 (33)	USA	NHS (women) & HPFS (men)	NHS 71,910 / Total CV: 1,964 & HPFS 37,725 / Total CV: 1,670	NHS 14<br>HPFS 12	Citrus juice	Unspecified	-	-	FFQ	CV events [myocardial infarction & stroke] (medical records)
Joshipura, 1999 (11)	USA	NHS (women)	75,596/ Stroke: 366	14	Citrus juice	Unspecified	46.2	24.3	FFQ	Stroke events (confirmed by medical records)
	USA	HPFS (men)	38,683 / Stroke: 204	8			53.7	25.4	FFQ	
Joshpura, 2001 (34)	USA	NHS (women)	84,251 / CHD: 1127	14	Citrus juice	Unspecified	-	-	FFQ	CHD (medical records reviewed by physicians)
	USA	HPFS (men)	42,148 / CHD: 1063	8					FFQ	
Joshpura, 2009 (15)	USA	NHS (women) & HPFS (men): *Low (<40%); Moderate (40-55%)	NHS 70,870 / Total CV: 1852 & HPFS 38,918 / Total CV: 2040	16	Citrus juice	Unspecified	61.2	26.0	FFQ	Total CV events [CHD & stroke events] (medical records)

		High (>55%)		14			59.7	26.2	FFQ	
Lai, 2015 (13)	Europe (UK)	Women's cohort Study (women)	30,458 / Total CV: 286 Stroke: 148 CHD: 138	16.7	All types	Specified	51.6	24.3	FFQ	CV mortality (UK's NHS Central Register)
Mink, 2007 (12)	USA	Iowa Women's Health Study (Post-menopausal women)	34,492 / Stroke: 469 CHD: 1,329	16	Citrus juice, other fruit juice	Unspecified	61.5	26.9	FFQ	Total CV mortality (State Health Registry)
Scheffers, 2018 (14)	Europe (Netherlands)	MORGEN & Prospect cohort (26% men, 74% women)	34,560 / Total CV: 3,801 Stroke: 1,135 CHD: 2,135	14.6	All types	Specified	48.8	25.5	FFQ	Total CV events, [& Stroke events & CHD events] (Hospital Register and Statistics Netherlands)
Simila, 2013 (32)	Europe (Finland)	Alpha-Tocopherol, Beta-carotene Cancer Prevention Study (men)	21,995 / CHD: 4,379	19	All types	Unspecified	56.6	25.8	FFQ	CHD events (National registry and Death registry)

CHD: coronary heart disease, CV: cardiovascular, FFQ: food-frequency questionnaire, HPFS: Health Professionals' Follow-Up Study, MORGEN: Monitoring Project on Chronic Disease Risk Factors, NHS: Nurses' Health Study, REGARDS: The Reasons for Geographic and Racial Differences in Stroke study; * Risk stratified by percentage of energy from carbohydrate intake

Supplemental Table 5. Characteristics of the prospective studies included in the meta-analysis of the risk of diabetes and hypertension

First author, year (ref)	Country	Population	Participants (n)/ Cases (n)	Follow-up (year)	Type of Juice	100% Fruit Juice	Age (year)	BMI (kg/m^2)	Dietary assessment tool	Outcome (type of assessment)
Auerbach, 2017 (35)	USA	The Women's Health Initiative (post-menopausal women)	114,219 / 11,488 [HPT: 80,539 / 36,314]	7.8	All types	Specified	64.5	-	FFQ	Self-reported diagnosis (diabetes & hypertension)
Bazzano, 2008 (36)	USA	The Nurses' Health Study (women)	71,346 / 4,529	18	All types	Unspecified	50.1	23.6	FFQ	Self-reported diagnosis (diabetes)
Duffey, 2010 (37)	USA	CARDIA (46% men, 54% women)	2639 / HPT: 609	20	All types	Unspecified	25.5	24.5	Questionnaire	Diagnosis by physicians (hypertension)
Fagherazzi, 2013 (38)	Europe (France)	E3N (women)	66,118 / 1,369	14	All types	Specified	52.8	22.8	Questionnaire	Self-reported diagnosis validated by physicians (diabetes)
Interact consortium, 2013 (39)	Europe (multicentric)	EPIC-Interact study (38% men, 62% women)	27,058 / 11,684	11.7	All types	Unspecified	52.4	26	FFQ	Self-report, linkage to primary-care registers, secondary-care registers, medication use (drug registers), hospital admissions and mortality data (diabetes).
Muraki, 2013 (40)	USA	NHS, NHS II, HPFS	187,382 / 12,198	19.6	All types	Unspecified	45.7	-	FFQ	Self-reported diagnosis validated by physicians (diabetes)

Mursu,2014 (41)	Europe (Finland)	KIHD (men)	2,332 / 432	19.3	Fruit and berry juices	Unspecified	53	26.6	FFQ	Self-reported diagnosis confirmed by record linkage to the national hospital discharge registry and to the Social Insurance Institution of Finland reimbursement register on diabetes-medication expenses (diabetes)
O'Connor, 2015 (42)	Europe (UK)	The EPIC-Norfolk study (45% men, 55% women)	25,639 / 847	10.8	All types	Specified	58.7	26.3	FFQ	Self reported diagnosis and external sources (national diabetes registers and Office of National Statistics mortality) (diabetes)
Odegaard, 2009 (43)	Asia (China)	The Singapore Chinese Health Study (48% men, 52% women)	43,580 / 2,273	5.7	All types	Unspecified	54.8	23.1	FFQ	At baseline self reported diagnosis; At follow-up hospital records and biomarkers (diabetes)
Palmer,2008 (44)	USA	BWHS (women)	43,960 / 2,713	7.7	Orange or grapefruit juice	Specified	38.2	27.6	FFQ	Self-reported diagnosis (diabetes)
Scheffers, 2020 (45)	Europe (Netherlands)	MORGEN & Prospect cohort (26% men, 74% women)	36,147 / 1,477	14.6	All types	Specified	49	25.6	FFQ	Self-reported diagnosis confirmed by record linkage to the national hospital Register or validated by physicians (diabetes)

BWHS: Black Women's Health Study, CARDIA: The Coronary Artery Risk Development in Young Adults study, E3N: French Etude Epidemiologique aupres des femmes de la Mutuelle Generale de l'Education Nationale–European Prospective Investigation into Cancer and Nutrition cohort data, EPIC: European Prospective Investigation into Cancer and Nutrition study, HPFS: Health Professionals Follow-up Study, KIHD: Kuopio Ischaemic Heart Disease Risk Factor Study, MORGEN: Monitoring Project on Chronic Disease Risk Factors, NHS: Nurses' Health Study

Supplemental Table 6. Characteristics of the randomised controlled trials included in the meta-analysis

First author, year (ref)	Country	Total number of participants (men/women)	Selected features of the study participants	Duration of intervention (weeks)	Type of Juice and Comparison	Juice characteristics (as reported by the authors of the study)	Age (yrs)	BMI (kg/m^2)	Outcomes	Study design
Bakuradze,2019 (46)	Europe (Germany)	57 (57/0)	Healthy subjects	8	Mix juice (750 ml/day)	100% fruit content - red grape juice, lingonberry juice from concentrate, apple, blueberry and strawberry puree, aronia juice from concentrate, acerola puree. Glucose: 65.2 g/l, Fructose: 70.5 g/l Sucrose: 3.8 g/l Polyphenols: 3.6 g/l Anthocyanins: 274 mg/l Vitamin C: 564 mg/l	23	23	BW,LP	PA
					Placebo (750 ml/day)		24	23		

Basu (I), 2010 (47)	North America (USA)	I: 25 (2/23)	Obese participants with metabolic syndrome	8	Blueberry juice (480 ml/day) + water (480 ml/day)	50 g freeze-dried blueberries, water, vanilla extract.<br>Energy: 174 kcal<br>Protein: 1.7 g<br>Carbohydrates: 42.3 g<br>Sugars: 30.0 g<br>Fibre: 9.3 g<br>Polyphenols: 1624 mg<br>Anthocyanins: 742 mg<br>Vitamin C: 86 mg<br>Potassium: 204 mg<br>Sodium: 8 mg	51.5	38.1	BP,BW,LP,GP	PA-SB
		C: 23 (2/21)			Water (960 ml/day)		48.0	37.5		
Basu (II), 2010 (48)	North America (USA)	I: 15 (0/15)	Metabolic syndrome	8	Strawberry (480 ml/day) + water (480 ml/day)	50 g freeze-dried strawberry powder, water, vanilla essence.<br>Energy: 150 kcal<br>Carbohydrates: 33 g<br>Protein: 3.5 g<br>Fat: 0.5 g<br>Fibre: 8%<br>Polyphenols: 2006 mg<br>Anthocyanins: 154 mg<br>Vitamin C: 109 mg<br>Phytosterols: 50 mg	48.0	39.0	BP,BW,LP,GP	PA
		C: 12 (2/10)			Water (960 ml/day)		45.0	36.4		

Basu (III), 2011 (49)	North America (USA)	I: 15 (0/15)	Metabolic syndrome	8	Cranberry juice (480 ml/day)	27% cranberry juice with water, sucralose and acesulfame K (commercially available).<br>Energy: 40 kcal<br>Fructose: 4.8 g<br>Glucose: 1.8 g<br>Sucrose: 0.1 g<br>Polyphenol: 229 mg<br>Anhocyanins: 12.4 mg<br>Vitamin C: 60 mg	52.0	40.0	BP,LP,GP	PA-DB
		C: 16 (0/16)			Placebo (480 ml/day)					
Buscemi,2012 (50)	Europe (Italy)	19 (10/9)	Increased cardiovascular risk, without diabetes	1	(Red) Orange Juice (500 ml/day)	100% red orange juice.<br>Polyphenols: 419 mg/l<br>Anthocyanins: 71.3 mg/l<br>Narirutin: 43 mg/l<br>Hesperidin: 319 mg/l<br>Vitamin C: 481 mg/l<br>Total carotenoids: 5.5 mg/l	48.0	32.1	BW,GP,FMD	CO-SB
					Placebo (500 ml/day)					
Cerdà, 2006 (51)	Europe (Spain)	I:15 (15/0)	Chronic obstrutive pulmonary disease	5	Pomegranate juice (400 ml/day)	100% pomegranates juice.<br>Polyphenols: 2.66 g<br>Anthocyanins: 0.19 g	60.0	31.4	LP,GP	PA-DB
		C:15 (15/0)			Placebo (400 ml/day)		63.4	30.6		

Chai, 2018 (52)	North America (USA)	I: 20 (8/12)	Older subjects	12	Tart cherry juice (480 ml/day)	Montmorency tart cherry concentrate (68 ml) diluted with water (412 ml) (commercially available)<br>Energy: 181 kcal<br>Carbohydrate: 43 g<br>Protein: 2.3 g<br>Fibre: -<br>Fat: -<br>Polyphenols: 450.6 mg<br>Total tannins: 95.9 mg<br>Thiamin: 11 mcg<br>Folic acid: 18.6 mcg<br>Potassium: 355 mg	70.0	28.5	BP,BW,LP,GP	PA-DB
		C: 17 (9/8)			Placebo (480 ml/day)		69.5	27.3		
Dohadwala (I), 2010 (53)	North America (USA)	64 (44/20)	Pre- and stage 1 hypertension	8	Concord Grape juice (7 mL/Kg/day)	100% concord grape juice.<br>Energy: 160 kcal/240 ml<br>Sugars: 39 g/240 ml (52% fructose, 48% glucose)<br>Polyphenols: 472.8 mg/240 ml	43	28	AS,BP,BW,LP,GP	CO-DB
					Placebo<br>(7 ml/Kg/day)					
Dohadwala (II), 2011 (54)	North America (USA)	44 (30/14)	Stable coronary artery disease	4	Cranberry juice (480mL/day)	54% cranberry juice (commercially available).<br>Energy: 40 kcal<br>Carbohydrates: 10 g<br>Polyphenols: 825 mg<br>Anthocyanins: 94 mg	62	29.5	AS,BP,LP,GP, FMD	CO-DB

					Placebo<br>(480 ml/day)					
Duthie, 2006 (55)	Europe (Scotland)	I: 11 (0/11)	Healthy subjects	2	Cranberry juice (750 ml/day)	Cranberry juice (commercially available<br>Polyphenols 1136 mg/l<br>Anthocyanins 2.80 mg/l<br>Vitamin C: 897 mg/l	27.3	-	LP	PA
		C: 9 (0/9)			Placebo (750 ml/day)		28.3	-		
Flammer, 2013 (56)	North America (USA)	I: 32 (20/12)	Patients with endothelial dysfunction and cardiovascular risk factors or previous cardiovascular events	16	Cranberry juice (460 ml/day)	54% cranberry juice with water, fructose and sucralose (commercially available).<br>Fructose: 4.9 mg/ml<br>Glucose: 15.3 mg/ml<br>Sucrose: 0.6 mg/ml<br>Polyphenols: 1740 mcg/ml<br>Anthocyanins: 151 mcg/ml<br>ProAnthocyanidins: 2662 mcg/ml	44.8	27.7	BP,LP	PA-DB
		C: 37 (11/26)			Placebo (460 ml/day)		51.4	27.2		

Gonzalez-Ortiz, 2011 (57)	South America (Mexico)	I: 10 (NR)	Obese participants	4	Pomegranate juice (120 ml/day)	Pomegranate juice (commercially available).<br>Energy: 160 kcal/240 ml<br>Carbohydrate: 39 g/240 ml<br>Fibre: -<br>Sugars: 33 g/240 ml<br>Protein: 1 g/240 ml<br>Fats: -<br>Vitamin C: 4 mg/240 ml<br>Folate: 60 mcg/240 ml<br>Potassium: 490 mg/240 ml<br>Sodium: 20 mg/240 ml	36.3	35.2	BW,LP,GP	PA-DB
		C: 10 (NR)			Placebo (120 ml/day)		38.3	33.8		
Guo, 2014 (58)	Asia (China)	44 (12/32)	Young adult participants with fatty liver disease	4	Bayberry juice (500 ml/day)	100% bayberry juice.<br>Sugars: 9.2 g/100 ml<br>Polyphenols: 270.2 mg/100 ml<br>Anthocyanins: 83.5 mg/100 ml<br>Vitamin C: 98.6 mg/100 ml	21.2	25.4	BP,BW,LP,GP	CO-DB
					Placebo (500 ml/day)					

Hollis, 2015 (59)	North America (USA)	I: 25 (NR)	Overweight participants	12	Concord grape juice (480 ml/day)	100% concord grape juice.<br>Energy: 175 kcal<br>Sugars: 41 g/240 ml (52% fructose, 48% glucose)<br>Polyphenols: 1945 mg/l<br>Anthocyanins 398 mg/l<br>ProAnthocyanidins: 639 mg/l	22	27.0	BW,LP,GP	PA-DB
		C: 26 (NR)			Placebo (480 ml/day)		26	27.0		
Karlsen, 2010 (60)	Europe (Norway)	I: 31 (21/10)	Subjects at increased risk of cardiovascular disease	4	Bilberry juice (330 ml/day+670 ml of water)	100% bilberry juice diluted with water.	53	25.6	BW,LP	PA
		C: 31 (25/6)			Water (1000 ml/day)		53	25.5		
Khan, 2014 (61)	Europe (UK)	I: 22 (15/7)	Healthy subjects	6	(Low) Blackcurrant juice (1000 ml/day)	6.4 blackcurrant juice diluted with water<br>Energy: 10.4 kcal/250 ml<br>Carbohydrate 1.7 g/250 ml<br>Sugars: 1.2 g/250 ml<br>Protein: 0.1 g/250 ml<br>Polyphenols: 27.3 mg/100 ml<br>Anthocyanins: 4 mg/100 ml<br>Vitamin C: 1.1 mg/100 ml	55	28.4	BP,BW,LP,FMD	PA-DB

			I: 21 (13/8)			(High) Blackcurrant juice<br>(1000 ml/day)	20% blackcurrant juice diluted with water.<br>Energy: 10.4 kcal/250 ml<br>Carbohydrate 1.7 g/250 ml<br>Sugars: 1.2 g/250 ml<br>Protein: 0.1 g/250 ml<br>Polyphenols: 81.5 mg/100 ml<br>Anthocyanins: 14.3 mg/100 ml<br>Vitamin C: 10.2 mg/100 ml	51	29.2		
			C: 21 (15/6)			Placebo (1000 ml/day)		51	28.9		
Kojadinovic, 2016 (62)	Europe (Serbia)		I: 12 (0/12)	Metabolic syndrome	6	Pomegranate juice (300 ml/day)	100% pomegranate juice.<br>Energy: 55 kcal<br>Protein: 0.8 g/kg<br>Carbohydrates: 135 g/kg<br>Polyphenols: 2.938 g/l<br>Anthocyanins: 0.021 g/l	40-60	32	BP,BW,LP,GP	PA
			C: 11 (0/11)			Water (300 ml/day)			27.8		
Krikorian, 2012 (63)	North America (USA)	21 (11/10)	I: 10	Older adults at risk for advancing cognitive impairment	16	Concord grape juice (6.3-7.8 ml/kg)	100% concord grape juice.<br>Polyphenols: 2091 mg/l<br>Anthocyanins: 425 mg/l<br>ProAnthocyanidins: 888 mg/l	78	-	BP,BW,GP	PA – DB

		C: 11			Placebo (6.3-7.8 ml/kg)		75	-		
Loo, 2016 (64)	Europe (Finland)	38 (14/24)	Untreated (pre-) hypertension	8	Chokeberry juice (300 ml/day)	100% chokeberry juice mixed with oven-dried chokeberry powder.<br>Energy: 76 kcal<br>Polyphenols: 2194 mg<br>Anthocyanins: 1024 mg<br>ProAnthocyanidins: 745 mg<br>Phenolic Acid: 367 mg	55.8	25.9	BP,BW,LP,GP	CO-SB
					Placebo (300 ml/day)					
Lynn (I), 2012 (65)	Europe (UK)	I: 24 (8/16)	Healthy subjects	4	Pomegranate juice (330 ml/day)	100% pomegranate juice.<br>Energy: 40 kcal/100 ml<br>Carbohydrate: 10 g/100 ml<br>Polyphenols: 18.6 mmol/l<br>Potassium: 1.711 mg/l	36.1	25.0	AS,BP,BW	PA
		C: 24 (8/16)			Control drink (330 ml/day)		39.0	25.0		

Lynn (II), 2014 (66)	Europe (UK)	I: 25 (9/16)	Healthy subjects	6	Tart cherry juice (250 ml/day)	Montmorency tart cherry concentrate (30 ml) diluted with water (220 ml) (commercially available).<br>Energy: 102 kcal<br>Carbohydrate: 24.5 g.<br>Protein: 1.1 g<br>Fibre: 2.6 g<br>Fat: trace<br>Anthocyanins: 273.5 mg<br>Vitamin C: 9.72 mg<br>B-carotene: 408 mcg<br>Sodium: trace	38.3	24.6	AS,BP,LP	PA
		C: 21 (8/13)			Control drink (250 ml/day)		37.2	23.5		
Moazzen, 2017 (67)	Asia (Iran)	32 (14/18) [30 included in the final analysis)	Metabolic syndrome	1	Pomegranate juice (500 ml/day)	100% pomegranate juice.<br>Polyphenols: 69 mg/l<br>Anthocyanins: 100.46 mg/l.<br>Flavonoids: 283.02 mg/l	51.6	-	BP,LP,GP	CO-DB
					Placebo (500 ml/day)					

Morand, 2011 (68)	Europe (France)	24 (24/0)	Overweight participants	4	Orange juice (500 ml/day)	Orange juice from concentrate.<br>Energy: 194 kcal<br>Total Carbohydrates: 45 g (50%sucrose, 25% glucose, 25% fructose)<br>Flavonoids: 341.9 mg (Hesperidin: 292 mg, Narirutin: 47.5 mg)<br>Vitamin C: 180 mg<br>α-tocopherol: 1.05 mg<br>Vitamin B-9: 0.085 mg<br>Total carotenoids: 0.135 mg	56	27.4	BP,LP,GP	CO
					Placebo (500 ml/day)					
Neto, 2017 (69)	South America (Brazil)	I: 14 (5/9)	Hypertension	4	(red) Grape juice (men 150 ml/day –women 100 ml/day)	100% red grape juice	50.5	25.5	BP	PA-DB
		C: 12 (4/8)			Control drink (not specified)		53.3	26.5		

Novotny, 2015 (70)	North America (USA)	I: 29 (14/15)	Healthy subjects	8	Cranberry juice (480 ml/day	Cranberries juice (commercially available).<br>Energy: 40 kcal<br>Sugars: 6.5 (Sucrose: 0.1 g, Fructose: 4.6, Dextrose: 1.8 g)<br>Polyphenols: 173 mg<br>Anthocyanins: 10.3 mg<br>ProAnthocyanidins: 118 mg<br>Vitamin C: 60 mg	49.8	27.8	BP,LP,GP	PA-DB
		C: 27 (12/15)			Placebo (480 ml/day)		51.3	29.1		
Paquette, 2017 (71)	North America (Canada)	I: 20 (9/11)	Overweight/obese and insulin resistant individuals	6	Strawberry-cranberry juice (120 ml/day)	Dry strawberry and cranberry polyphenol extracts with water and sucralose.<br>Polyphenols: 333 mg<br>ProAnthocianidins: 20040 mcg/120 ml	57	31	BP,BW,LP	PA-DB
		C: 21 (9/12)			Placebo (120 ml/day)		60	31		
Park, 2009 (72)	Asia (Korea)	I: 21 (21/0)	(Pre-) Hypertension	8	Grape Juice (5.5ml/kg/day)	Grape juice.<br>Polyphenols: 2108 mg/l	43	26.5	BP,BW,LP	PA-DB
		C: 19 (19/0)			Placebo (5.5 ml/kg/day)		46	26.2		

Ruel, 2013 (73)	North America (Canada)	35 (35/0)	Healthy overweight subjects	4	Cranberry juice (500 ml/day)	27% cranberry juice with water and sucralose (commercially available).<br>Energy: 22 kcal/125 ml<br>Carbohydrates: 5.46 g/125 ml<br>Polyphenols: 100 mg/125 ml<br>Anthocyanins: 5.2 mg/100 ml<br>ProAnthocyanidins: 74 mg/125 ml<br>Vitamin C: 32 mg/125 ml	45	28.3	BP	CO-DB
					Placebo (500 ml/day)					
Shidfar, 2012 (74)	Asia (Iran)	I: 29 (29/0)	Type 2 diabetes patients	12	Cranberry juice (240 ml/day)	100% Cranberry juice	54.8	29.1	GP	PA-DB
		C: 29 (29/0)			Placebo (240 ml/day)			28.4		
Siasos, 2014 (75)	Europe (Greece)	26 (10/16)	Healthy smokers	2	Concord grape juice (7 ml/kg/day)	100% concord grape juice (commercially available).<br>Energy: 160 kcal/240 ml<br>Sugars: 39 g/240 ml (52% Fructose, 48% Glucose)<br>Polyphenols: 472.8 mg/240 ml<br>Anthocyanins: 296 mcmol/l / 240 ml	26.3	23.2	AS,BP,BW,LP, GP,FMD	CO-DB
					Placebo (7 ml/kg/day)					

Silver, 2011 (76)	North America (USA)	I: 28 (3/25)	Obese subjects (BMI: 30-39.9 kg/m^2)	12	Grapefruit juice (≈121 ml/day)	100% grapefruit juice.<br>Energy: 46 kcal<br>Fibre: 0.13 g<br>Flavonoid (Naringin): 39.6 mg<br>Vitamin C: 48.3 mg	39.8	35.2	BP,BW,LP,GP	PA
		C: 28 (7/21)			Water (≈127 g ml/day)		38.7	35.7		
Simpson, 2016 (77)	Europe (UK)	I: 18 (18/0)	Overweight/obese, hypercholesterolemic subjects	12	Orange juice (250 ml/day)	100% orange juice.<br>Sucrose: 10.78 g<br>Fructose: 5.60 g<br>Glucose: 5.25 g<br>Hesperidin: 135.4 mg<br>Narirutin: 15.5 mg<br>Vitamin C: 137 mg	48.3	29.9	BW,LP,GP	PA-SB
		C: 18 (18/0)			Placebo (250 ml/day)		48.9	29.3		
Sohrab (I), 2014 (78) & (II) 2015 (79)	Asia (Iran)	22 (11/11)	Type 2 diabetes (without insulin as medication)	12	Pomegranate juice (250 ml/day)	Pomegranate juice.<br>Energy: 126 kcal<br>Sugars: 24 g<br>Protein: 1.4 g<br>Fibre: 0 g<br>Polyphenols: 1946 mg/l<br>Flavonoids: 345.87 mcg/ml	55	29.4	BW,GP	PA-DB
		22 (11/11)			Placebo (250 ml/day)		56.9	28.6		

Sumner, 2005 (80)	North America (USA)	I: 26 (22/4)	Coronary heart disease	13	Pomegranate juice (240 ml/day)	Pomegranate juice (commercially available).<br>Energy: 160 kcal<br>Carbohydrate: 39 g<br>Sugars: 34 g<br>Fibre: -<br>Protein: -<br>Fats: -<br>Potassium: 550 mg<br>Sodium: 5 mg	69	28	BP,BW,LP,GP	PA-DB
		C: 19 (18/1)			Placebo (240 ml/day)		69	29		
Tsang, 2012 (81)	Europe (UK)	28 (12/16)	Apparently healthy overweight/obese subjects	4	Pomegranate juice (500 ml/day)	100% pomegranate juice.<br>Energy: 15 kcal/100 ml<br>Carbohydrate: 3.4 g/100 ml<br>Sugars: 3.4 g/100 ml<br>Protein: 0.1 gr/100ml<br>Fats: -<br>Polyphenols: 1685 mg/l<br>Vitamin C: 20 mg/100 ml<br>Sodium: -	50.4	26.8	AS,BP,LP,GP	CO
					Placebo (500 ml/day)					

AS: arterial stiffness, BP: blood pressure, BW: body weight, C: control group, CO: cross-over, DB: double-blind, FMD: flow-mediated dilation, GP: glucose profile, I: intervention group, LP: lipids profile, PA: parallel-arm, SB: single-blind.

Supplemental Table 7. Studies excluded after qualitative analysis

1. Aghababaee SK et al. Res Med Sci 2015, 20(7):684-691.

2. Banini AE et al. Nutrition. 2006 Nov-Dec;22(11-12):1137-45.

3. Lima ACD et al. J Med Food. 2019 Feb;22(2):202-210. doi: 10.1089/jmf.2018.0080

4. Ribeiro C et al. Nutrition 2017, 38;13-19

5. Ravn-Haren G et al. Eur J Nutr 2013, 52:1875-1889

6. Sohrab G et al. Clinical Nutrition ESPEN 2019, 29:30-35

7. Zare Javid A et al. J Am Coll Nutr. 2018 Jan;37(1):71-79. doi: 10.1080/07315724.2017.1357509.

8. Cesar TB et al. Nutr Res. 2010 Oct;30(10):689-94. doi: 10.1016/j.nutres.2010.09.006.

9. Aviram M et al. Clin Nutr 2004;23:423-33

10. Stein JH et al. Circulation. 1999 Sep 7;100(10):1050-5

11. Lazavi F et al. Complementary Therapies in Clinical Practice 2018, 31:170-174

12. Asgary S et al. Phytother Res 2014,28:193-199

13. Leelarungrayub J et al. Clin Interv Aging. 2016 Aug 31;11:1183-92. doi: 10.2147/CIA.S110718

14. Toth PP et al. Front Pharmacol. 2016 Jan 6;6:299. doi: 10.3389/fphar.2015.00299

15. Mulero J et al. Clin Nutr. 2012 Jun;31(3):372-7. doi: 10.1016/j.clnu.2011.11.014

16. Diáz-Juárez JA et al. Phytother Res 2009, 23:948–54

17. Kardum N et al. J Med Food 00 (0) 2015, 1–8. doi: 10.1089/jmf.2014.0171

18. Poreba M et al. Ann Agric Environ Med. 2009;16(2):305-8.

19. Chou EJ et al. Am J Cardiol. 2001 Sep 1;88(5):553-5.

20. Johnson SA et al. J Acad Nutr Diet 2015;115:369-377. http://dx.doi.org/10.1016/j.jand.2014.11.001

21. Ellinger S et al. J Agric Food Chem. 2012 Nov 14;60(45):11292-300. doi: 10.1021/jf300719t

22. Amagase H et al. Nutrition Research 29 2009, 19–25. doi:10.1016/j.nutres.2008.11.005

23. Duthie SJ et al. Eur J Nutr (2018) 57:1855–1872. https://doi.org/10.1007/s00394-017-1469-0

24. Hollands WJ et al. British Journal of Nutrition 2018, 119:415-421.

25. Kent K et al. Eur J Nutr 2017,56:333-341.

26. Sweazea KL et al. J Nutr Metab. 2018 Sep 16;2018:1729653. doi: 10.1155/2018/1729653

27. Soriano-Maldonado A et al. Eur J Nutr 2014, 53:1645–1657. doi 10.1007/s00394-014-0670-7

28. Guo C et al. Nutrition Research 28 (2008) 72–77. doi:10.1016/j.nutres.2007.12.001

29. Bub A et al. Journal of Nutritional Biochemistry 14 (2003) 90–98.

30. Razani Z et al. Phytother Res 2017,31:1731-1738.

31. Mathison BD et al. Nutr Res. 2014 May;34(5):420-7. doi: 10.1016/j.nutres.2014.03.006.

32. Kunz S et al. British Journal of Nutrition 2014, 112, 925–936. doi:10.1017/S0007114514001482

33. Chai SC et al. Nutrients 2019, 11, 228; doi:10.3390/nu11020228

34. Shema-Didi L et al. Free Radical Biology and Medicine 2012, 53:297-304.

35. Sohrab G et al. Metab Syndr Relat Disord. 2018 Oct;16(8):446-451

36. Iso H, Kubota Y. Asian Pac J Cancer Prev 2007;(Suppl 8):35–80.

37. Oyebode O et al. J Epidemiol Community Health 2014,68:856-862.

38. Pase MP et al. Appetite 84 (2015) 68–72. http://dx.doi.org/10.1016/j.appet.2014.09.019

39. Aptekmann NP et al. Lipids Health Dis. 2013 Aug 6;12:119. doi: 10.1186/1476-511X-12-119.

40. Oude Griep LM et al. Am J Clin Nutr 2013;97:1083–91.013

41. Eshak ES et al. Clinical Nutrition 32 (2013) 300-308. http://dx.doi.org/10.1016/j.clnu.2012.08.003

42. Johnsen SP et al. Am J Clin Nutr 2003,78:57-64.

43. Oude Griep LM et al. PLoS ONE 2010,5(10):e13609.

44. Oude Griep LM et al. Eur J Clin Nutr 2011,65:791-799.

45. Shema-Didi L. et al. Nutrition Journal 2014, 13:18.

46. Reshef N et al. Am J Hypertens. 2005 Oct;18(10):1360-3.

47. Costans J et al. Clinical Nutrition 2015, 34,1093-1100.

48. Habauzit V et al. Am J Clin Nutr 2015; 102:66-72.

49. Tjelle T et al. British Journal of Nutrition 2015,114:1054-63.

50. Pokimika B et al. Nutrients. 2019 Apr 15;11(4). pii: E850. doi: 10.3390/nu11040850

Supplemental Table 8. Subgroup analysis and meta-regression analysis of the effect of fruit juice consumption on blood pressure (RCTs)

A. Systolic Blood Pressure				
	Variables (n. of cohorts)	Mean Difference (mm Hg)	95% CI	P for interaction
**Study design**	Cross-over (9)	-3.47	-5.42 to -1.51	0.51
	Parallel-arm (17)	-2.69	-4.54 to -0.85	
**Type of blinding**	Double-blind (17)	-3.59	-5.61 to -1.56	0.85
	Single-blind (2)	-2.89	-5.95 to 0.17	
	Open-label (7)	-2.81	-4.81 to -0.81	
**Risk factors**	Healthy (9)	-3.77	-6.08 to -1.46	0.44
	Overweight/obese (4)	-1.51	-4.28 to 1.25	
	With cardio-metabolic risk factors (10)	-3.70	-5.63 to -1.77	
	At high cardiovascular risk (3)	0.56	-7.58 to 8.71	
**Type of Juice**	Pomegranate Juice (5)	-5.25	-7.60 to -2.90	0.56
	Cranberry Juice (5)	-3.03	-7.51 to 1.45	
	Grape Juice (5)	-0.89	-5.25 to 3.47	
	Blackcurrant Juice (2)	4.59	-4.45 to 13.63	
	Cherry Juice (2)	-3.67	-8.05 to 0.71	
	Strawberry Juice (1)	2.20	-12.11 to 16.51	
	Blueberry Juice (1)	-5.80	-13.25 to 1.65	
	Chokeberry Juice (1)	-2.30	-5.65 to 1.05	
	Bayberry Juice (1)	-4.00	-11.84 to 3.84	
	Orange Juice (1)	-3.00	-8.29 to 2.29	
	Grapefruit Juice (1)	-1.60	-5.28 to 2.08	
	Mix Juice (1)	2.00	-10.54 to 14.54	
**Comparator**	Placebo (19)	-3.37	-5.01 to -1.73	0.74
	Water (4)	-2.00	-5.16 to 1.17	
	Other control drink (3)	-3.36	-6.15 to -0.57	

**Fruit Juice energy content compared to control drink**	Iso-caloric (19)	-3.58	-5.02 to -2.14	0.40
	Hyper-caloric (6)	-1.28	-4.27 to 1.71	
	Unspecified (1)	-2.60	-15.30 to 10.10	
**Diet during trial**	Usual (15)	-3.76	-5.39 to -2.13	0.13
	Without confounder sources (9)	-1.54	-4.23 to 1.15	
	Controlled diet (1)	-11.30	-20.32 to -2.28	
	Restricted energy intake (1)	-1.60	-5.28 to 2.08	
**Country of origin**	Noth America (13)	-1.99	-4.22 to 0.24	0.18
	Europe (9)	-2.70	-4.59 to -0.82	
	East Asia (2)	-3.92	-10.95 to 3.11	
	Asia-Middle East (1)	-6.94	-10.27 to -3.61	
	South America (1)	-2.60	-15.30 to 10.10	
**Reconstituited Fruit Juice**	Yes (14)	-2.54	-4.44 to -0.64	0.40
	No (12)	-3.65	-5.41 to -1.89	

B. Systolic Blood Pressure			
**Variables** (n. of cohorts)	**Coefficient**	**95% CI**	
**Age (years) (26)**	-.0128715	-.1684479	.1427049
**BMI (Kg/m²) (24)**	.0785245	-.259822	.416871
**Length of intervention (week) (26)**	.3325463	-.0353023	.7003949
**Number of participants (n) (26)**	.0678393	-.0476276	.1833062
**Gender (% men) (26)**	.0232193	-.0379473	.084386
**Year of publication (year) (26)**	-.4346865	-.9601755	.0908025
**Volume of Fruit Juice (ml/day) (26)**	-.0006617	-.0094829	.0081595
**Baseline systolic blood pressure (mm Hg) (24)**	-.0570601	-.2110391	.096919
**Baseline diastolic blood pressure (mm Hg) (24)**	-.1410262	-.3325957	.0505434
**Risk of bias (point) (26)**	.5399205	-1.441215	2.521056

C. Diastolic Blood Pressure				
	**Variables (n. of cohorts)**	**Mean Difference (mm Hg)**	**95% CI**	**P for interaction**
**Study design**	Cross-over (9)	-1.44	-3.57 to 0.69	0.49
	Parallel-arm (17)	-2.21	-3.61 to -0.82	
**Type of blinding**	Double-blind (17)	-0.90	-2.64 to 0.84	0.03
	Single-blind (2)	-0.71	-4.51 to 3.09	
	Open-label (7)	-3.33	-5.10 to -1.55	
**Risk factors**	Healthy (9)	-2.94	-4.76 to -1.13	0.09
	Overweight/obese (4)	-2.69	-5.93 to 0.54	
	With cardio-metabolic risk factors (10)	-0.51	-2.65 to 1.63	
	At high cardiovascular risk (3)	1.36	-3.11 to 5.83	
**Type of Juice**	Pomegranate Juice (5)	-2.87	-5.38 to -0.36	0.09
	Cranberry Juice (5)	-0.78	-5.08 to 3.52	
	Grape Juice (5)	0.90	-2.55 to 4.34	
	Blackcurrant Juice (2)	2.47	-7.19 to 12.13	
	Cherry Juice (2)	-2.54	-5.94 to 0.86	
	Strawberry Juice (1)	-1.10	-8.35 to 6.15	
	Blueberry Juice (1)	-3.20	-7.71 to 2.31	
	Chokeberry Juice (1)	0.80	-1.47 to 3.07	
	Bayberry Juice (1)	0.10	-5.19 to 5.39	
	Orange Juice (1)	-5.50	-9.03 to -1.97	
	Grapefruit Juice (1)	-3.90	-8.41 to 0.61	
	Mix Juice (1)	0.00	-8.23 to 8.23	
**Comparator**	Placebo (19)	-1.34	-3.05 to 0.37	0.66
	Water (4)	-2.00	-5.57 to 1.57	
	Other control drink (3)	-2.49	-4.37 to -0.62	
**Fruit Juice energy content compared to control drink**	Iso-caloric (19)	-1.73	-3.19 to -0.28	0.69
	Hyper-caloric (6)	-1.92	-4.76 to 0.93	
	Unspecified (1)	2.20	-7.21 to 11.61	
**Diet during trial**	Usual (15)	-1.66	-3.21 to -0.12	0.05

	Without confounder sources (9)	-0.71	-2.74 to 1.32	
	Controlled diet (1)	-8.20	-13.30 to -3.10	
	Restricted energy intake (1)	-3.90	-8.41 to 0.61	
**Country of origin**	Noth America (13)	-1.14	-3.03 to 0.75	0.31
	Europe (9)	-1.91	-4.18 to 0.36	
	East Asia (2)	-0.45	-5.29 to 4.40	
	Asia-Middle East (1)	-4.00	-6.35 to -1.65	
	South America (1)	2.20	-7.21 to 11.61	
**Reconstituited Fruit Juice**	Yes (14)	-1.33	-2.61 to -0.05	0.23
	No (12)	-2.45	-3.75 to -1.16	

D. Diastolic Blood Pressure			
**Variables** (n. of cohorts)	**Coefficient**	**95% CI**	
**Age (years) (26)**	.0125207	-.1256053	.1506467
**BMI (Kg/m^2) (24)**	-.028484	-.3786708	.3217027
**Length of intervention (week) (26)**	.2442633	-.1359238	.6244504
**Number of participants (n) (26)**	.0208782	-.0949261	.1366825
**Gender (% men) (26)**	-.0018935	-.0552538	.0514669
**Year of publication (year) (26)**	.0520775	-.4915935	.5957484
**Volume of Fruit Juice (ml/day) (26)**	-.0000815	-.0096185	.0094555
**Baseline systolic blood pressure (mm Hg) (24)**	-.0381115	-.1976627	.1214397
**Baseline diastolic blood pressure (mm Hg) (24)**	-.0893083	-.3175527	.1389361
**Risk of bias (point) (26)**	-.1527052	-1.695538	1.390128

Supplemental Table 9. Subgroup analysis and meta-regression analysis of the effect of fruit juice consumption on body weight

A. **Weight**

	Variables (n. of cohorts)	Mean Difference (Kg)	95% CI	P for interaction
**Study design**	Cross-over (5)	0.00	-0.44 to 0.44	0.66
	Parallel-arm (15)	-0.15	-0.61 to 0.32	
**Type of blinding**	Double-blind (14)	-0.01	-0.45 to 0.43	0.27
	Single-blind (2)	-0.70	-1.55 to 0.15	
	Open-label (4)	0.11	-0.46 to 0.67	
**Risk factors**	Healthy (6)	0.07	-2.65 to 2.80	0.29
	Overweight/obese (4)	-0.70	-1.55 to 0.14	
	With cardio-metabolic risk factors (10)	0.03	-0.31 to 0.38	
**Type of Juice**	Grape Juice (5)	-0.35	-4.72 to 4.01	0.97
	Pomegranate Juice (3)	-0.57	-8.40 to 7.27	
	Blackcurrant Juice (2)	-0.27	-12.82 to 12.29	
	Orange Juice (2)	-0.18	-1.77 to 1.41	
	Mix Juice (2)	0.82	-4.52 to 6.15	
	Bilberry Juice (1)	0.10	-0.47 to 0.67	
	Cherry Juice (1)	-0.40	-9.20 to 8.40	
	Strawberry Juice (1)	0.10	-20.09 to 20.29	
	Blueberry Juice (1)	-0.90	-1.90 to 0.10	
	Chokeberry Juice (1)	-0.01	-0.45 to 0.44	
	Bayberry Juice (1)	-0.05	-3.58 to 3.48	
**Comparator**	Placebo (17)	-0.02	-0.44 to 0.40	0.70
	Water (3)	-0.27	-1.03 to 0.50	
**Fruit Juice energy content compared to control drink**	Iso-caloric (13)	-0.02	-0.44 to 0.41	0.91
	Hyper-caloric (6)	-0.14	-0.64 to 0.35	
	Unspecified (1)	-1.60	-15.59 to 12.39	
**Diet during trial**	Usual (14)	-0.16	-0.55 to 0.23	0.45
	Without confounder sources (6)	0.10	-0.45 to 0.66	

**Country of origin**	Noth America (8)	-0.87	-1.84 to 0.10	0.56
	Europe (8)	0.03	-0.31 to 0.37	
	East Asia (2)	-0.11	-3.44 to 3.22	
	Asia-Middle East (1)	0.80	-11.55 to 13.15	
	South America (1)	-1.60	-15.59 to 12.39	
**Reconstituited Fruit Juice**	Yes (9)	-0.06	-0.39 to 0.27	0.86
	No (11)	-0.19	-1.55 to 1.17	

B. **Weight**			
**Variables** (n. of cohorts)	**Coefficient**	**95% CI**	
**Age (years) (20)**	.0175886	-.0604191	.0955962
**Length of intervention (week) (20)**	-.0530894	-.2215645	.1153856
**Number of participants (n) (20)**	.0056812	-.0404664	.0518287
**Gender (% men) (19)**	.007519	-.0071001	.022138
**Year of publication (year) (20)**	.0379438	-.128567	.2044546
**Volume of Fruit Juice (ml/day) (20)**	-.0028055	-.0080116	.0024007
**Energy intake by Fruit Juice (Kcal/day) (11)**	-.0068712	-.0179158	.0041734
**BMI (Kg/m^2) (19)**	-.0779219	-.1711375	.0152938
**Baseline weight (Kg) (18)**	-.0050476	-.0897152	.07962
**Baseline waist circumference (cm) (7)**	.002957	-.1127288	.1186429
**Risk of bias (point) (20)**	.2748022	-.10858	.6581845

C. BMI				
	**Variables (n. of cohorts)**	**Mean Difference (Kg)**	**95% CI**	**P for interaction**
**Study design**	Cross-over (2)	-0.02	-0.18 to 0.13	0.91
	Parallel-arm (11)	-0.04	-0.24 to 0.16	
**Type of blinding**	Double-blind (10)	-0.02	-0.18 to 0.13	0.94
	Open-label (3)	-0.03	-0.24 to 0.17	
**Risk factors**	Healthy (5)	-0.06	-0.27 to 0.15	0.77
	Overweight/obese (3)	0.18	-0.47 to 0.83	
	With cardio-metabolic risk factors (5)	-0.02	-0.18 to 0.14	
**Type of Juice**	Pomegranate Juice (4)	-0.06	-0.27 to 0.16	0.99
	Grape Juice (2)	-0.12	-1.35 to 1.11	
	Blackcurrant Juice (2)	-0.02	-3.92 to 3.89	
	Cherry Juice (1)	-0.10	-2.90 to 2.70	
	Chokeberry Juice (1)	-0.02	-0.18 to 0.14	
	Bayberry Juice (1)	-0.10	-1.08 to 0.88	
	Grapefruit Juice (1)	0.20	-0.47 to 0.87	
	Mix Juice (1)	0.00	-4.19 to 4.19	
**Comparator**	Placebo (10)	-0.02	-0.18 to 0.13	0.75
	Water (2)	0.21	-0.45 to 0.87	
	Control drink (1)	-0.06	-0.28 to 0.16	
**Fruit Juice energy content compared to control drink**	Iso-caloric (8)	-0.04	-0.16 to 0.09	0.75
	Hyper-caloric (4)	0.20	-0.45 to 0.86	
	Unspecified (1)	-0.70	-5.89 to 4.49	
**Diet during trial**	Usual (10)	-0.03	-0.16 to 0.09	0.79
	Without confounder sources (2)	-0.09	-1.05 to 0.86	
	Restricted energy intake (1)	0.20	-0.47 to 0.87	
**Country of origin**	Noth America (4)	0.13	-0.45 to 0.71	0.98
	Europe (5)	-0.03	-0.16 to 0.09	
	East Asia (2)	-0.11	-1.03 to 0.81	
	Asia-Middle East (1)	0.20	-3.33 to 3.73	

	South America (1)	-0.70	-5.89 to 4.49	
**Reconstituited Fruit Juice**	Yes (5)	-0.02	-0.18 to 0.14	0.88
	No (8)	-0.04	-0.24 to 0.16	

D. BMI

**Variables** (n. of cohorts)	**Coefficient**	**95% CI**	
**Age (years) (20)**			
**Length of intervention (week) (13)**	.0159788	-.0471108	.0790684
**Number of participants (n) (13)**	.0007242	-.0248131	.0262616
**Gender (% men) (11)**	-.005415	-.0340558	.0232258
**Year of publication (year) (13)**	.0027863	-.0666697	.0722423
**Volume of Fruit Juice (ml/day) (13)**	-.0007267	-.0033307	.0018772
**Energy intake by Fruit Juice (Kcal/day) (10)**	-.0007644	-.004615	.0030861
**Baseline weight (Kg) (9)**	.0035414	-.1126535	.1197363
**Baseline waist circumference (cm) (5)**	-.000126	-.049021	.048769
**Risk of bias (point) (13)**	-.1179087	-1.517984	1.282166

E. Waist Circumference

	Variables (n. of cohorts)	Mean Difference (cm)	95% CI	P for interaction
**Study design**	Cross-over (1)	0.00	-2.94 to 2.94	0.94
	Parallel-arm (9)	0.13	-1.00 to 1.25	
**Type of blinding**	Double-blind (5)	-0.09	-1.85 to 1.68	0.93
	Single-blind (2)	0.32	-1.17 to 1.81	
	Open-label (3)	-0.10	-2.78 to 2.58	
**Risk factors**	Healthy (2)	-0.12	-1.96 to 1.71	0.95
	Overweight/obese (5)	0.23	-1.06 to 1.52	
	With cardio-metabolic risk factors (3)	-0.29	-9.73 to 9.14	
**Type of Juice**	Pomegranate Juice (2)	0.37	-7.68 to 8.41	1.0
	Grape Juice (2)	-0.21	-2.53 to 2.11	
	Grapefruit Juice (1)	-0.10	-2.84 to 2.64	
	Bayberry Juice (1)	0.00	-2.94 to 2.94	
	Orange Juice (1)	0.50	-1.52 to 2.52	
	Strawberry Juice (1)	3.30	-24.08 to 30.68	
	Blueberry Juice (1)	0.10	-2.11 to 2.31	
	Mix Juice (1)	0.00	-10.51 to 10.51	
**Comparator**	Placebo (6)	0.17	-1.16 to 1.50	0.89
	Water (4)	0.02	-1.69 to 1.73	
**Fruit Juice energy content compared to control drink**	Iso-caloric (5)	0.15	-1.19 to 1.49	0.98
	Hyper-caloric (4)	0.02	-1.69 to 1.73	
	Unspecified (1)	1.00	-8.80 to 10.80	
**Diet during trial**	Usual (7)	0.17	-1.06 to 1.41	0.98
	Without confounder sources (2)	0.00	-2.83 to 2.83	
	Restricted energy intake (1)	-0.10	-2.84 to 2.64	
**Country of origin**	Noth America (6)	-0.05	-1.42 to 1.32	0.97
	Europe (2)	0.47	-1.53 to 2.47	
	East Asia (1)	0.00	-2.94 to 2.94	
	South America (1)	1.00	-8.80 to 10.80	

**Reconstituited Fruit Juice**	Yes (3)	0.12	-2.04 to 2.28	0.99
	No (7)	0.11	-1.09 to 1.31	

F. Waist Circumference

**Variables** (n. of cohorts)	**Coefficient**	**95% CI**	
**Age (years) (10)**	.0131549	-.0883796	.1146894
**Length of intervention (week) (10)**	.0055537	-.4106446	.421752
**Number of participants (n) (10)**	-.0282404	-.1872732	.1307923
**Gender (% men) (8)**	.0054742	-.0311236	.042072
**Year of publication (year) (10)**	.0584099	-.4710099	.5878297
**Volume of Fruit Juice (ml/day) (10)**	-.0007301	-.0091208	.0076605
**Energy intake by Fruit Juice (Kcal/day) (6)**	-.0005418	-.0170175	.0159338
**Baseline BMI (kg/m^2) (9)**	.0046407	-.2710497	.280331
**Baseline weight (Kg) (7)**	.0239721	-.1299303	.1778745
**Baseline waist circumference (cm) (8)**	.0086716	-.043332	.0606752
**Risk of bias (point) (10)**	.0803118	-.8238818	.9845053

Supplemental Table 10. Subgroup analysis and meta-regression analysis of the effect of fruit juice consumption on lipid metabolic profile

A. **Total Cholesterol**

	Variables (n. of cohorts)	Mean Difference (mg/dl)	95% CI	P for interaction
**Study design**	Cross-over (8)	-1.84	-6.83 to 3.15	0.50
	Parallel-arm (21)	-4.15	-8.51 to 0.22	
**Type of blinding**	Double-blind (17)	-3.67	-9.40 to 2.05	0.92
	Single-blind (3)	-1.87	-8.74 to 4.99	
	Open-label (9)	-3.42	-8.37 to 1.52	
**Risk factors**	Healthy (8)	-4.65	-11.83 to 2.52	0.97
	Overweight/obese (5)	-2.53	-8.41 to 3.36	
	With cardio-metabolic risk factors (15)	-2.94	-7.72 to 1.84	
	Other disease (1)	0.24	-41.51 to 41.99	
**Type of Juice**	Pomegranate Juice (6)	1.66	-8.73 to 12.05	0.95
	Cranberry Juice (5)	-0.84	-12.97 to 11.30	
	Grape Juice (4)	-1.43	-12.70 to 9.84	
	Cherry Juice (2)	-8.63	-21.76 to 4.50	
	Blackcurrant Juice (2)	1.95	-18.42 to 22.32	
	Orange Juice (2)	1.34	-13.88 to 16.56	
	Grapefruit Juice (1)	-5.70	-12.72 to 1.32	
	Bayberry Juice (1)	-12.00	-26.31 to 2.31	
	Bilberry Juice (1)	-5.79	-18.65 to 7.07	
	Strawberry Juice (1)	-19.30	-57.13 to 18.53	
	Blueberry Juice (1)	0.00	-27.24 to 27.24	
	Chokeberry Juice (1)	-1.16	-8.72 to 6.40	
	Strawberry+Cranberry Juice (1)	-6.95	-37.98 to 24.08	

	Mix Juice (1)	-4.50	-27.04 to 18.04	
**Comparator**	Placebo (23)	-1.91	-6.01 to 2.20	0.59
	Water (5)	-5.11	-10.99 to 0.77	
	Control drink (1)	-7.07	-22.20 to 8.06	
**Fruit Juice energy content compared to control drink**	Iso-caloric (19)	-2.43	-6.53 to 1.67	0.84
	Hyper-caloric (9)	-4.37	-9.91 to 1.17	
	Unspecified (1)	-7.72	-46.92 to 31.48	
**Diet during trial**	Usual (17)	-1.98	-6.83 to 2.87	0.73
	Without confounder sources (10)	-3.85	-9.99 to 2.29	
	Controlled diet (1)	3.09	-14.35 to 20.53	
	Restricted energy intake (1)	-5.70	-12.72 to 1.32	
**Country of origin**	Noth America (13)	-4.21	-9.39 to 0.96	0.51
	Europe (13)	-1.19	-5.92 to 3.55	
	East Asia (1)	-12.00	-26.31 to 2.31	
	Asia-Middle East (2)	-3.94	-17.02 to 9.14	
**Reconstituited Fruit Juice**	Yes (14)	-2.56	-7.33 to 2.20	0.74
	No (15)	-3.68	-8.21 to 0.85	

**B. Total Cholesterol**			
**Variables** (n. of cohorts)	**Coefficient**	**95% CI**	
**Age (years) (29)**	.2087322	-.0957425	.5132069
**Length of intervention (week) (29)**	-.1886155	-1.180843	.8036123
**Number of participants (n) (29)**	-.1477117	-.437016	.1415925
**Gender (% men) (27)**	.0683026	-.0594819	.1960871
**Year of publication (year) (29)**	-.2083275	-1.406807	.9901524
**Volume of Fruit Juice (ml/day) (29)**	.0065875	-.0113025	.0244775
**Energy intake by Fruit Juice (Kcal/day) (16)**	-.0003146	-.0534269	.0527978
**Baseline BMI (kg/m^2) (27)**	-.2384758	-1.03619	.5592388
**Baseline total cholesterol (mg/dl) (27)**	-.0043634	-.1456368	.1369101
**Baseline LDL-cholesterol (mg/dl) (21)**	.1017191	-.1258367	.3292749
**Baseline HDL-cholesterol (mg/dl) (24)**	.1872147	-.3189291	.6933586
**Baseline triglycerides (mg/dl) (24)**	-.0224749	-.1046788	.059729
**Risk of bias (point) (29)**	-.4889376	-5.200919	4.223044

C. LDL-Cholesterol				
	**Variables (n. of cohorts)**	**Mean Difference (mg/dl)**	**95% CI**	**P for interaction**
**Study design**	Cross-over (7)	-1.23	-6.79 to 4.33	0.53
	Parallel-arm (16)	0.87	-2.55 to 4.29	
**Type of blinding**	Double-blind (14)	-2.40	-7.33 to 2.53	0.41
	Single-blind (2)	2.89	-8.88 to 14.67	
	Open-label (7)	1.61	-2.17 to 5.40	
**Risk factors**	Healthy (5)	-4.79	-11.70 to 2.13	0.34
	Overweight/obese (5)	2.32	-1.52 to 6.15	
	With cardio-metabolic risk factors (12)	-0.89	-6.82 to 5.05	
	Other disease (1)	3.33	-32.34 to 39.00	
**Type of Juice**	Pomegranate Juice (6)	1.23	-7.85 to 10.30	0.63
	Cranberry Juice (4)	-0.03	-10.09 to 10.03	
	Grape Juice (4)	-0.78	-11.89 to 10.32	
	Orange Juice (2)	7.22	-3.97 to 18.42	
	Cherry Juice (1)	-12.50	-37.39 to 12.39	
	Grapefruit Juice (1)	1.80	-2.32 to 5.92	
	Bayberry Juice (1)	-8.20	-17.61 to 1.21	
	Strawberry Juice (1)	-15.44	-49.54 to 18.66	
	Blueberry Juice (1)	3.86	-12.80 to 20.52	
	Strawberry+Cranberry Juice (1)	-6.95	-33.43 to 19.53	
	Mix Juice (1)	-7.30	-29.06 to 14.46	
**Comparator**	Placebo (19)	-1.13	-5.43 to 3.17	0.38
	Water (4)	1.49	-2.46 to 5.45	
**Fruit Juice energy content compared to control drink**	Iso-caloric (16)	-1.07	-5.47 to 3.32	0.69
	Hyper-caloric (6)	1.42	-2.49 to 5.33	
	Unspecified (1)	-3.90	-40.16 to 32.36	
**Diet during trial**	Usual (12)	0.05	-6.33 to 6.43	0.60

	Without confounder sources (9)	-3.01	-8.88 to 2.85	
	Controlled diet (1)	2.70	-10.92 to 16.32	
	Restricted energy intake (1)	1.80	-2.32 to 5.92	
**Country of origin**	Noth America (12)	1.00	-2.47 to 4.46	0.32
	Europe (8)	1.78	-6.23 to 9.80	
	East Asia (1)	-8.20	-17.61 to 1.21	
	Asia-Middle East (2)	2.06	-9.15 to 13.28	
**Reconstituited Fruit Juice**	Yes (8)	-0.21	-7.72 to 7.30	0.89
	No (15)	0.38	-2.77 to 3.54	

D. **LDL-cholesterol**		
**Variables** (n. of cohorts)	**Coefficient**	**95% CI**
**Age (years) (23)**	.1917848	-.0982563 .4818259
**Length of intervention (week) (23)**	.3310754	-.4762682 1.138419
**Number of participants (n) (23)**	.0430864	-.237066 .3232387
**Gender (% men) (21)**	.0576498	-.0690479 .1843475
**Year of publication (year) (23)**	-.7719624	-1.983642 .4397169
**Volume of Fruit Juice (ml/day) (23)**	-.0084995	-.0245353 .0075363
**Energy intake by Fruit Juice (Kcal/day) (12)**	-.0190648	-.069871 .0317413
**Baseline BMI (kg/m^2) (21)**	.3665378	-.3596031 1.092679
**Baseline total cholesterol (mg/dl) (21)**	.0307062	-.1363101 .1977226
**Baseline LDL-cholesterol (mg/dl) (21)**	.1408803	-.042515 .3242757
**Baseline HDL-cholesterol (mg/dl) (21)**	-.0195197	-.9533382 .9142987
**Baseline triglycerides (mg/dl) (21)**	.005081	-.0719627 .0821247
**Risk of bias (point) (23)**	.1522973	-3.900806 4.205401

E. HDL-Cholesterol

	Variables (n. of cohorts)	Mean Difference (mg/dl)	95% CI	P for interaction
**Study design**	Cross-over (7)	-0.91	-2.18 to 0.36	0.02
	Parallel-arm (18)	1.55	-0.05 to 3.16	
**Type of blinding**	Double-blind (14)	-0.01	-1.97 to 1.94	0.002
	Single-blind (3)	-0.73	-1.92 to 0.47	
	Open-label (8)	4.39	0.96 to 7.82	
**Risk factors**	Healthy (5)	4.49	-0.29 to 9.27	0.004
	Overweight/obese (5)	3.59	0.11 to 7.07	
	With cardio-metabolic risk factors (14)	-0.68	-1.73 to 0.37	
	Other disease (1)	-2.31	-21.91 to 17.29	
**Type of Juice**	Pomegranate Juice (6)	-0.61	-3.38 to 2.15	0.06
	Cranberry Juice (4)	-0.06	-3.94 to 3.82	
	Grape Juice (3)	1.35	-3.43 to 6.13	
	Orange Juice (2)	0.00	-3.38 to 3.38	
	Cherry Juice (2)	7.41	-13.17 to 27.99	
	Grapefruit Juice (1)	6.90	2.98 to 10.82	
	Bayberry Juice (1)	4.10	-2.76 to 10.96	
	Strawberry Juice (1)	3.90	-9.04 to 16.84	
	Blueberry Juice (1)	0.00	-2.27 to 2.27	
	Chokeberry Juice (1)	-1.16	-2.67 to 0.35	
	Strawberry+Cranberry Juice (1)	-1.16	-10.02 to 7.70	
	Mix Juice (1)	0.20	-8.42 to 8.82	
**Comparator**	Placebo (20)	-0.61	-1.72 to 0.50	0.003
	Water (4)	3.04	-1.64 to 7.73	
	Control drink (1)	17.80	4.94 to 30.66	
**Fruit Juice energy content compared to control drink**	Iso-caloric (18)	-0.49	-1.61 to 0.64	0.14
	Hyper-caloric (6)	2.65	-1.19 to 6.49	

	Unspecified (1)	0.00	-8.33 to 8.33	
**Diet during trial**	Usual (15)	-0.48	-1.54 to 0.59	0.003
	Without confounder sources (8)	0.05	-2.77 to 2.87	
	Controlled diet (1)	3.86	-3.71 to 11.43	
	Restricted energy intake (1)	6.90	2.98 to 10.82	
**Country of origin**	Noth America (13)	1.18	-0.47 to 2.83	0.22
	Europe (9)	-0.14	-2.12 to 1.85	
	East Asia (1)	4.10	-2.76 to 10.96	
	Asia-Middle East (2)	-0.60	-3.64 to 2.44	
**Reconstituited Fruit Juice**	Yes (11)	-0.65	-1.82 to 0.51	0.03
	No (15)	1.66	-0.02 to 3.34	

F. **HDL-cholesterol**		
**Variables** (n. of cohorts)	**Coefficient**	**95% CI**
**Age (years) (25)**	-.1863635	-.3087853 -.0639417
**Length of intervention (week) (25)**	.2413576	-.2023281 .6850434
**Number of participants (n) (25)**	.1255153	-.0099144 .260945
**Gender (% men) (23)**	-.0196734	-.070756 .0314092
**Year of publication (year) (25)**	-.2164203	-.6771233 .2442827
**Volume of Fruit Juice (ml/day) (25)**	-.006098	-.0161544 .0039584
**Energy intake by Fruit Juice (Kcal/day) (13)**	-.0059715	-.0414075 .0294645
**Baseline BMI (kg/m^2) (23)**	.0392619	-.3277629 .4062867
**Baseline total cholesterol (mg/dl) (23)**	-.0138991	-.0650726 .0372744
**Baseline LDL-cholesterol (mg/dl) (20)**	-.0128271	-.0841724 .0585182
**Baseline HDL-cholesterol (mg/dl) (23)**	-.0727696	-.1925036 .0469644
**Baseline triglycerides (mg/dl) (22)**	.0001661	-.0193737 .0197059
**Risk of bias (point) (25)**	.2681949	-1.17805 1.71444

G. Triglycerides

	Variables (n. of cohorts)	Mean Difference (mg/dl)	95% CI	P for interaction
**Study design**	Cross-over (8)	-3.75	-11.14 to 3.64	0.25
	Parallel-arm (18)	2.33	-4.93 to 9.59	
**Type of blinding**	Double-blind (14)	3.44	-5.01 to 11.89	0.26
	Single-blind (3)	-6.05	-14.42 to 2.31	
	Open-label (8)	1.55	-9.00 to 12.09	
**Risk factors**	Healthy (5)	-2.55	-17.11 to 12.01	0.95
	Overweight/obese (5)	-3.43	-16.56 to 9.70	
	With cardio-metabolic risk factors (15)	0.28	-5.84 to 6.40	
	Other disease (1)	2.50	-161.35 to 166.35	
**Type of Juice**	Pomegranate Juice (6)	5.38	-19.13 to 29.90	0.92
	Cranberry Juice (5)	6.23	-4.62 to 17.07	
	Grape Juice (4)	1.49	-18.19 to 21.17	
	Orange Juice (2)	-23.12	-72.06 to 25.82	
	Cherry Juice (1)	2.80	-22.09 to 27.69	
	Grapefruit Juice (1)	-5.10	-20.70 to 10.50	
	Bayberry Juice (1)	-5.00	-38.91 to 28.91	
	Strawberry Juice (1)	-8.85	-163.29 to 145.59	
	Blueberry Juice (1)	-8.85	-47.07 to 29.37	
	Bilberry Juice (1)	11.50	-9.31 to 32.31	
	Chokeberry Juice (1)	-5.31	-13.97 to 3.35	
	Strawberry+Cranberry Juice (1)	-3.54	-85.07 to 77.99	
	Mix Juice (1)	-8.40	-39.76 to 22.96	
**Comparator**	Placebo (21)	-0.87	-6.64 to 4.90	0.87
	Water (5)	0.25	-11.51 to 12.01	

**Fruit Juice energy content compared to control drink**	Iso-caloric (18)	-1.31	-7.16 to 4.55	0.89
	Hyper-caloric (7)	1.76	-9.57 to 13.08	
	Unspecified (1)	0.00	-57.23 to 57.23	
**Diet during trial**	Usual (14)	-3.55	-10.83 to 3.74	0.36
	Without confounder sources (10)	5.59	-3.13 to 14.30	
	Controlled diet (1)	-8.85	-38.25 to 20.55	
	Restricted energy intake (1)	-5.10	-20.70 to 10.50	
**Country of origin**	Noth America (13)	1.52	-6.30 to 9.33	0.90
	Europe (10)	-2.33	-9.47 to 4.81	
	East Asia (1)	-5.00	-38.91 to 28.91	
	Asia-Middle East (2)	1.95	-46.70 to 50.61	
**Reconstituited Fruit Juice**	Yes (11)	-0.15	-6.34 to 6.04	0.77
	No (15)	-1.84	-11.30 to 7.61	

H. **Triglycerides**			
**Variables** (n. of cohorts)	**Coefficient**	**95% CI**	
**Age (years) (26)**	.0686716	-.4432899	.5806331
**Length of intervention (week) (26)**	-1.053352	-3.064975	.9582709
**Number of participants (n) (26)**	-.1542805	-.6537077	.3451467
**Gender (% men) (24)**	-.0867644	-.3138965	.1403678
**Year of publication (year) (26)**	-1.649902	-3.507787	.2079838
**Volume of Fruit Juice (ml/day) (26)**	.0202564	-.01706	.0575728
**Energy intake by Fruit Juice (Kcal/day) (13)**	.0167375	-.0789498	.1124249
**Baseline BMI (kg/m^2) (24)**	.5578336	-.4188746	1.534542
**Baseline total cholesterol (mg/dl) (24)**	.0762774	-.2006492	.3532041
**Baseline LDL-cholesterol (mg/dl) (21)**	.0601829	-.3564828	.4768487
**Baseline HDL-cholesterol (mg/dl) (23)**	-.4025435	-1.113005	.3079182
**Baseline triglycerides (mg/dl) (24)**	.1124303	-.0965081	.3213688
**Risk of bias (point) (26)**	-2.099691	-9.09653	4.897149

Supplemental Table 11. Subgroup analysis and meta-regression analysis of the effect of fruit juice consumption on glucose metabolism

A. **Glucose**

	Variables (n. of cohorts)	Mean Difference (mg/dl)	95% CI	P for interaction
**Study design**	Cross-over (9)	-0.64	-2.44 to 1.15	0.20
	Parallel-arm (14)	-0.64	-6.27 to 5.00	
**Type of blinding**	Double-blind (15)	-1.57	-6.53 to 3.40	0.01
	Single-blind (3)	0.40	-1.61 to 2.40	
	Open-label (5)	0.50	-2.64 to 3.64	
**Risk factors**	Healthy (3)	-2.23	-6.76 to 2.30	0.44
	Overweight/obese (5)	0.83	-2.16 to 3.83	
	With cardio-metabolic risk factors (14)	-1.29	-6.41 to 3.83	
	Other disease (1)	3.60	-45.01 to 52.21	
**Type of Juice**	Pomegranate Juice (7)	-0.27	-4.70 to 4.16	0.001
	Cranberry Juice (4)	-6.81	-19.96 to 6.35	
	Grape Juice (4)	-0.04	-4.54 to 4.46	
	Orange Juice (2)	-1.04	-8.73 to 6.65	
	Cherry Juice (1)	6.40	-1.79 to 14.59	
	Grapefruit Juice (1)	1.80	-2.08 to 5.68	
	Bayberry Juice (1)	-5.41	-10.70 to -0.12	
	Strawberry Juice (1)	1.80	-11.98 to 15.58	
	Blueberry Juice (1)	3.60	-6.65 to 13.85	
	Chokeberry Juice (1)	0.36	-1.76 to 2.48	
**Comparator**	Placebo (19)	-1.63	-5.16 to 1.89	0.05
	Water (4)	2.09	-1.40 to 5.57	
**Fruit Juice energy content**	Iso-caloric (15)	-0.49	-2.25 to 1.26	0.06

**compared to control drink**	Hyper-caloric (7)	-2.34	-12.50 to 7.81	
	Unspecified (1)	1.80	-8.80 to 12.40	
**Diet during trial**	Usual (14)	-0.90	-5.93 to 4.12	0.39
	Without confounder sources (7)	-1.69	-4.99 to 1.60	
	Controlled diet (1)	-2.88	-7.37 to 1.61	
	Restricted energy intake (1)	1.80	-2.08 to 5.68	
**Country of origin**	Noth America (12)	0.45	-1.71 to 2.60	0.001
	Europe (7)	0.19	-1.69 to 2.06	
	East Asia (1)	-5.41	-10.70 to -0.12	
	Asia-Middle East (3)	-8.17	-29.15 to 12.80	
**Reconstituited Fruit Juice**	Yes (7)	1.00	-0.94 to 2.93	0.002
	No (16)	-2.23	-6.34 to 1.88	

**B. Glucose**		
**Variables** (n. of cohorts)	**Coefficient**	**95% CI**
**Age (years) (23)**	-.0519172	-.305844 .2020095
**Length of intervention (week) (23)**	-.3375352	-1.180918 .5058476
**Number of participants (n) (23)**	-.1732439	-.4049484 .0584606
**Gender (% men) (21)**	-.1983727	-.3003487 -.0963967
**Year of publication (year) (23)**	.5575233	-.9171019 2.032149
**Volume of Fruit Juice (ml/day) (23)**	.0099321	-.0152675 .0351318
**Energy intake by Fruit Juice (Kcal/day) (14)**	-.0043021	-.029963 .0213588
**Baseline BMI (kg/m^2) (21)**	.3435247	-.4422309 1.12928
**Baseline glucose (mg/dl) (21)**	-.1608723	-.3420349 .0202903
**Baseline HOMA index (Unit) (8)**	2.66177	-1.184907 6.508448
**Baseline Insulin (mIU/L) (10)**	.3039095	-.4607158 1.068535
**Risk of bias (point) (23)**	-1.971158	-5.547064 1.604748

C. HOMA index				
	**Variables (n. of cohorts)**	**Mean Difference (Unit)**	**95% CI**	**P for interaction**
**Study design**	Cross-over (5)	-0.18	-0.63 to 0.27	0.28
	Parallel-arm (6)	0.14	-0.23 to 0.52	
**Type of blinding**	Double-blind (7)	-0.10	-0.51 to 0.31	0.73
	Single-blind (2)	0.27	-0.89 to 1.43	
	Open-label (2)	0.06	-0.80 to 0.93	
**Risk factors**	Healthy (2)	-0.03	-0.50 to 0.43	0.95
	Overweight/obese (2)	0.06	-0.80 to 0.93	
	With cardio-metabolic risk factors (7)	-0.01	-0.56 to 0.54	
**Type of Juice**	Pomegranate Juice (3)	-0.27	-0.94 to 0.39	0.45
	Cranberry Juice (2)	-0.15	-0.72 to 0.43	
	Grape Juice (1)	-0.50	-1.68 to 0.68	
	Orange Juice (1)	-0.29	-1.25 to 0.67	
	Cherry Juice (1)	-0.50	-2.62 to 1.62	
	Grapefruit Juice (1)	0.50	-0.19 to 1.19	
	Bayberry Juice (1)	0.10	-0.64 to 0.84	
	Blueberry Juice (1)	0.90	-0.22 to 2.02	
**Comparator**	Placebo (9)	-0.18	-0.52 to 0.15	0.02
	Water (2)	0.61	0.02 to 1.19	
**Fruit Juice energy content compared to control drink**	Iso-caloric (9)	-0.18	-0.52 to 0.15	0.02
	Hyper-caloric (2)	0.61	0.02 to 1.19	
**Diet during trial**	Usual (6)	-0.08	-0.55 to 0.40	0.49
	Without confounder sources (3)	-0.10	-0.71 to 0.50	
	Controlled diet (1)	-0.12	-0.72 to 0.48	
	Restricted energy intake (1)	0.50	-0.19 to 1.19	
**Country of origin**	Noth America (6)	0.13	-0.28 to 0.54	0.53
	Europe (2)	-0.35	-0.92 to 0.22	
	East Asia (1)	0.10	-0.64 to 0.84	
	Asia-Middle East (2)	0.52	-1.42 to 2.46	

**Reconstituited Fruit Juice**	Yes (3)	0.38	-0.56 to 1.33	0.36
	No (8)	-0.04	-0.34 to 0.27	

D. **HOMA index**		
**Variables** (n. of cohorts)	**Coefficient**	**95% CI**
**Age (years) (11)**	-.0115631	-.0416732 .018547
**Length of intervention (week) (11)**	.0327783	-.0682094 .133766
**Number of participants (n) (11)**	.0114502	-.017769 .0406694
**Gender (% men) (11)**	-.012389	-.0262751 .0014971
**Year of publication (year) (11)**	-.0637805	-.221108 .0935471
**Volume of Fruit Juice (ml/day) (11)**	-.0012117	-.0034442 .0010208
**Energy intake by Fruit Juice (Kcal/day) (8)**	-.0016061	-.007103 .0038908
**Baseline BMI (kg/m^2) (10)**	.0811181	-.0061392 .1683755
**Baseline glucose (mg/dl) (8)**	.0051798	-.0303702 .0407297
**Baseline HOMA index (Unit) (9)**	.0701068	-.53783 .6780436
**Baseline Insulin (mIU/L) (7)**	.035834	-.142069 .213737
**Risk of bias (point) (11)**	-.0775085	-.3540795 .1990624

E. Insulin				
	**Variables (n. of cohorts)**	**Mean Difference (%)**	**95% CI**	**P for interaction**
**Study design**	Cross-over (6)	7.04	-8.12 to 22.20	0.51
	Parallel-arm (5)	-0.03	-14.80 to 14.73	
**Type of blinding**	Double-blind (8)	5.08	-7.78 to 17.93	0.65
	Open-label (3)	-0.08	-18.70 to 18.54	
**Risk factors**	Healthy (2)	2.46	-21.42 to 26.33	0.98
	Overweight/obese (4)	2.33	-15.58 to 20.24	
	With cardio-metabolic risk factors (5)	4.65	-11.03 to 20.33	
**Type of Juice**	Pomegranate Juice (3)	13.94	-4.37 to 32.26	0.77
	Cranberry Juice (2)	-5.23	-32.21 to 21.74	
	Grape Juice (2)	-11.46	-44.52 to 21.59	
	Orange Juice (1)	-12.66	-106.68 to 81.36	
	Cherry Juice (1)	-21.20	-79.61 to 37.21	
	Grapefruit Juice (1)	3.00	-17.29 to 23.29	
	Bayberry Juice (1)	8.40	-26.27 to 43.07	
**Comparator**	Placebo (10)	3.57	-8.83 to 15.96	0.96
	Water (1)	3.00	-17.29 to 23.29	
**Fruit Juice energy content compared to control drink**	Iso-caloric (9)	2.50	-10.12 to 15.13	0.68
	Hyper-caloric (1)	3.00	-17.29 to 23.29	
	Unspecified (1)	32.30	-33.36 to 97.96	
**Diet during trial**	Usual (5)	7.36	-8.49 to 23.20	0.90
	Without confounder sources (4)	-2.14	-27.12 to 22.85	
	Controlled diet (1)	-2.90	-35.83 to 30.03	
	Restricted energy intake (1)	3.00	-17.29 to 23.29	
**Country of origin**	Noth America (7)	-1.74	-15.55 to 12.07	0.40
	Europe (2)	-16.52	-63.41 to 30.37	
	East Asia (1)	8.40	-26.27 to 43.07	
	Asia-Middle East (1)	16.68	-3.70 to 37.06	
**Reconstituited Fruit Juice**	Yes (3)	-14.18	-48.31 to 19.96	0.29

	No (8)	5.28	-5.84 to 16.41

F. Insulin

**Variables** (n. of cohorts)	**Coefficient**	**95% CI**	
**Age (years) (11)**	-.3330365	-1.209831	.5437584
**Length of intervention (week) (11)**	-1.328579	-3.729908	1.07275
**Number of participants (n) (11)**	-.2156451	-1.146235	.7149452
**Gender (% men) (10)**	-.2363909	-1.002917	.5301354
**Year of publication (year) (11)**	2.288499	-2.478942	7.055939
**Volume of Fruit Juice (ml/day) (11)**	-.0096196	-.082626	.0633869
**Energy intake by Fruit Juice (Kcal/day) (7)**	-.0407179	-.2900348	.2085989
**Baseline BMI (kg/m^2) (9)**	.9989227	-2.865285	4.863131
**Baseline glucose (mg/dl) (10)**	.304036	-.2717005	.8797724
**Baseline HOMA index (Unit) (7)**	9.544391	-12.17246	31.26124
**Baseline Insulin (mIU/L) (10)**	.0520487	-3.902234	4.006332
**Risk of bias (point) (11)**	-1.408192	-19.86313	17.04675
