# Supplementary material for: 100% Fruit juice intake and cardiovascular risk: a systematic review and meta-analysis of prospective and randomised controlled studies
Source: Eur J Nutr. 2020 Nov 4;60(5):2449–67. doi: 10.1007/s00394-020-02426-7 (PMC8275541; doi:10.1007/s00394-020-02426-7)

Titles and Legends to Supplemental Figures.

Supplemental Figure 1. Stepwise procedure for selection of the studies.
Flowchart indicating the results of the systematic review with inclusions and exclusions.

Supplemental Figure 2. Dose-response association between fruit drink consumption and risk of stroke. Fruit drink consumption was modelled with restricted cubic splines in a multivariate random-effects dose–response model (solid line). Dashed lines represent the 95% confidence intervals for the spline model.

Supplemental Figure 3. Dose-response association between fruit drink consumption and risk of coronary heart disease (CHD). Fruit drink consumption was modelled with restricted cubic splines in a multivariate random-effects dose–response model (solid line). Dashed lines represent the 95% confidence intervals for the spline model.

Supplemental Figure 4. Dose-response association between 100% citrus juice consumption and risk of diabetes. 100% citrus juice consumption was modelled with restricted cubic splines in a multivariate random-effects dose–response model (solid line). Dashed lines represent the 95% confidence intervals for the spline model.

Supplemental Figure 5. Dose-response association between fruit drink consumption and risk of diabetes. Fruit drink consumption was modelled with restricted cubic splines in a multivariate random-effects dose–response model (solid line). Dashed lines represent the 95% confidence intervals for the spline model.

Supplemental Figure 6. Funnel plot of the effect on systolic blood pressure (SBP) changes.

Supplemental Figure 7. Funnel plot of the effect on diastolic blood pressure (DBP) changes.

Supplemental Figure 8. Funnel plot of the effect on weight changes.

Supplemental Figure 9. Funnel plot of the effect on BMI changes.

Supplemental Figure 10. Funnel plot of the effect on waist circumference changes.

Supplemental Figure 11. Forest plot of the effect of 100% fruit juice intake on total cholesterol.
Results are expressed as Mean Difference (MD) and 95% confidence intervals (95% CI). Squares indicate study-specific relative risk estimates (size of the square reflects the study-specific statistical weight); horizontal lines indicate 95% CI; diamond indicates the overall relative risk with its 95% CI.

Supplemental Figure 12. Forest plot of the effect of 100% fruit juice intake on LDL-Cholesterol.
Results are expressed as Mean Difference (MD) and 95% confidence intervals (95% CI). Squares indicate study-specific relative risk estimates (size of the square reflects the study-specific statistical weight); horizontal lines indicate 95% CI; diamond indicates the overall relative risk with its 95% CI.

Supplemental Figure 13. Forest plot of the effect of 100% fruit juice intake on HDL-cholesterol.
Results are expressed as Mean Difference (MD) and 95% confidence intervals (95% CI). Squares indicate study-specific relative risk estimates (size of the square reflects the study-specific statistical weight); horizontal lines indicate 95% CI; diamond indicates the overall relative risk with its 95% CI.

Supplemental Figure 14. Forest plot of the effect of 100% fruit juice intake on triglycerides.

Results are expressed as Mean Difference (MD) and 95% confidence intervals (95% CI). Squares indicate study-specific relative risk estimates (size of the square reflects the study-specific statistical weight); horizontal lines indicate 95% CI; diamond indicates the overall relative risk with its 95% CI.

Supplemental Figure 15. Funnel plot of the effect on total cholesterol changes.

Supplemental Figure 16. Funnel plot of the effect on LDL-cholesterol changes.

Supplemental Figure 17. Funnel plot of the effect on HDL-cholesterol changes.

Supplemental Figure 18. Funnel plot of the effect on triglycerides changes.

Supplemental Figure 19. Forest plot of the effect of 100% fruit juice intake on serum glucose.
Results are expressed as Mean Difference (MD) and 95% confidence intervals (95% CI). Squares indicate study-specific relative risk estimates (size of the square reflects the study-specific statistical weight); horizontal lines indicate 95% CI; diamond indicates the overall relative risk with its 95% CI.

Supplemental Figure 20. Forest plot of the effect of 100% fruit juice intake on HOMA index.
Results are expressed as Mean Difference (MD) and 95% confidence intervals (95% CI). Squares indicate study-specific relative risk estimates (size of the square reflects the study-specific statistical weight); horizontal lines indicate 95% CI; diamond indicates the overall relative risk with its 95% CI.

Supplemental Figure 21. Forest plot of the effect of 100% fruit juice intake on serum insulin.
Results are expressed as Mean Difference (MD) and 95% confidence intervals (95% CI). Squares indicate study-specific relative risk estimates (size of the square reflects the study-specific statistical weight); horizontal lines indicate 95% CI; diamond indicates the overall relative risk with its 95% CI.

Supplemental Figure 22. Forest plot of the effect of 100% fruit juice intake on glycated haemoglobin concentration.
Results are expressed as Mean Difference (MD) and 95% confidence intervals (95% CI). Squares indicate study-specific relative risk estimates (size of the square reflects the study-specific statistical weight); horizontal lines indicate 95% CI; diamond indicates the overall relative risk with its 95% CI.

Supplemental Figure 23. Funnel plot of the effect on serum glucose changes.

Supplemental Figure 24. Funnel plot of the effect on HOMA index changes.

Supplemental Figure 25. Funnel plot of the effect on serum insulin changes.

Supplemental Figure 26. Forest plot of the effect of 100% fruit juice intake on pulse wave velocity (PWV).
Results are expressed as Mean Difference (MD) and 95% confidence intervals (95% CI). Squares indicate study-specific relative risk estimates (size of the square reflects the study-specific statistical weight); horizontal lines indicate 95% CI; diamond indicates the overall relative risk with its 95% CI.

Supplemental Figure 27. Funnel plot of the effect on pulse wave velocity (PWV) changes.

Supplemental Figure 28. Forest plot of the effect of 100% fruit juice intake on flow-mediated dilation (FMD).

Results are expressed as Mean Difference (MD) and 95% confidence intervals (95% CI). Squares indicate study-specific relative risk estimates (size of the square reflects the study-specific statistical weight); horizontal lines indicate 95% CI; diamond indicates the overall relative risk with its 95% CI.

Supplemental Figure 29. Funnel plot of the effect on flow-mediated dilation (FMD) changes.

Supplemental Figure 1

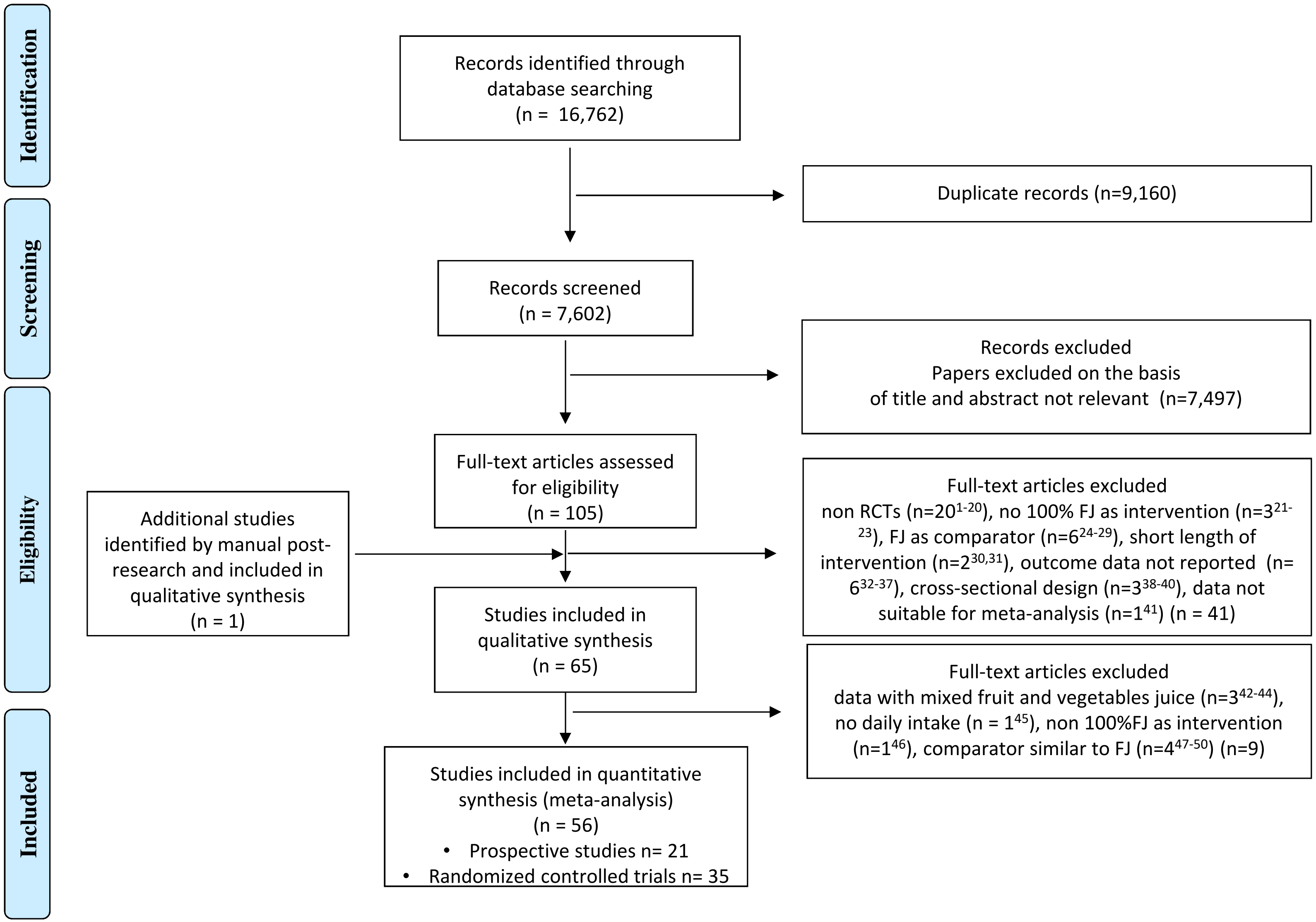

Supplemental Figure 2

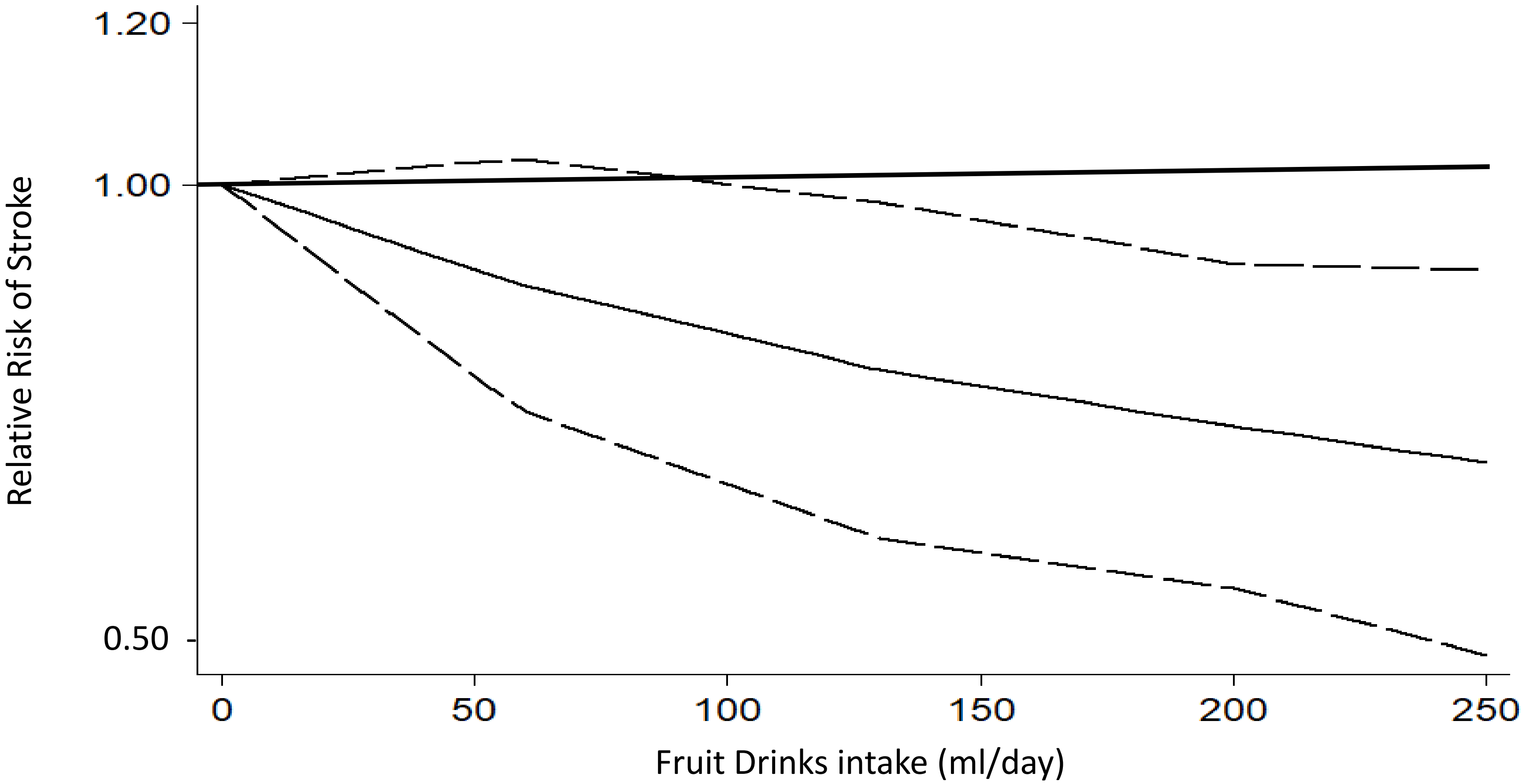

Supplemental Figure 3

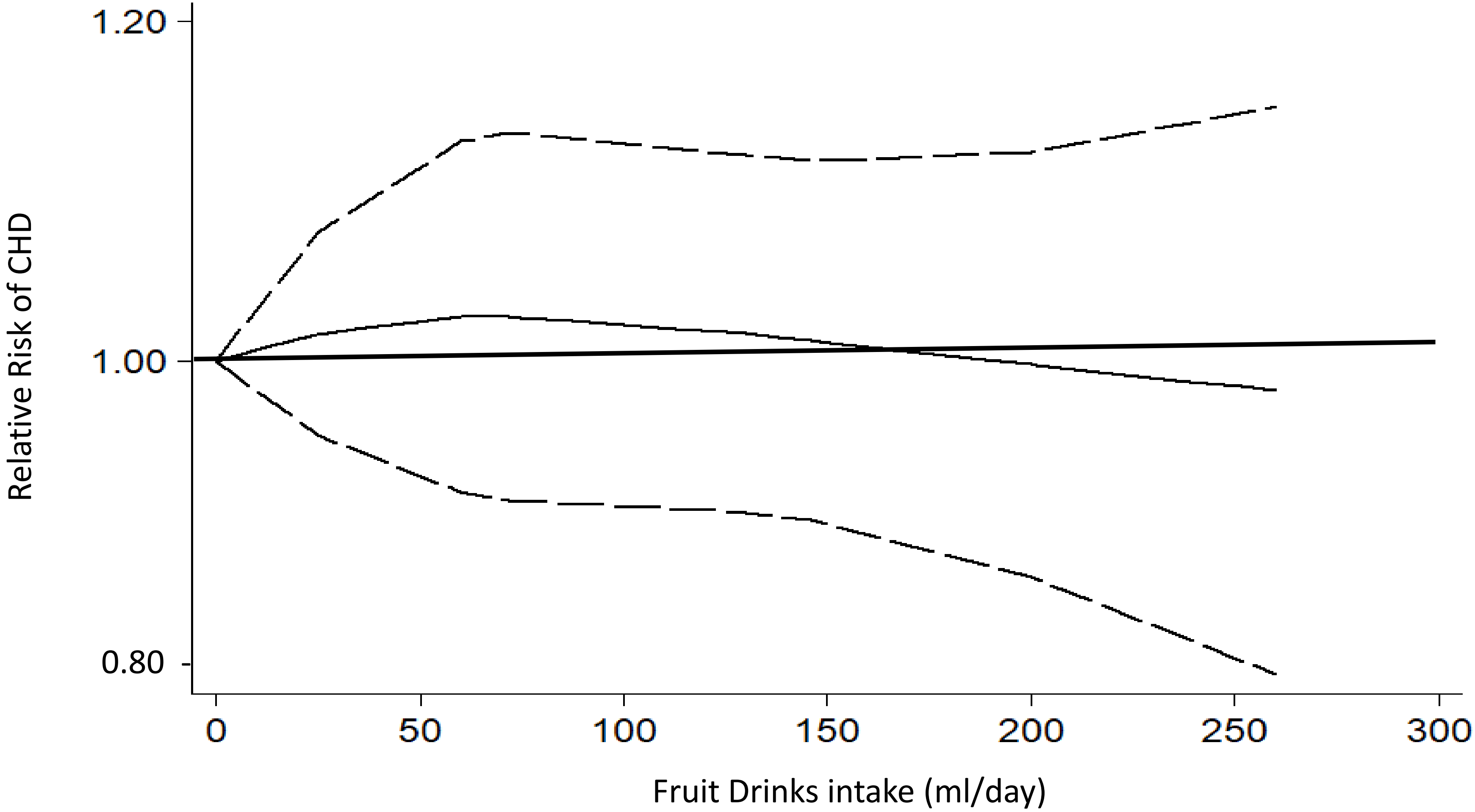

Supplemental Figure 4

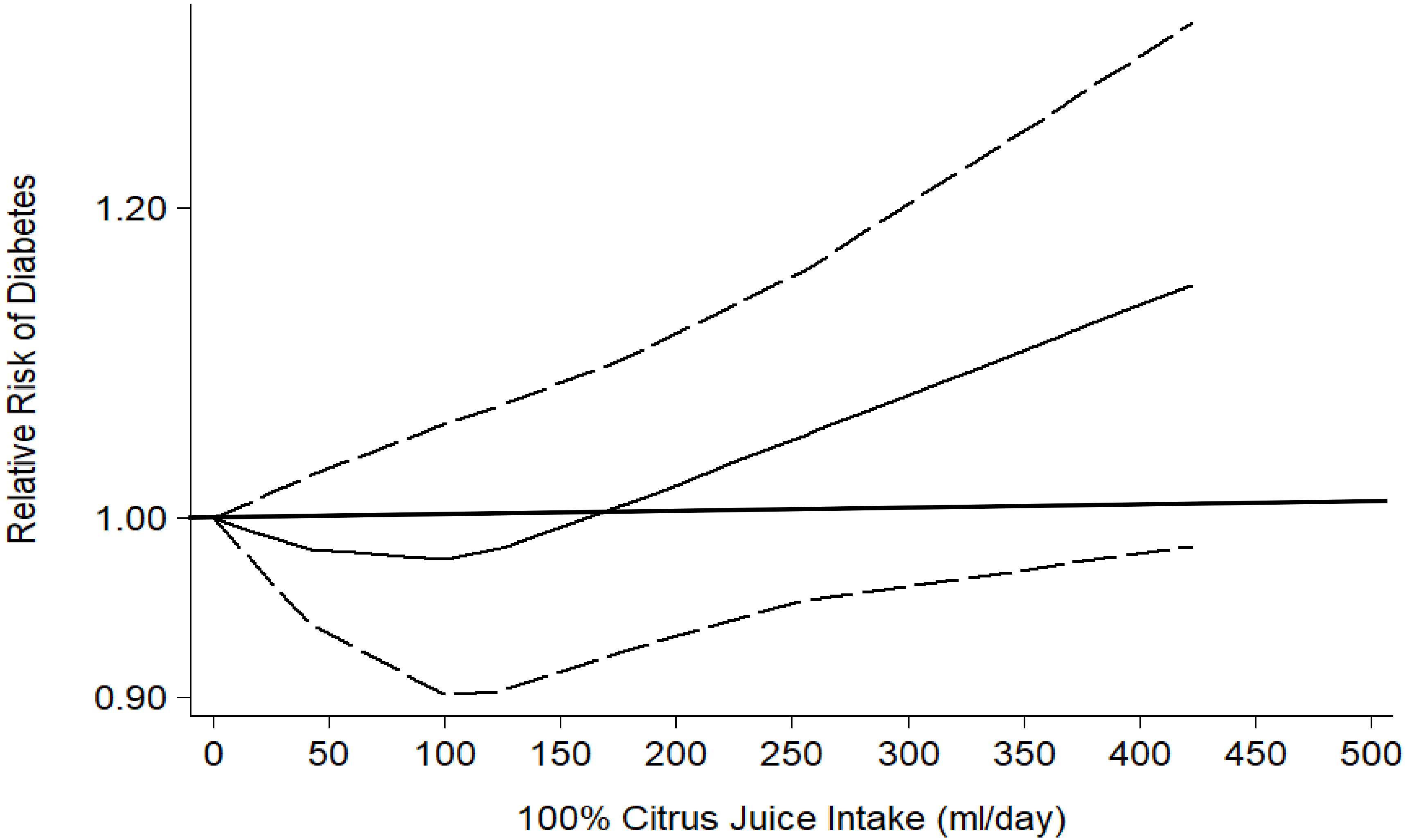

Supplemental Figure 5

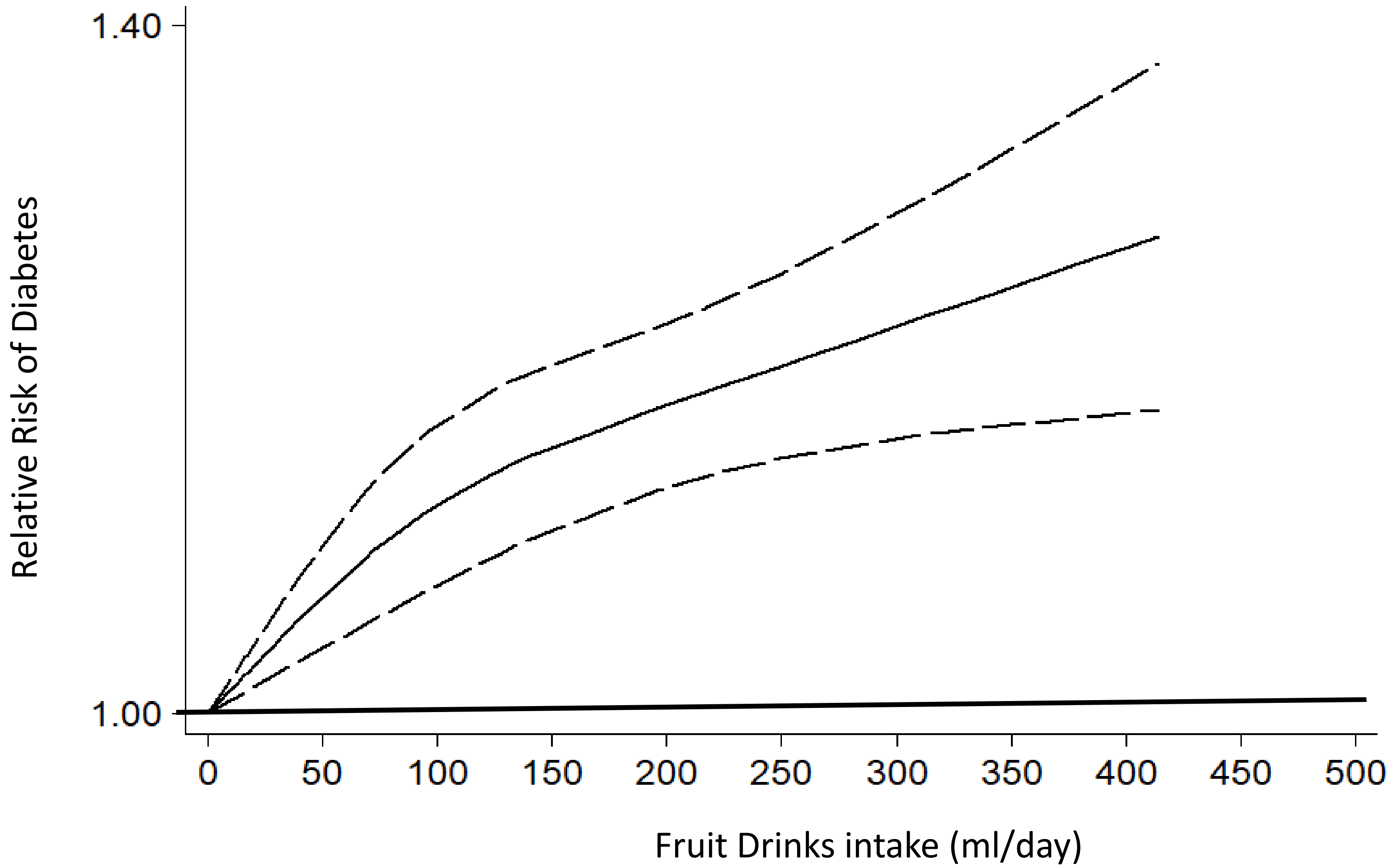

Supplemental Figure 6

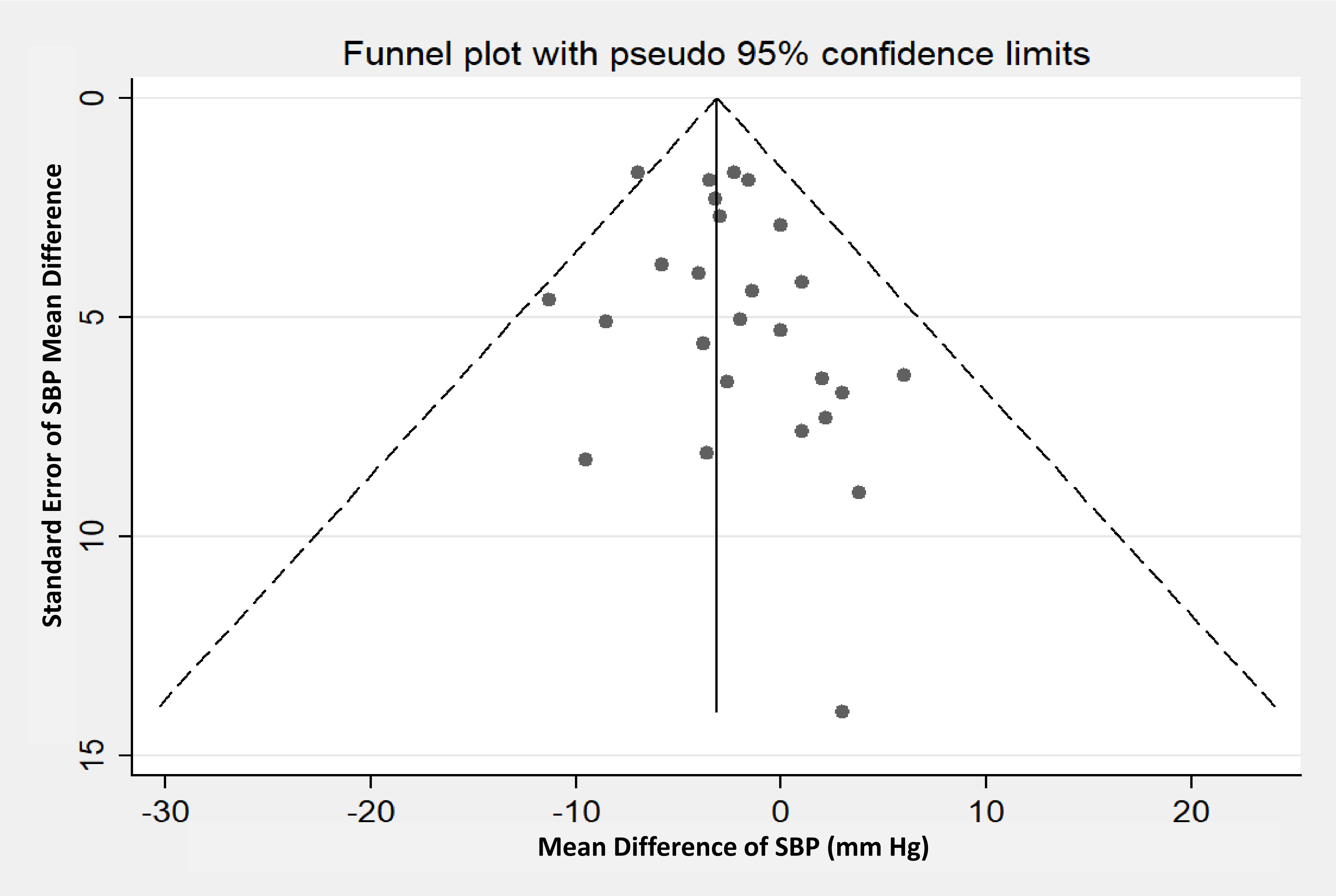

Supplemental Figure 7

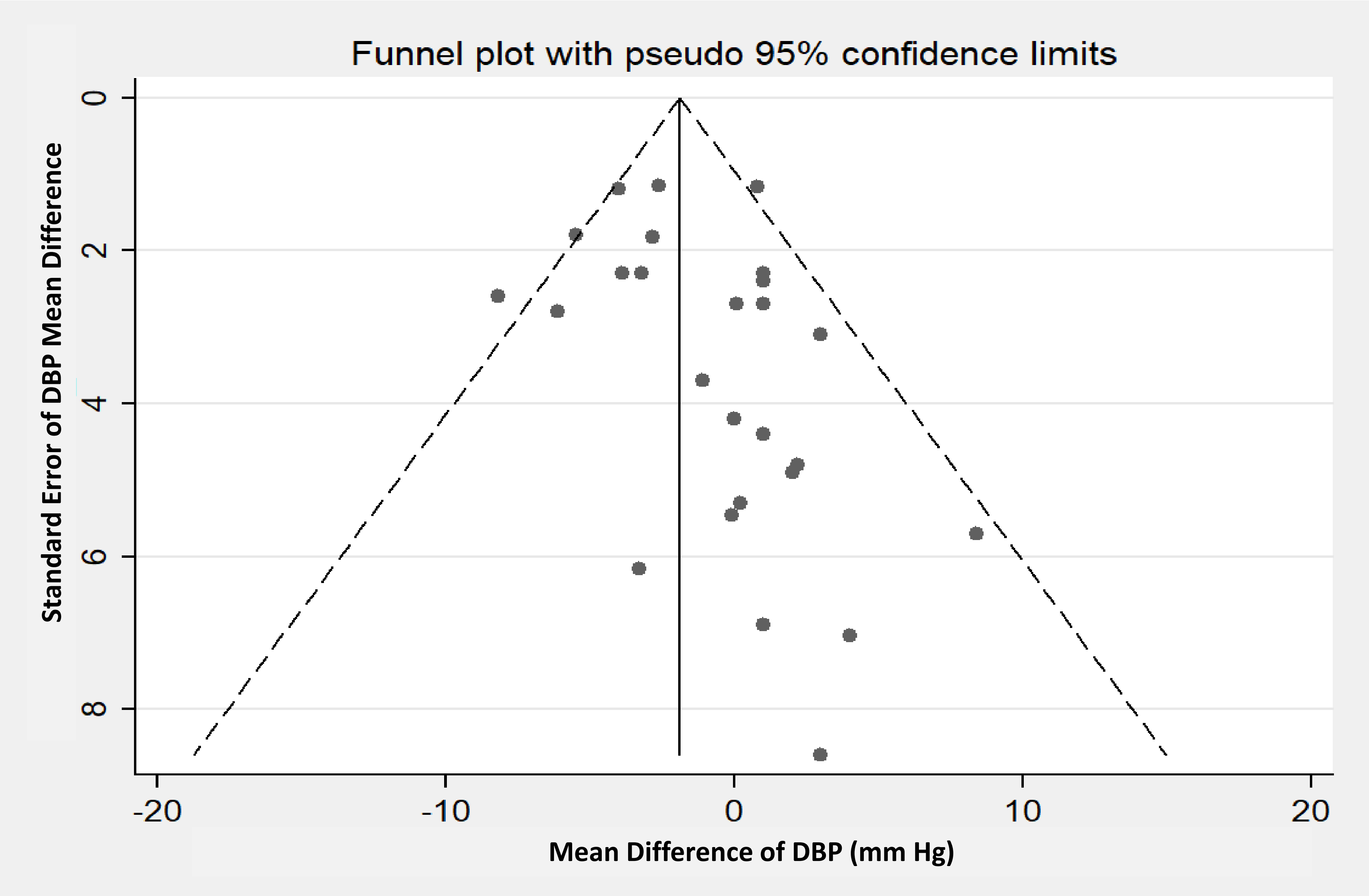

Supplemental Figure 8

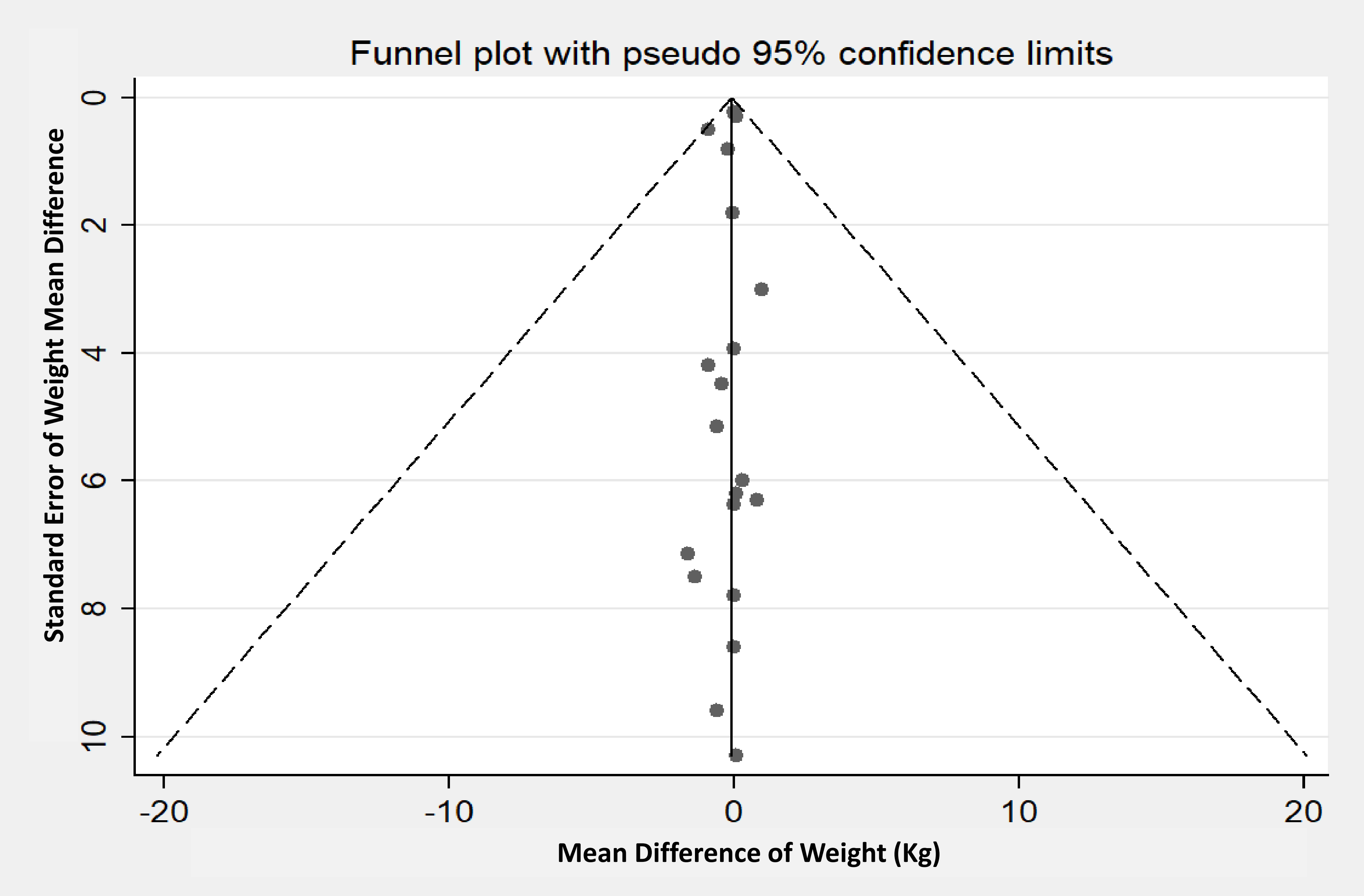

Supplemental Figure 9

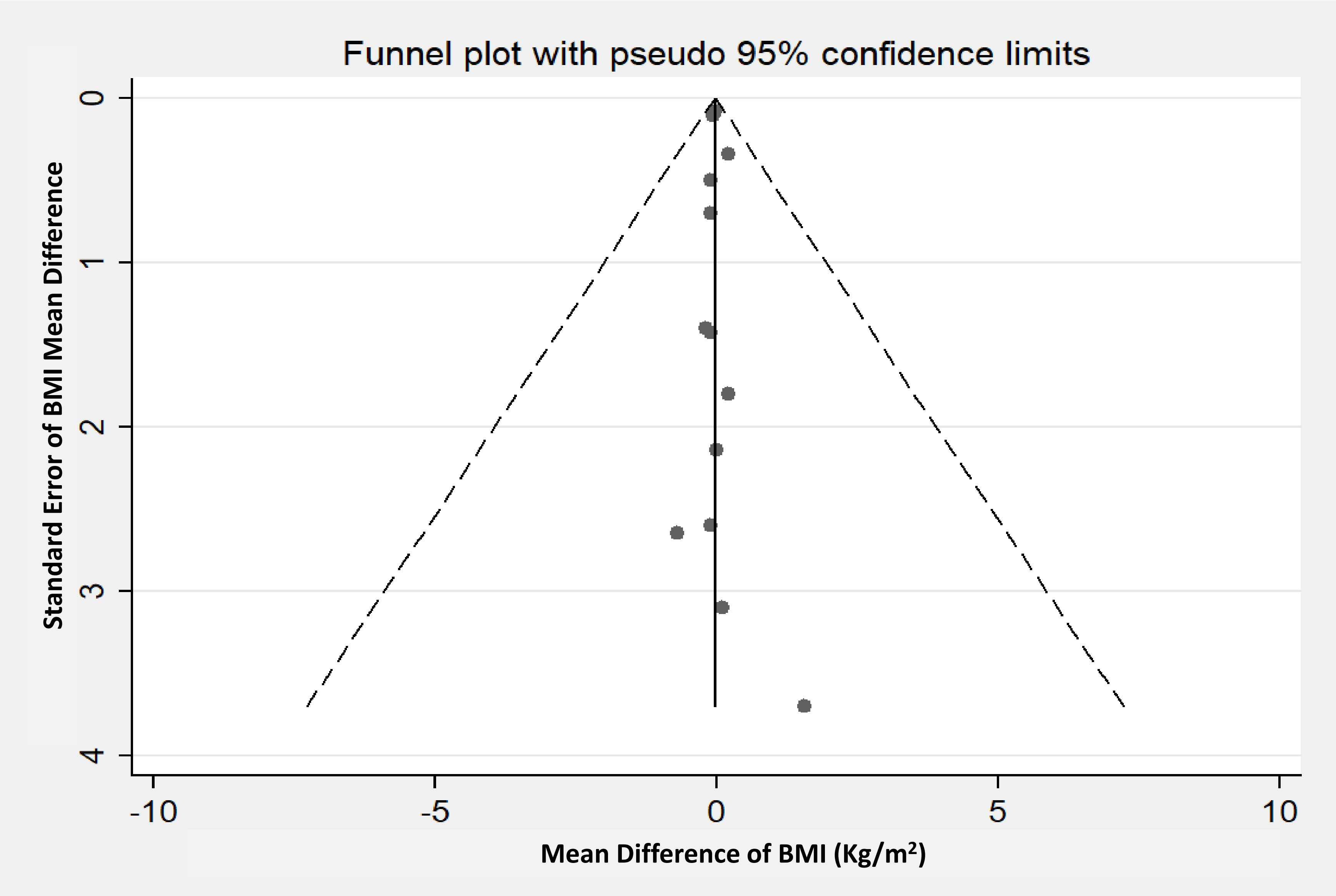

Supplemental Figure 10

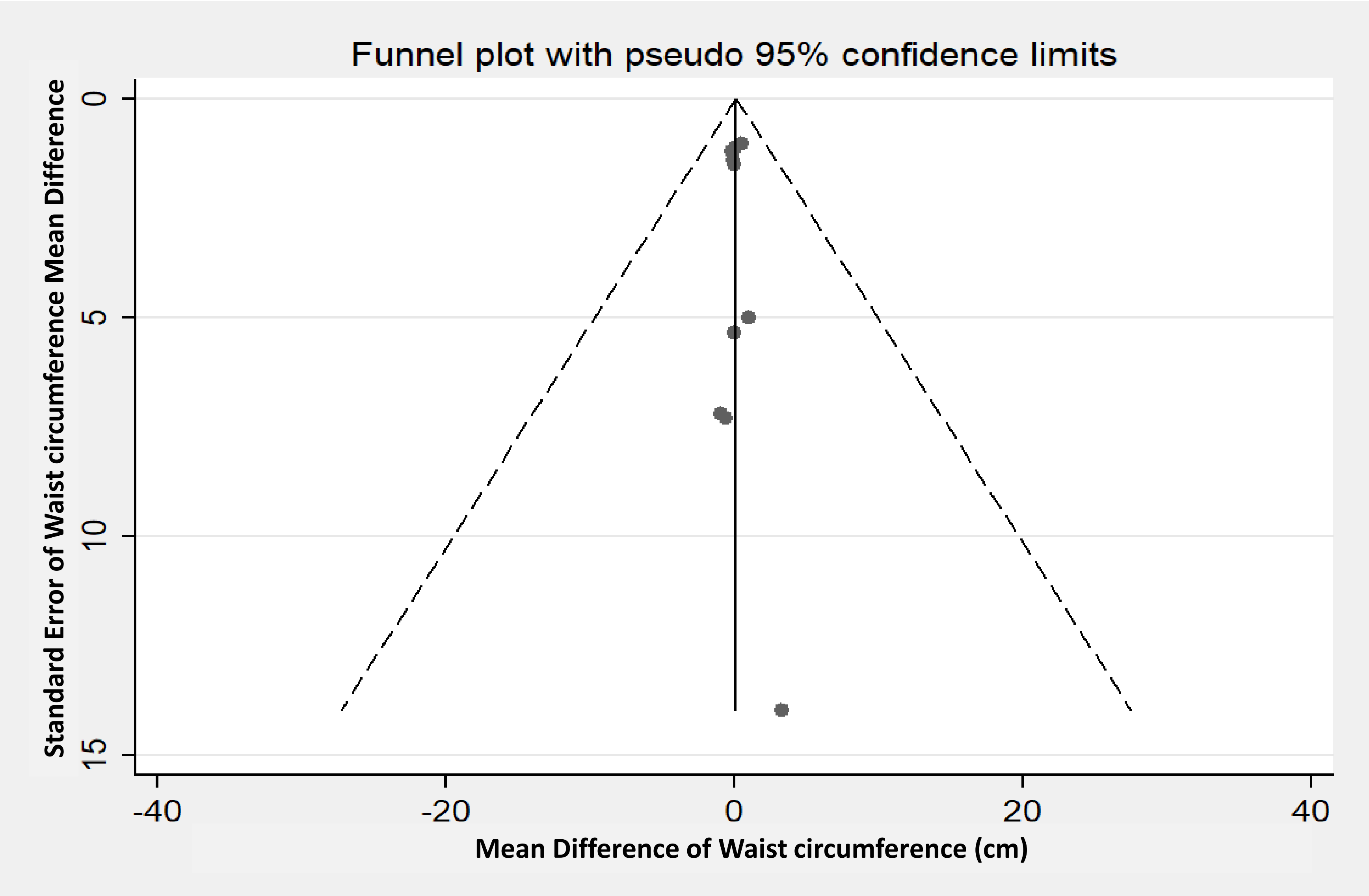

Supplemental Figure 11

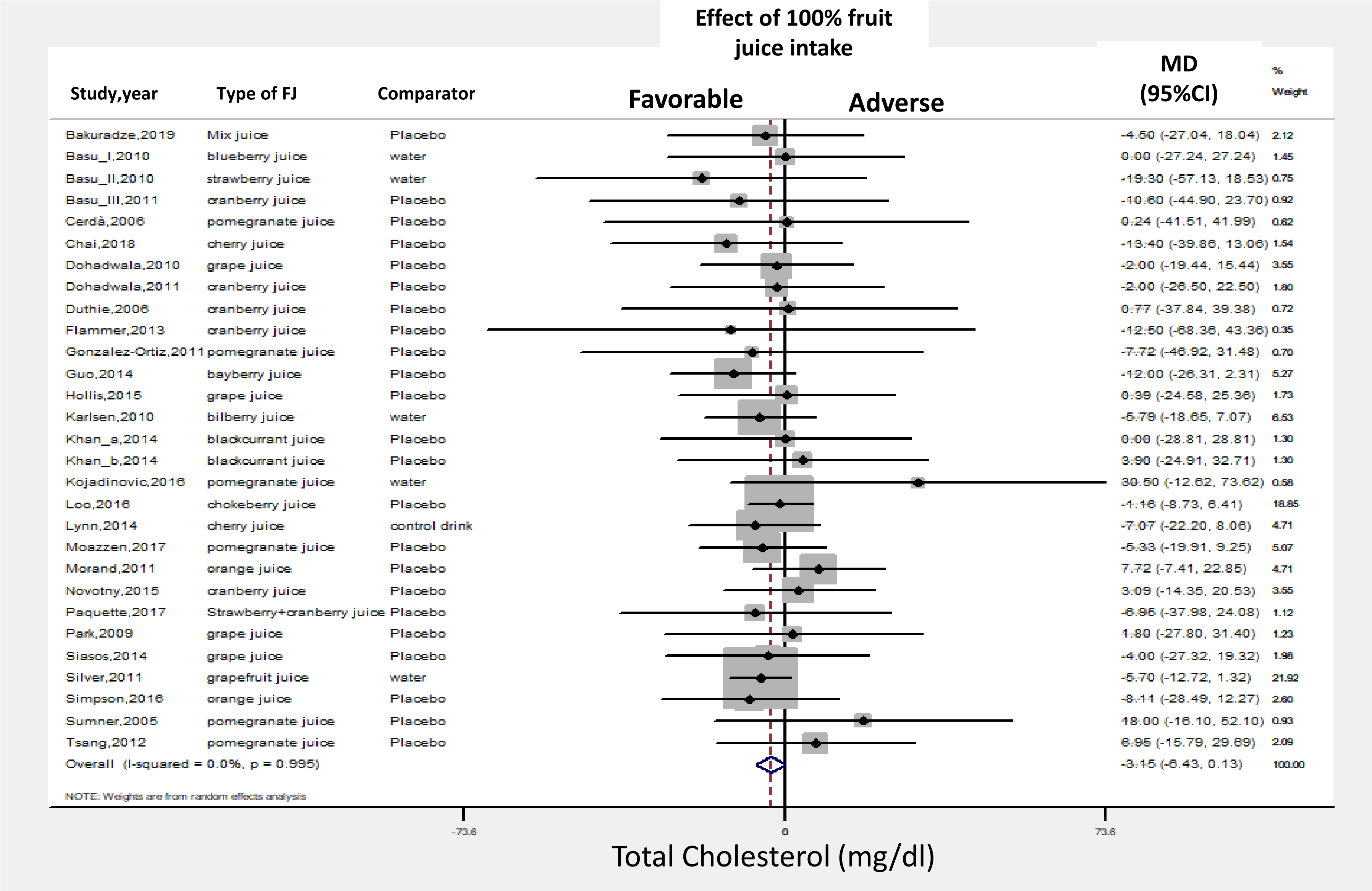

Supplemental Figure 12

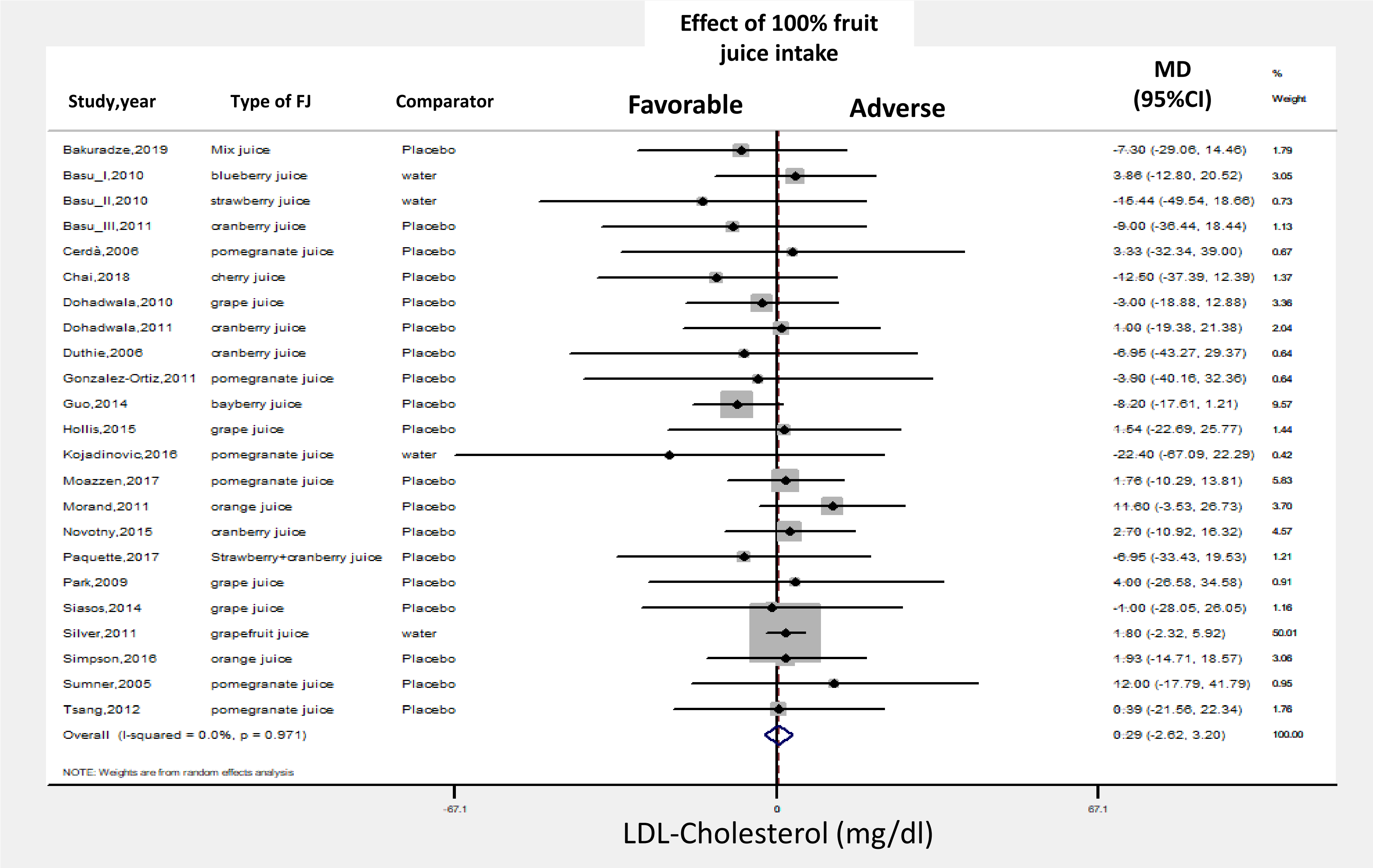

Supplemental Figure 13

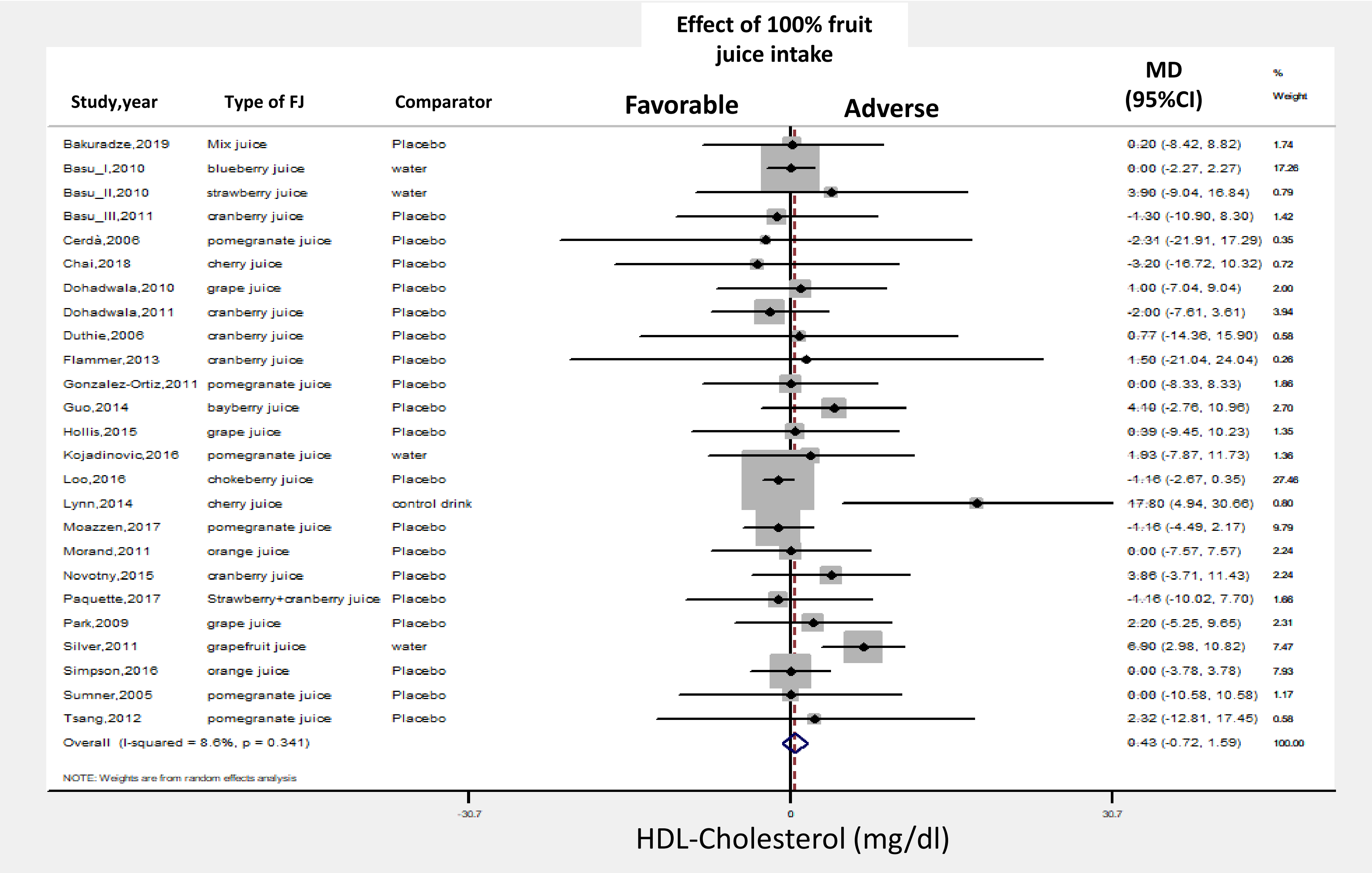

Supplemental Figure 14

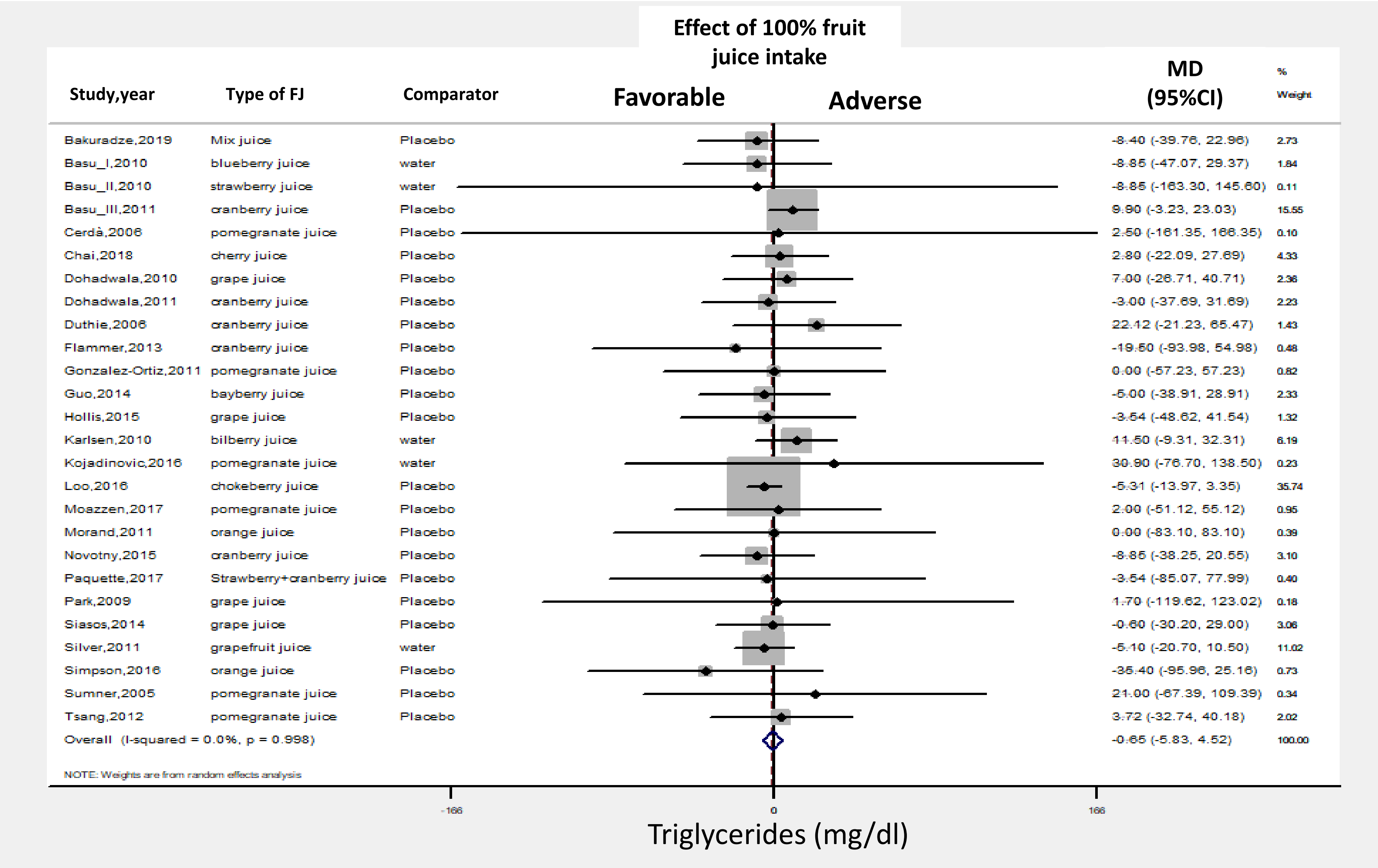

Supplemental Figure 15

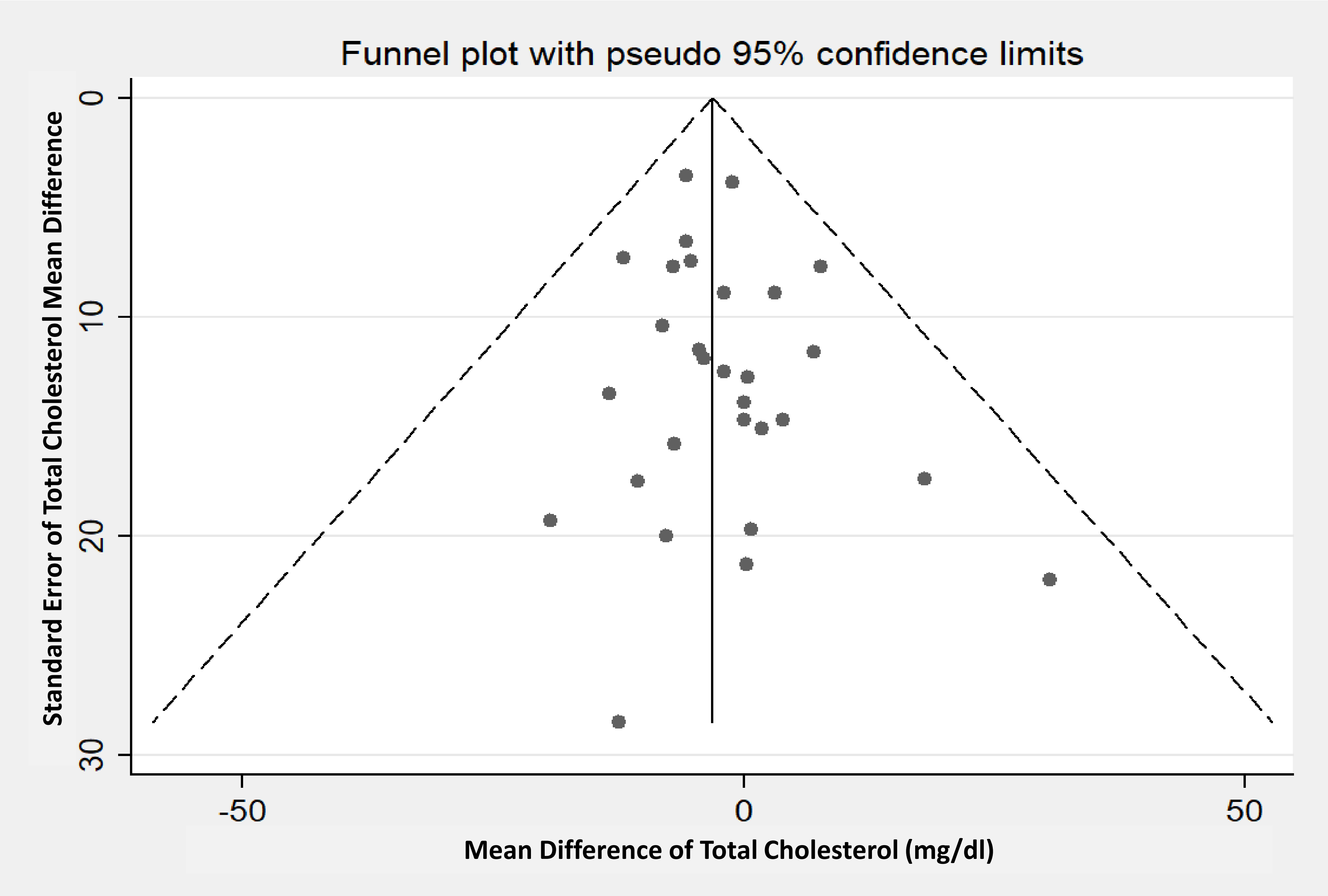

Supplemental Figure 16

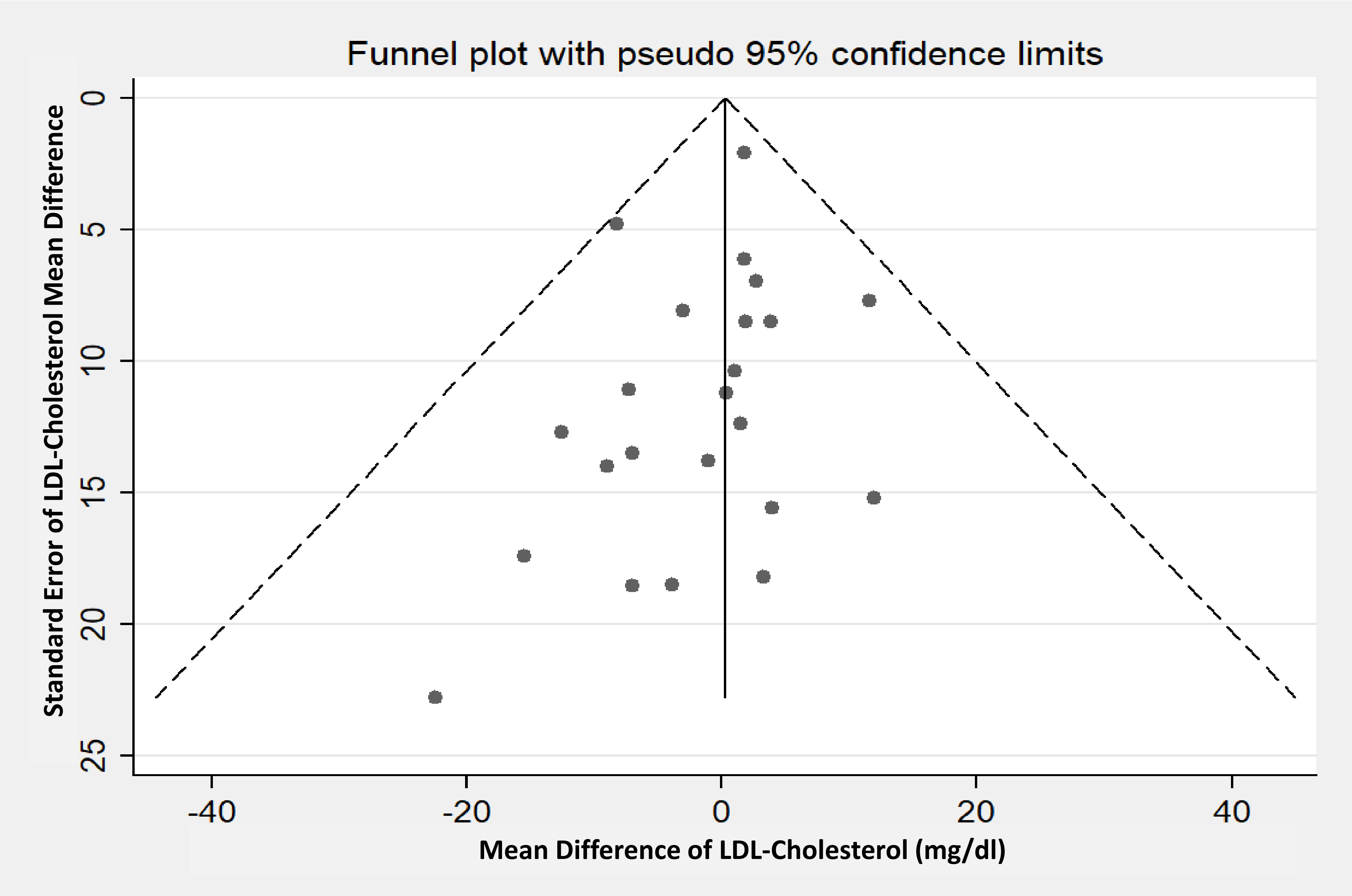

Supplemental Figure 17

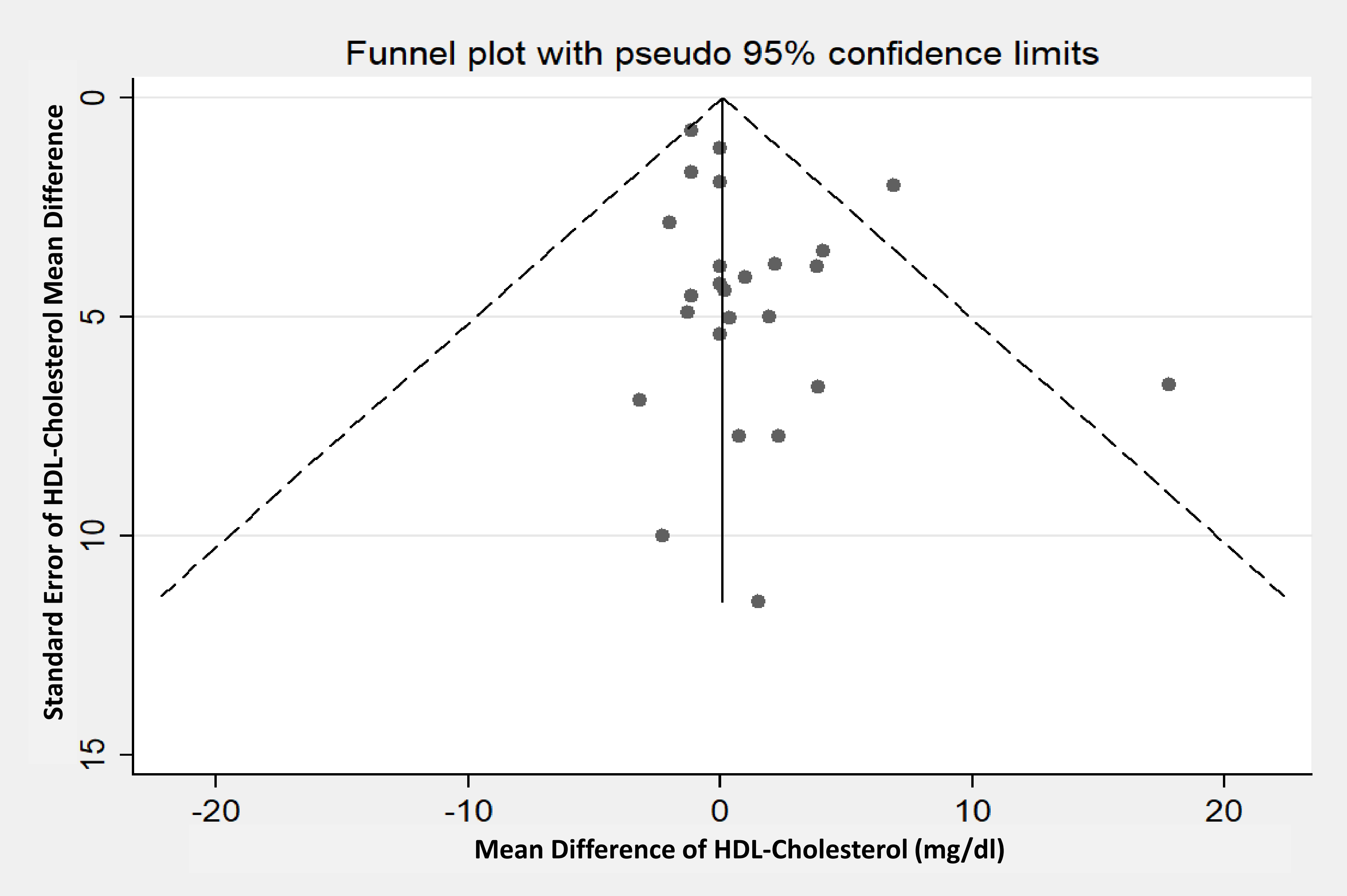

Supplemental Figure 18

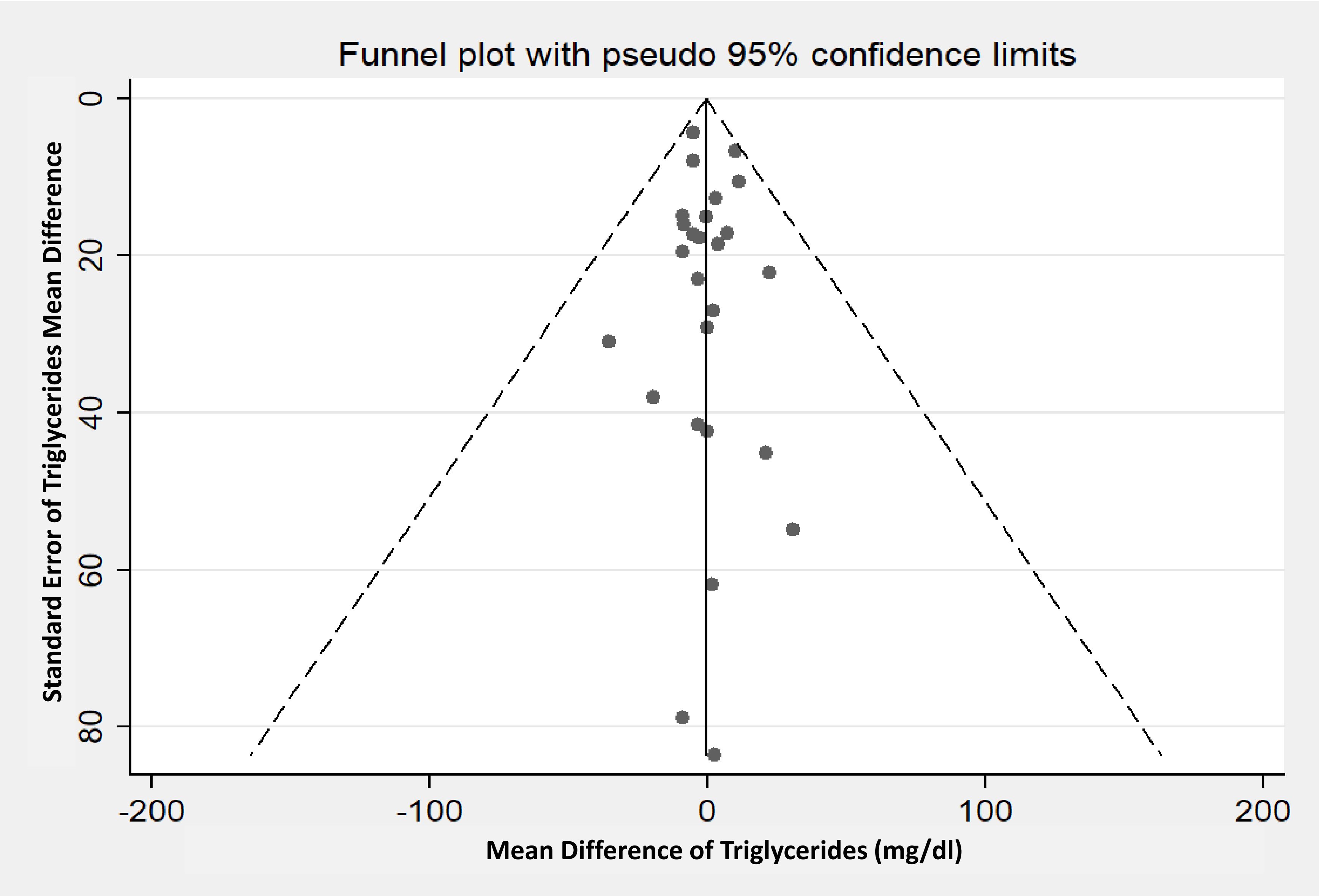

Supplemental Figure 19

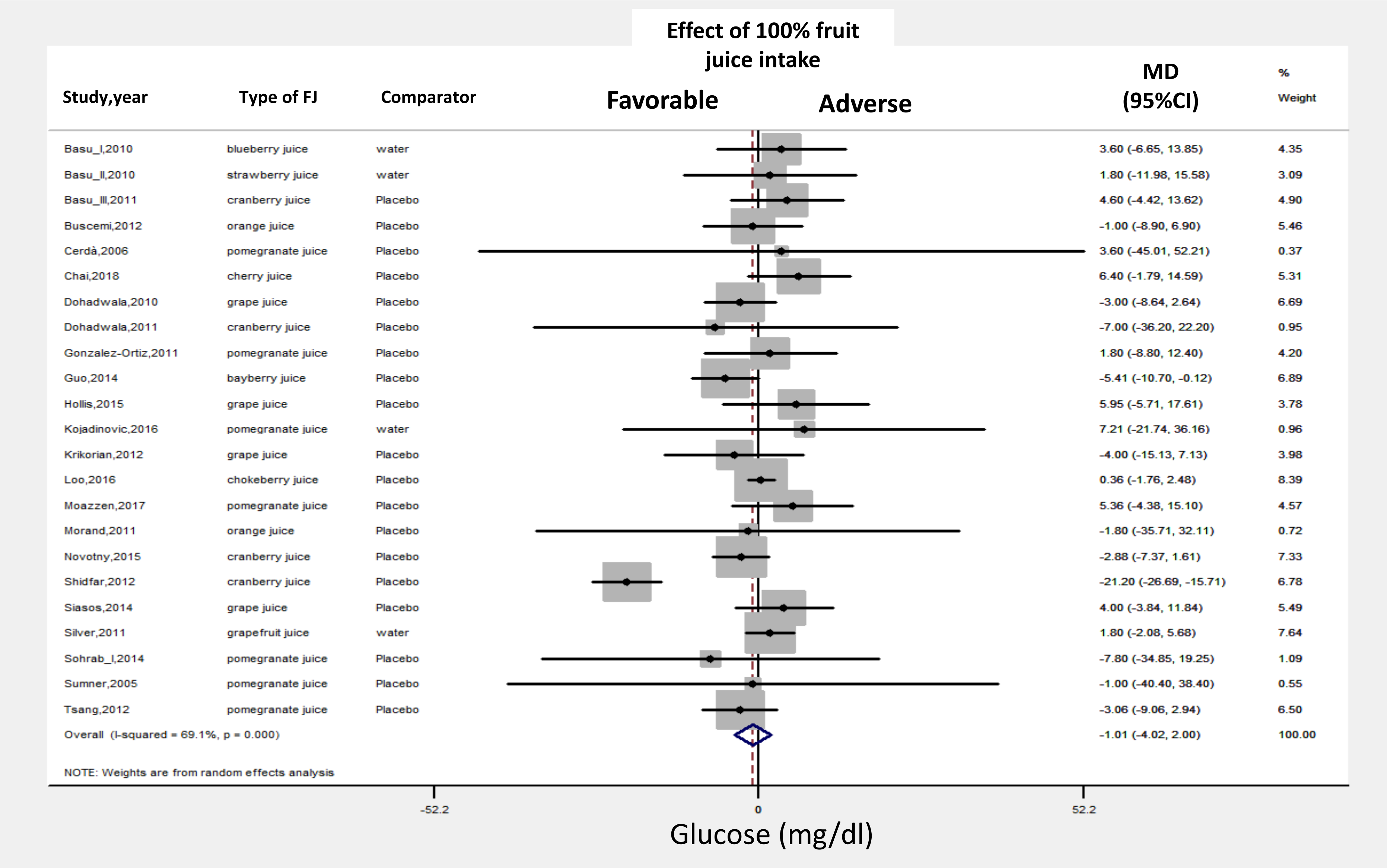

Supplemental Figure 20

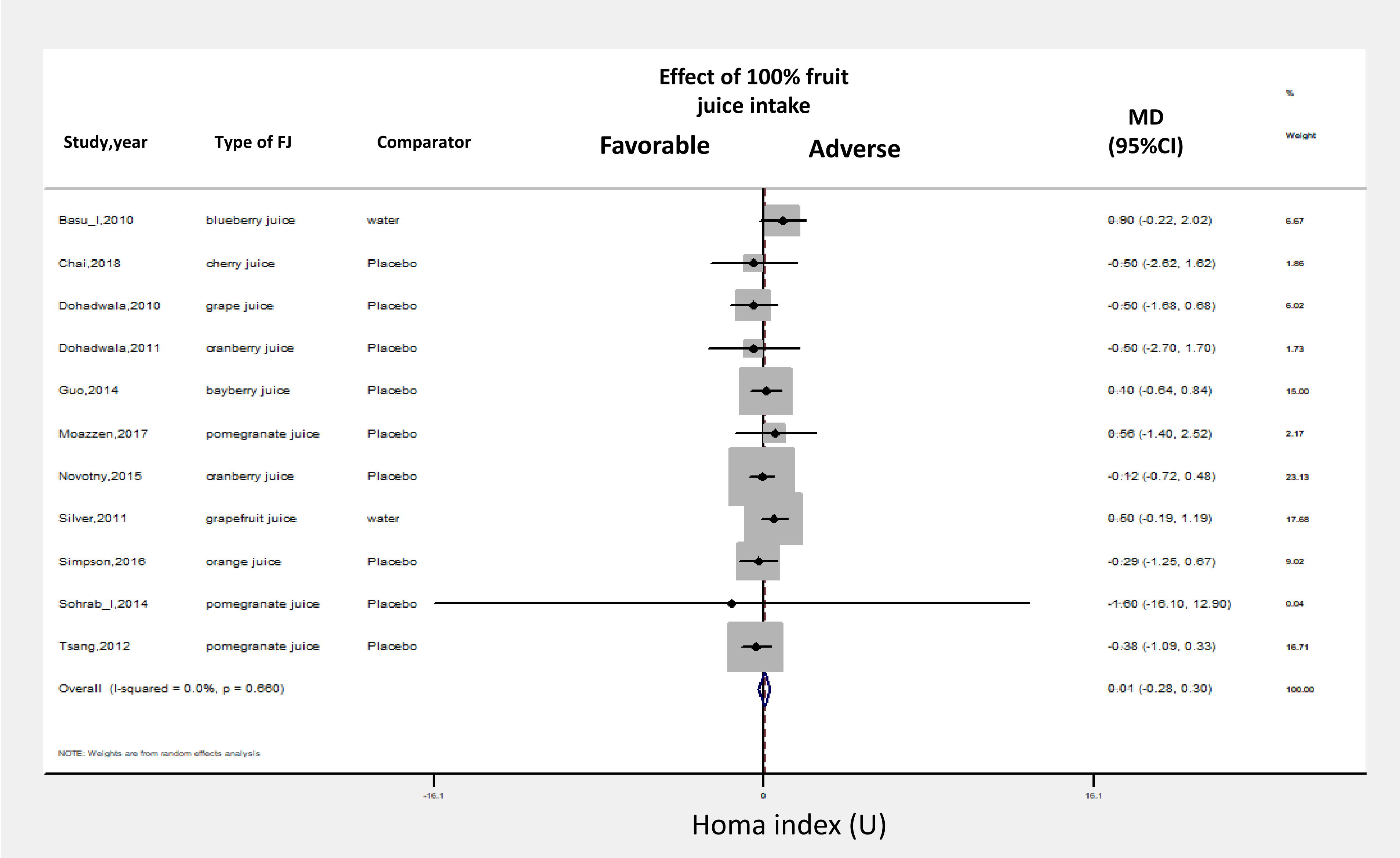

Supplemental Figure 21

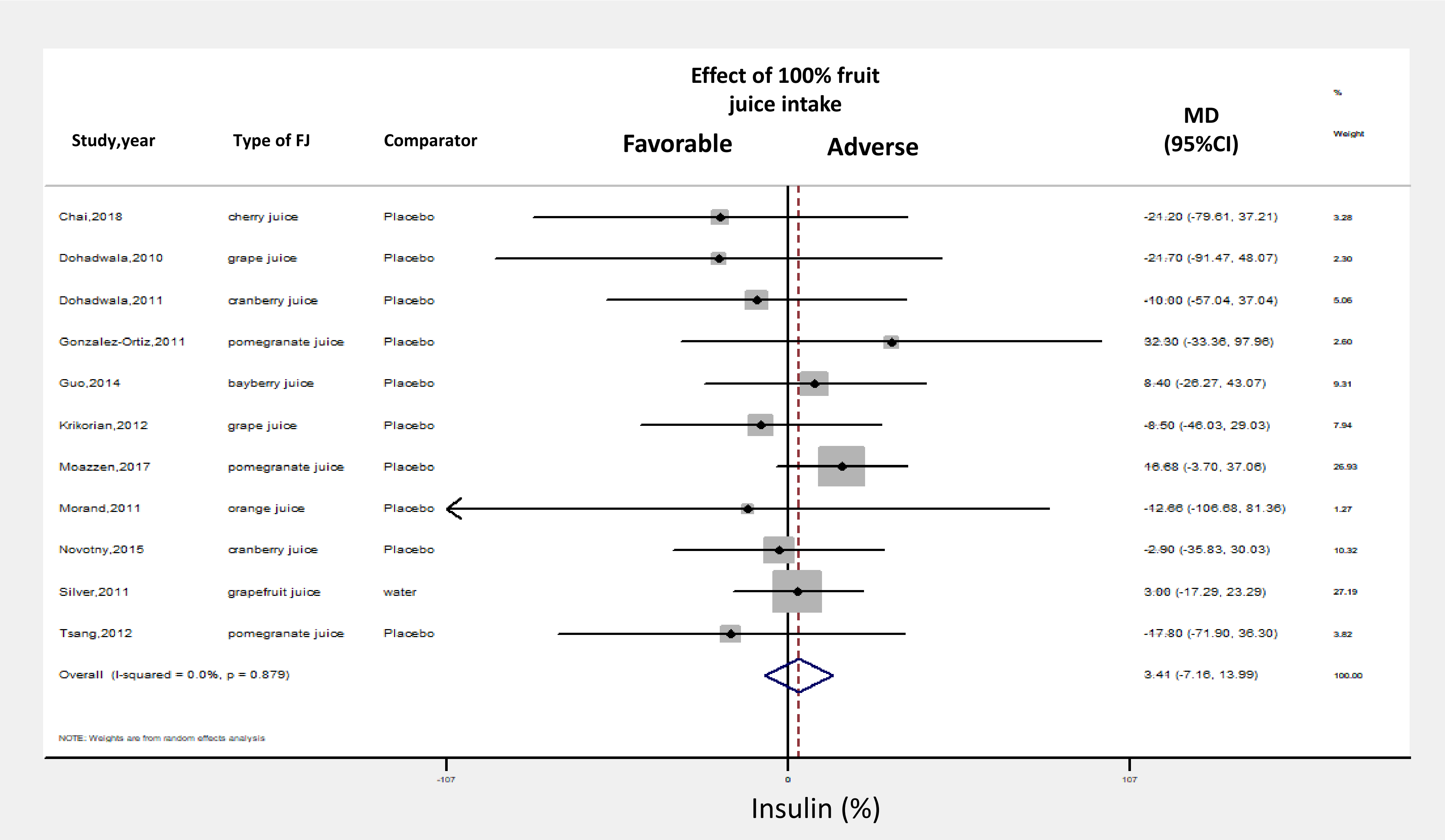

Supplemental Figure 22

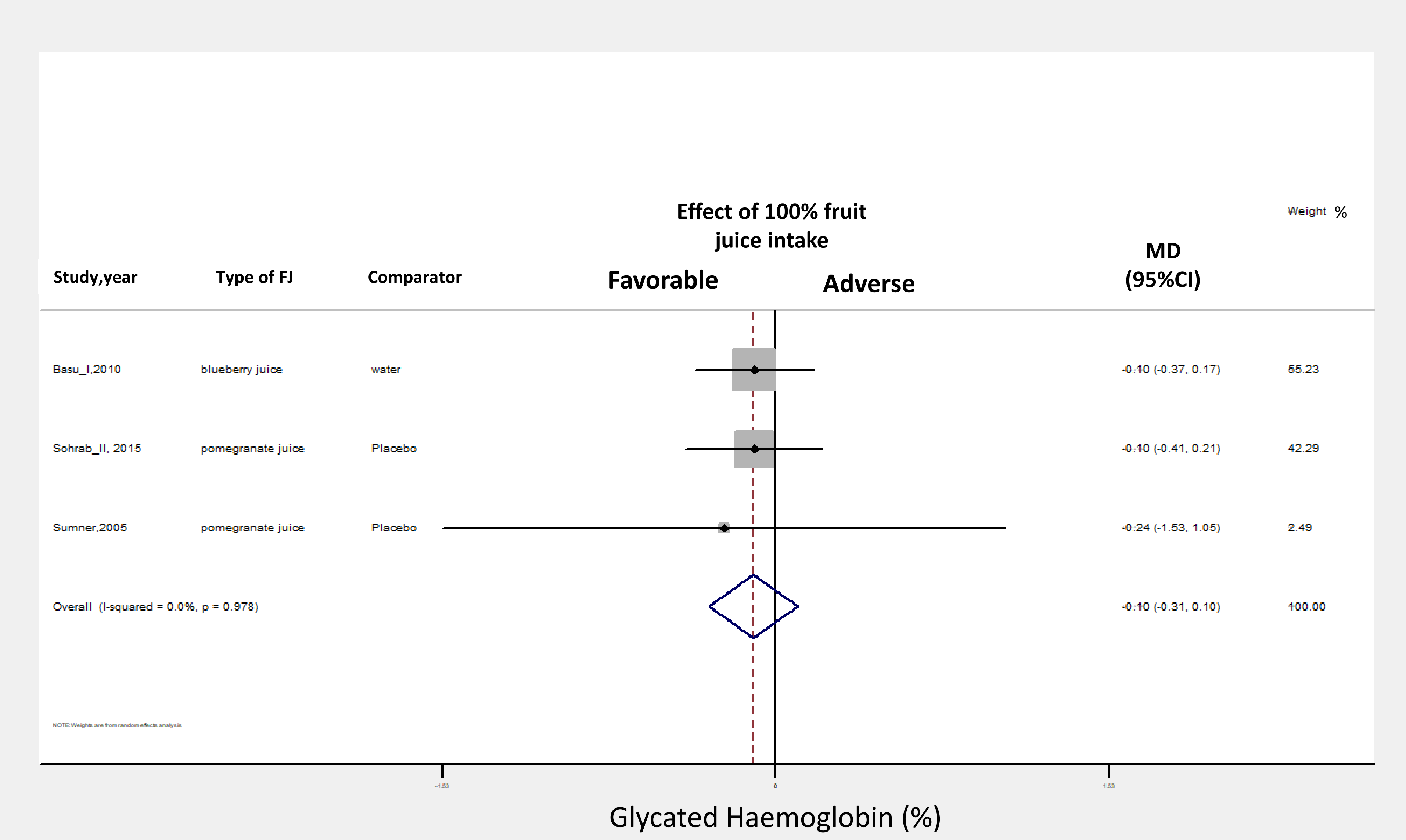

Supplemental Figure 23

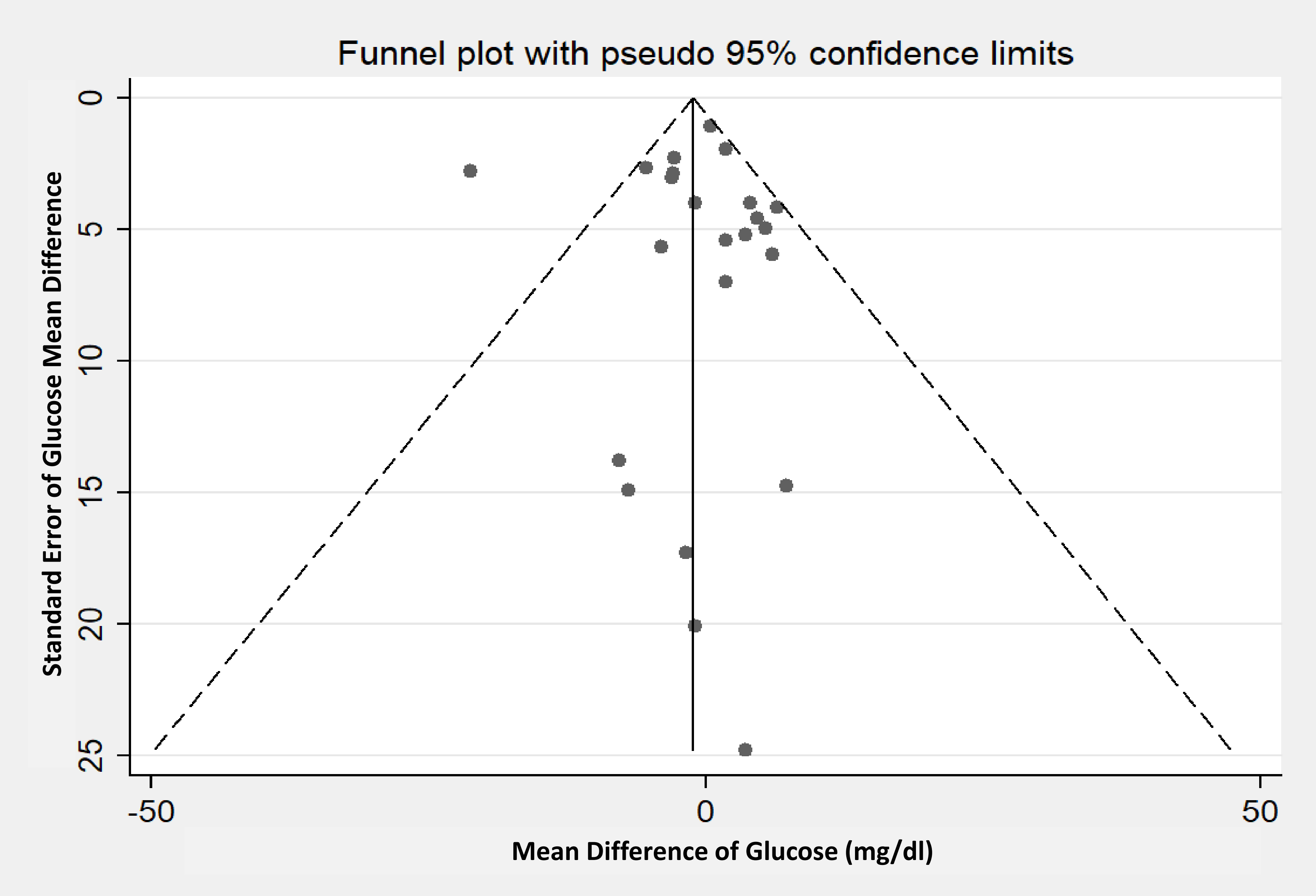

Supplemental Figure 24

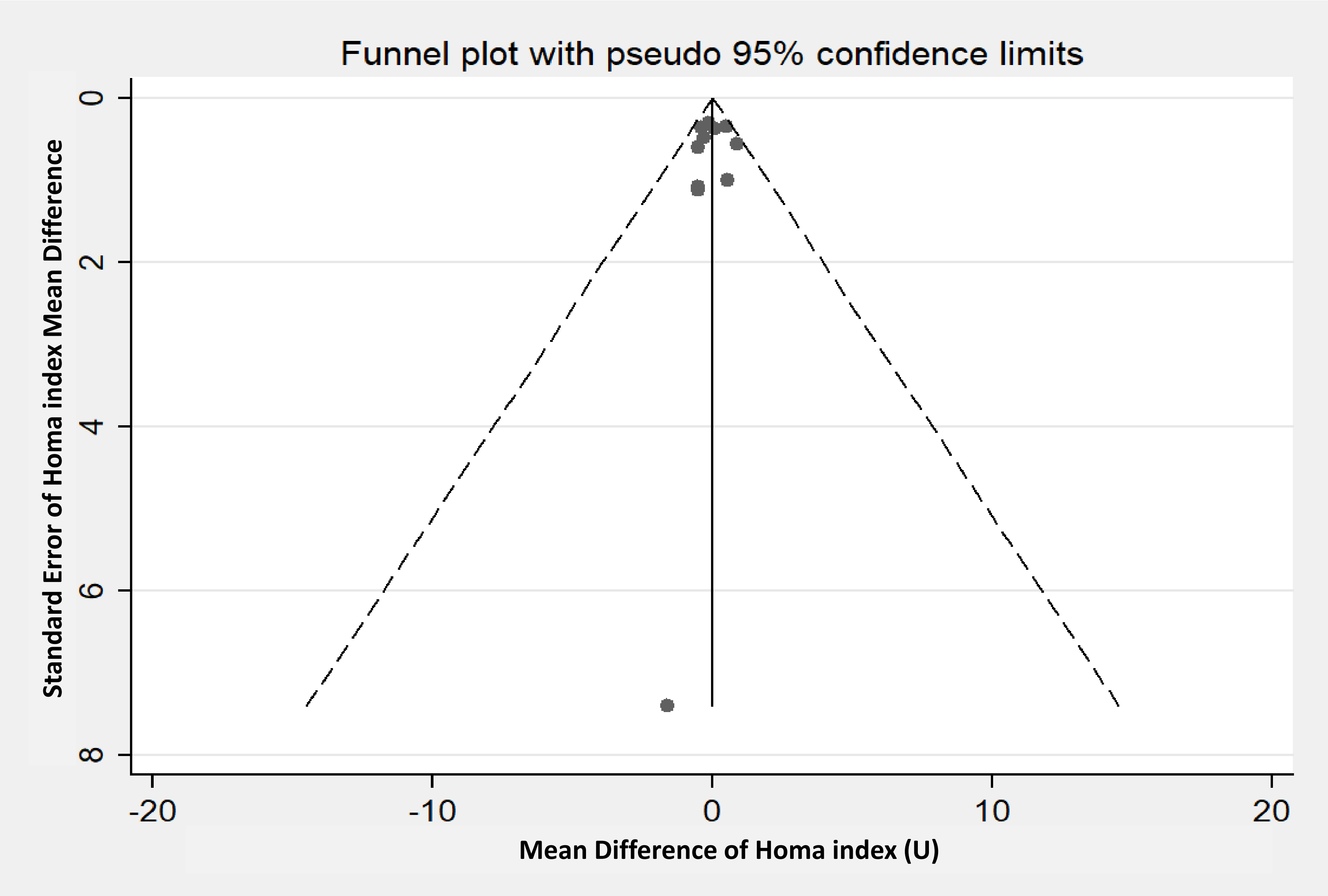

Supplemental Figure 25

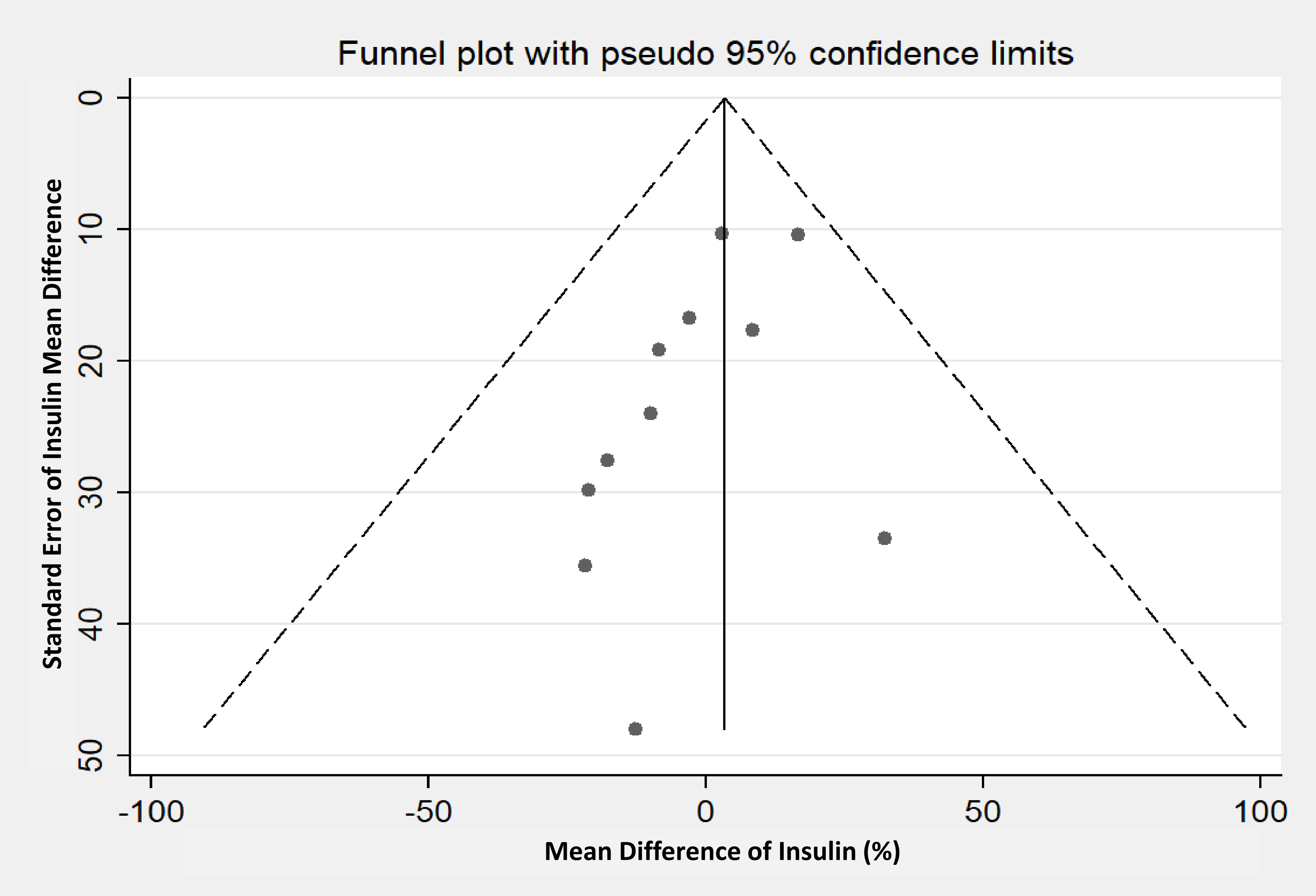

Supplemental Figure 26

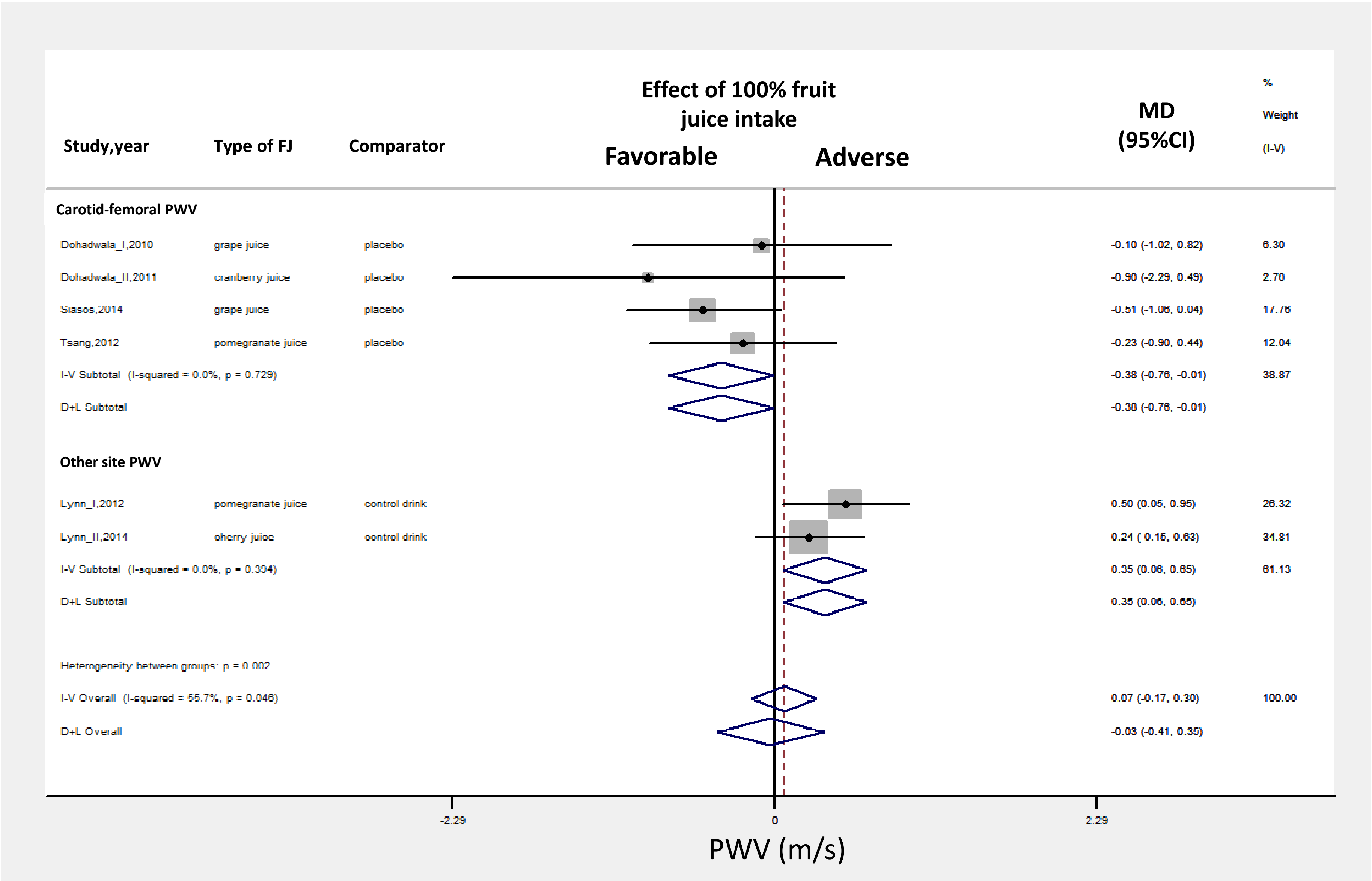

Supplemental Figure 27

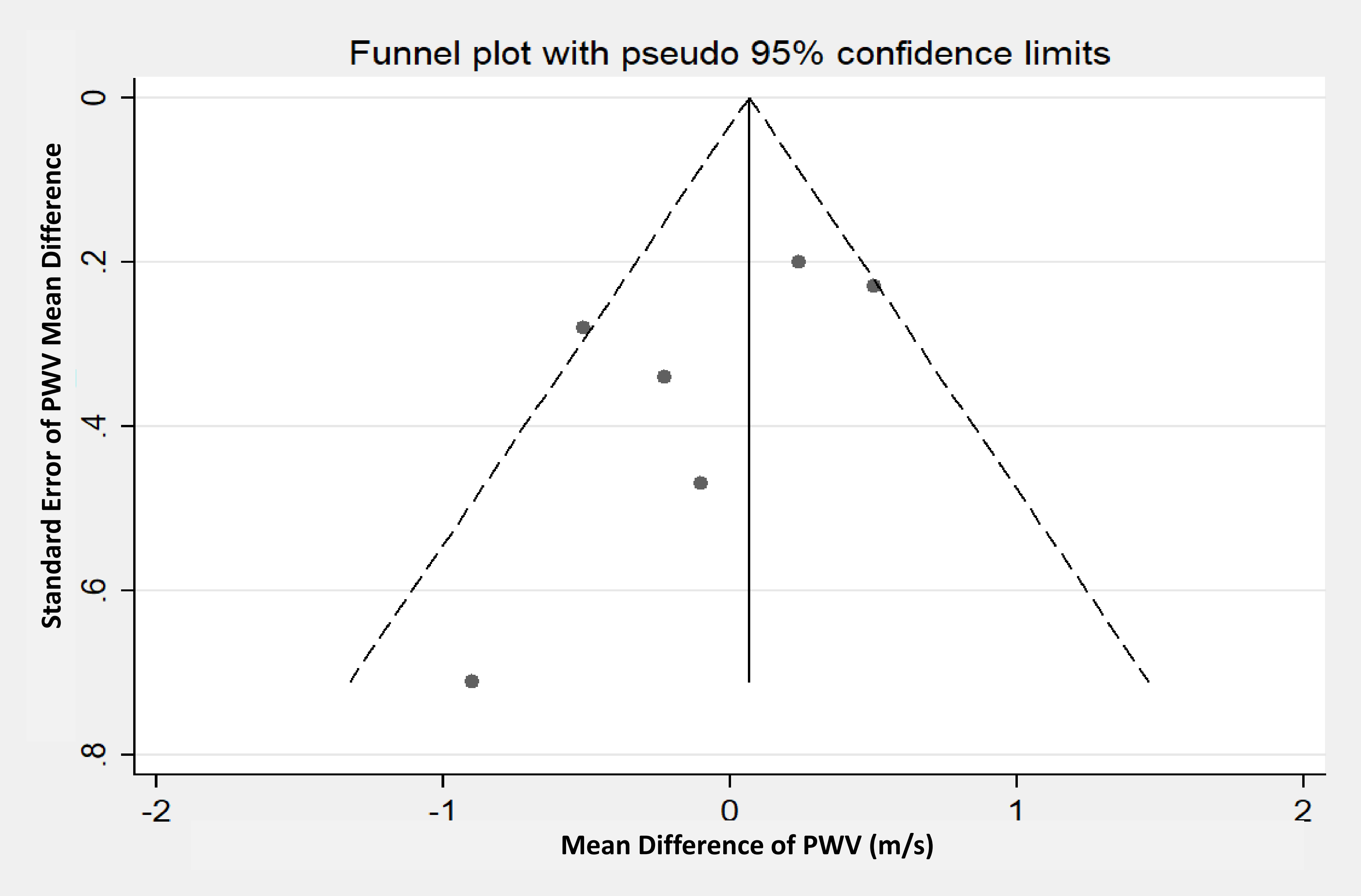

Supplemental Figure 28

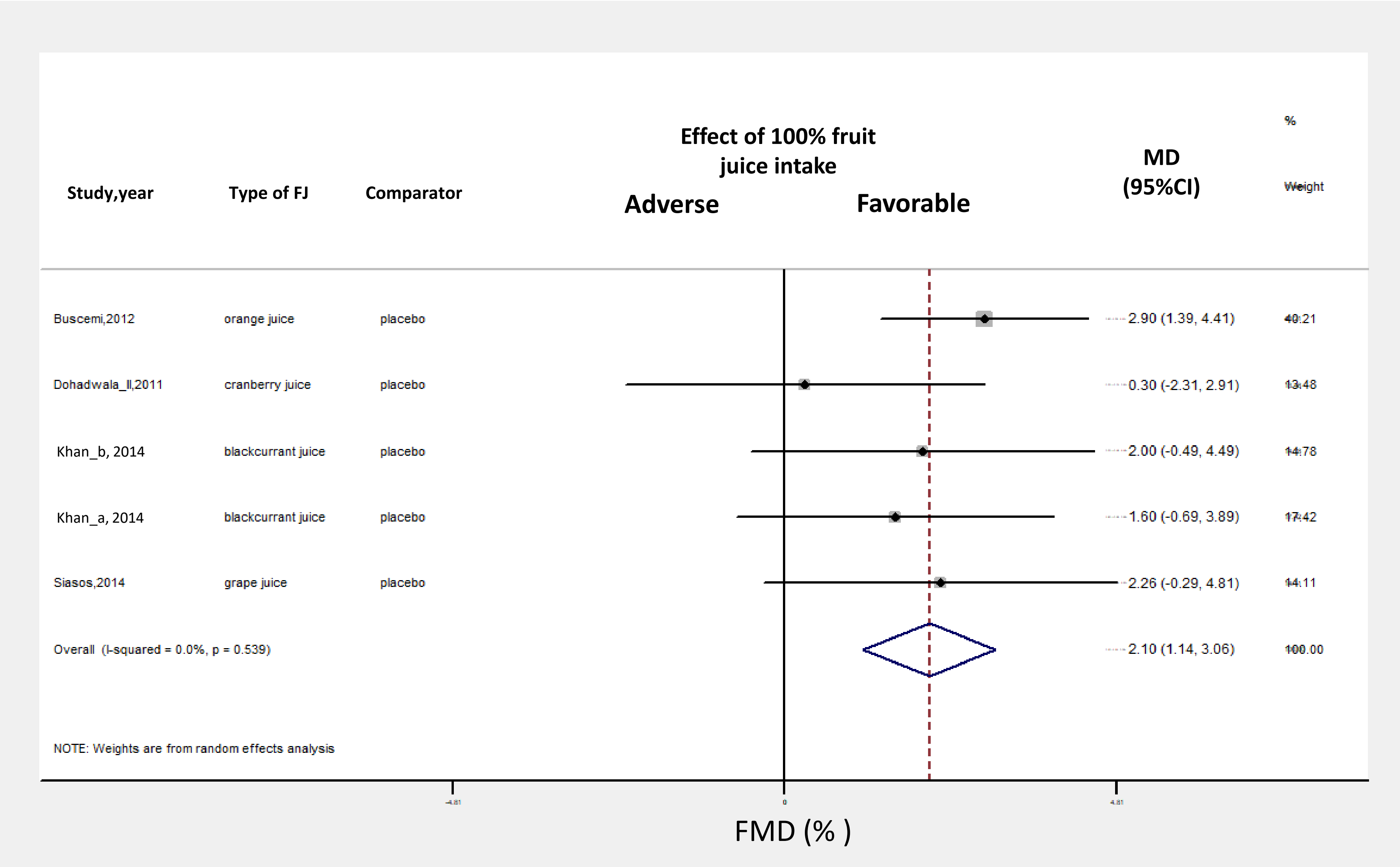

Supplemental Figure 29

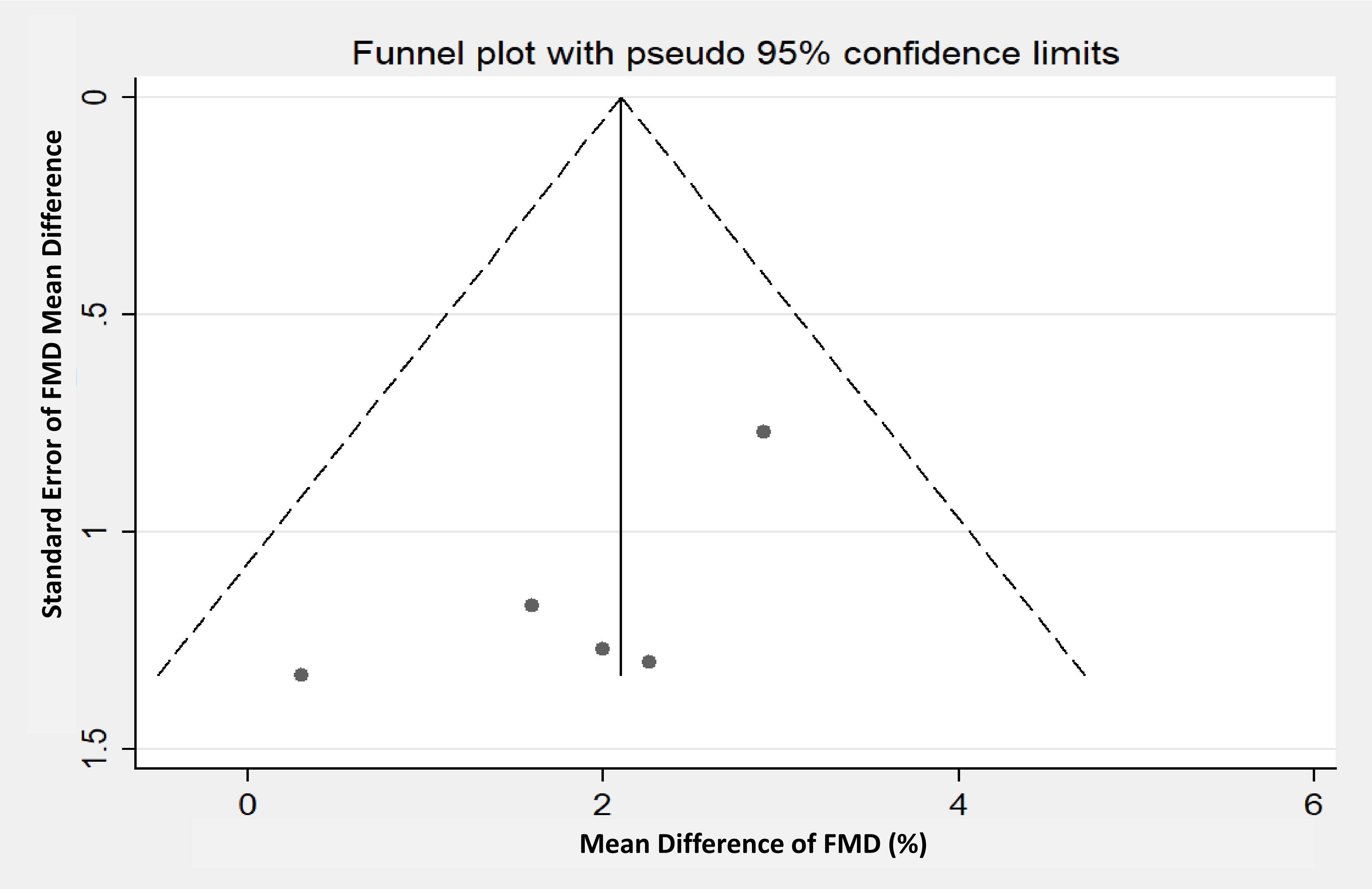

Supplement: Supplementary file 2 — Supplementary file2 (PDF 705 kb) [file 394_2020_2426_MOESM2_ESM.pdf]
